# The Identification of Phytohormone Receptor Homologs in Early Diverging Fungi Suggests a Role for Plant Sensing in Land Colonization by Fungi

**DOI:** 10.1128/mBio.01739-16

**Published:** 2017-01-31

**Authors:** Anaïs Hérivaux, Thomas Dugé de Bernonville, Christophe Roux, Marc Clastre, Vincent Courdavault, Amandine Gastebois, Jean-Philippe Bouchara, Timothy Y. James, Jean-Paul Latgé, Francis Martin, Nicolas Papon

**Affiliations:** a Université d’Angers, Groupe d’Etude des Interactions Hôte-Pathogène, Angers, France; b Université François-Rabelais de Tours, EA 2106, Biomolécules et Biotechnologies Végétales, Tours, France; c Laboratoire de Recherche en Sciences Végétales, Université de Toulouse, UPS, Castanet-Tolosan, France; d Department of Ecology and Evolutionary Biology, University of Michigan, Ann Arbor, Michigan, USA; e Institut Pasteur de Paris, Unité des Aspergillus, Paris, France; f Institut National de la Recherche Agronomique, Université de Lorraine, UMR 1136 Interactions Arbres/Microorganismes, Laboratoire d'Excellence ARBRE, Nancy, France; University of California

## Abstract

Histidine kinases (HKs) are among the most prominent sensing proteins studied in the kingdom Fungi. Their distribution and biological functions in early diverging fungi (EDF), however, remain elusive. We have taken advantage of recent genomic resources to elucidate whether relationships between the occurrence of specific HKs in some EDF and their respective habitat/lifestyle could be established. This led to the unexpected discovery of fungal HKs that share a high degree of similarity with receptors for plant hormones (ethylene and cytokinin). Importantly, these phytohormone receptor homologs are found not only in EDF that behave as plant root symbionts or endophytes but also in EDF species that colonize decaying plant material. We hypothesize that these particular sensing proteins promoted the interaction of EDF with plants, leading to the conquest of land by these ancestral fungi.

## OPINION/HYPOTHESIS

Histidine kinases (HKs) are prominent sensing proteins present in bacteria, amoebae, plants, and fungi. When activated, for instance following the perception of an external stimulus, HKs initiate more or less complex phosphorylation cascades, ranging from two-component systems (prevailing in bacteria) to multistep phosphorelays (in plants and fungi) that lead to an adapted response (1).

In bacteria, it is now well documented that HKs regulate a large panel of fundamental processes, including nutrient acquisition, various metabolic activities, adaptation to changes in the environment, developmental pathways, virulence, antibiotic resistance, and many others (2). In plants, HKs act as osmosensors by regulating responses to drought, salt stress, and stomatal closure, but more importantly they have been implicated in the perception of two major phytohormones, cytokinins and ethylene (3). HKs are also widespread in the kingdom Fungi, and to date in Dikarya (i.e*., Ascomycota* and *Basidiomycota*) they have been reported to be involved in stress adaptation, red light perception, morphogenesis, and virulence (4). With the exception of a few recent insights into the distribution of these sensing proteins in *Mucoromycotina* (5), no extensive analysis of HKs has been conducted to date across the so-called early diverging fungal (EDF) lineages, which comprise a large portion of the phylogenetic diversity of the kingdom Fungi though just a small proportion of described species (6–8).

**FIG 1:**
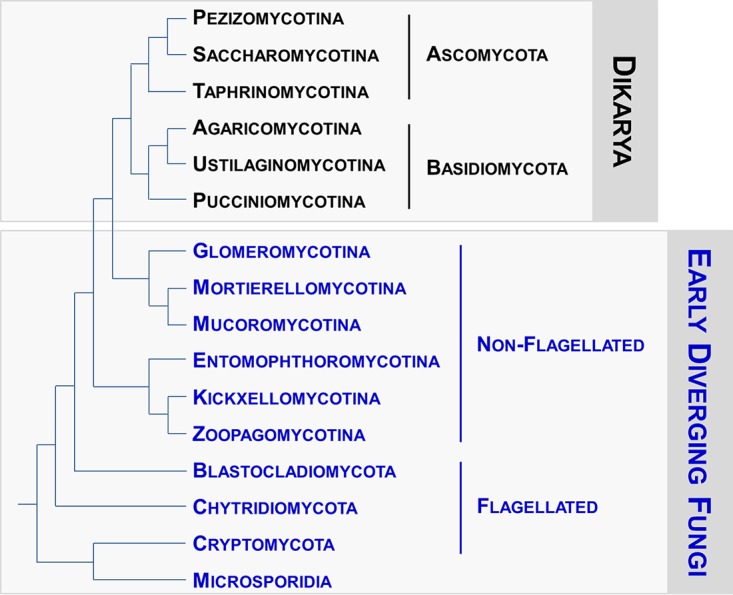
Phylogeny of the kingdom Fungi. The 10 early diverging fungal lineages are indicated in blue. The topologies resemble the current understanding of the relationships of the fungal groups, according to reference 9.

EDF are currently categorized into 10 lineages (depicted in Fig. 1) (9). From an ecological point of view, the habitats of EDF are broadly diversified, ranging from marine or freshwater environments to soils, with more or less tight associations with plants. Many other EDF lifestyles require strong interactions with hosts, as illustrated by *Glomeromycotina*, which live exclusively as obligate symbionts of vascular plants, bryophytes, and cyanobacteria, whereas several EDF have been identified as insect or vertebrate pathogens (6–8). In this way, to survive in a wide range of ecological niches or to fine-tune interactions with their respective hosts, EDF have likely developed a variety of cell signaling strategies that allow them to perceive and to cope appropriately with a broad range of external cues. Since HKs remain among the most important sensing proteins in Dikarya (1), we were primarily interested for this study in exploring the structure and distribution of HKs in EDF and in elucidating whether relationships could be established between the occurrence of particular HKs in some species/groups and their respective habitats and lifestyles.

## EXPLORING THE STRUCTURAL DIVERSITY AND DISTRIBUTION OF HKs AMONG THE EDF LINEAGES

**FIG 2:**
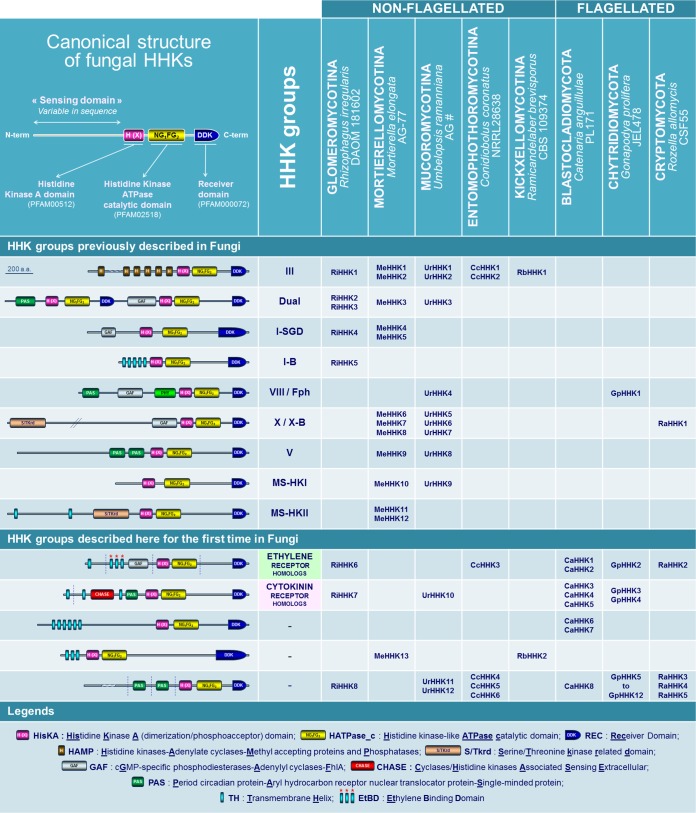
Compilation of HHKs in some early diverging fungi. Gene loci encoding HHKs in EDF genomes were identified following multiple tBLASTn and BLASTp searches against selected genomes of the Joint Genome Institute (JGI) MycoCosm database (6) (http://genome.jgi.doe.gov/programs/fungi/index.jsf). Conserved Domain Database (CDD) sequences for HisKA (PFAM00512), HATPase_c (PFAM02518), and REC (PFAM00072) were used in BLAST searches of each EDF genome. All hits producing *E* values below 10^−4^ were further analyzed. Functional domains were identified with CDD, and predictions of transmembrane-spanning regions were carried out using TMHMM v2.0. All sequences are compiled in Text S1 in the supplemental material.

The basic structure of fungal HKs is now well established (Fig. 2, top left panel). In contrast to most bacterial HKs, the histidine kinase A (HisKA) and histidine kinase ATPase catalytic (HATPase_c) domains are fused to the C-terminal receiver domain (REC); thus, importantly, fungal HKs are generically defined as hybrid HKs (HHKs). It is worth noting that the variable N-terminal region, referred to as the sensing domain, displays a combination of motifs that drives the perception properties of the HHK (Fig. 2, top left panel) (10). Based on the sequence analysis of both histidine kinase A and sensing domains from more than 200 predicted proteins, fungal HHKs are currently categorized into 16 groups (4).

10.1128/mBio.01739-16.1 Text S1  Further description of methods used for the phylogenetic analysis and a compilation of nucleotide sequences used in the study. Download Text S1, DOCX file, 0.1 MB. Copyright © 2017 Hérivaux et al. 2017 Hérivaux et al. This content is distributed under the terms of the Creative Commons Attribution 4.0 International license.

To gain insight into the structure and distribution of these proteins in the EDF lineages, we were primarily interested in compiling predicted sequences that bear the HHK canonical domains described in top left panel of Fig. 2 (10). For this purpose, we browsed the genome of a representative species from each EDF lineage (Fig. 1); the characteristics of these species are summarized in Table 1. Due to the lack of genomic resources for *Zoopagomycotina*, this lineage was not incorporated into the present analysis, nor were the *Microsporidia*, which do not contain HHK-encoding genes in their genome.

**TABLE 1:** Early diverging fungi whose genomes were examined in this study

Species^a^	Strain	Fungal lineage	Lifestyle or habitat^b^
Preliminary exploration of HHK structures			
* Rhizophagus irregularis*	DAOM 181602	*Glomeromycotina* (nonflagellated)	Obligate endosymbiont of plant roots
* Mortierella elongata*	AG-77	*Mortierellomycotina* (nonflagellated)	Saprotrophic, widely distributed in soils
* Umbelopsis ramanniana*	AG #	*Mucoromycotina* (nonflagellated)	Saprotrophic, facultative endophyte in woody roots
* Conidiobolus coronatus*	NRRL28638	*Entomophthoromycotina* (nonflagellated)	Saprotrophic, rarely parasite of insects and mammals
* Ramicandelaber brevisporus*	CBS 109374	*Kickxellomycotina* (nonflagellated)	Saprotrophic, widely distributed in soils
* Catenaria anguillulae*	PL171	*Blastocladiomycota* (flagellated)	Saprotrophic, decaying plant materials, facultative parasite of plant pathogenic nematodes
* Gonapodya prolifera*	JEL478	*Chytridiomycota* (flagellated)	Saprotrophic, decaying plant material
* Rozella allomycis*	CSF55	*Cryptomycota* (flagellated)	Obligate parasite of *Allomyces* *macrogynus*
Secondary exploration^b^			
* Gigaspora rosea*	DAOM194757	*Glomeromycotina* (nonflagellated)	Obligate endosymbiont of plant roots
* Basidiobolus meristosporus*	CBS 931.73	*Entomophthoromycotina* (nonflagellated)	Saprotrophic, decaying plant material
* Zoophthora radicans*	ARSEF 4784	*Entomophthoromycotina* (nonflagellated)	Parasite of insects
* Allomyces macrogynus*	ATCC 38327	*Blastocladiomycota* (flagellated)	Saprotrophic, decaying plant material
* Spizellomyces punctatus*	DAOM BR117	*Chytridiomycota* (flagellated)	Saprotrophic, decaying plant material
* Batrachochytrium dendrobatidis*	JAM81	*Chytridiomycota* (flagellated)	Parasite of amphibians
* Rhizoclosmatium globosum*	JEL800	*Chytridiomycota* (flagellated)	Saprotrophic
* Piromyces* sp.	E2	*Neocallimastigomycetes* (flagellated)	Mutualist in gut in variety of herbivores

a All genomes were compared with information from the Joint Genome Institute MycoCosm database (6) (http://genome.jgi.doe.gov/programs/fungi/index.jsf). See Fig. 2 for further information on the genomes browsed for the preliminary exploration of HHK structures; for information regarding the genomes browsed in the secondary exploration, see Fig. 3, 4, and 6.

b Several genome sequences used in this study are included in this table: *Rhizophagus irregularis*, *Rozella allomycis*, *Batrachochytrium dendrorabatidis*, *Gigaspora rosea*, and *Spizellomyces punctatus* (31–35).

A compilation of HHK structures deduced from EDF genomes is provided in Fig. 2. Above all, this compilation gives evidence that 9 out of the 16 fungal HHK groups previously identified in Dikarya are also present in EDF. This includes, notably, the osmosensing group III HHKs, the dual HHK group, which was initially thought to be restricted to *Basidiomycota*, and the red light sensing phytochromes (the VIII/Fph group) (4, 11). Interestingly, some unprecedented HHKs are also scattered among the different EDF lineages (Fig. 2), and it was particularly surprising that most of their sensing domains harbor hydrophobic transmembrane helices, distinguishing them from the majority of Dikarya-related HHKs (4).

## FIRST DESCRIPTION OF PLANT HORMONE RECEPTOR HOMOLOGS IN EDF

The striking finding of this analysis is actually the discovery of fungal HHKs with a high degree of similarity with two groups of plant hormone receptors, i.e., ethylene and cytokinin receptors (Fig. 2). Both phytohormones are known to play crucial roles in plant development, and recent works have highlighted a cytokinin/ethylene interaction at diverse levels of biosynthetic and metabolic pathways (3). From a general point of view, ethylene and cytokinins are also well-documented as key signaling molecules in plant biotic interactions (with viruses, protists, bacteria, worms, insects, and fungi). Importantly, recent advances have revealed that in several plant-fungus systems, both plant- and microorganism-borne phytohormones have concerted effects that promote interactions (12–17).

## SOME EDF GENOMES ENCODE ETHYLENE RECEPTOR HOMOLOGS

We first identified new fungal HHKs that share strong identities with plant ethylene receptors (Fig. 2 and 3A). The main feature that differentiates plant ethylene receptors from other bacterial or fungal HKs is the presence within the N-terminal sensing region of an ethylene binding domain consisting of a combination of three transmembrane helices that bear conserved amino acids essential for hormone perception (Fig. 3B) (18). We identified this particular N-terminal feature initially in the *Rhizophagus* (*Glomeromycotina*) RiHHK6 predicted protein (Fig. 2). *Glomeromycotina* form arbuscular mycorrhizae with plants (Table 1), and the importance of ethylene in the establishment of this type of symbiosis has been previously demonstrated (12, 13). Further BLAST analysis of more than 500 fungal genomes (using the *Rhizophagus* RiHHK6 ethylene binding domain as the query) led us to identify homologous sequences in several other EDF which are known to colonize plant materials (leaf litter, twigs, decaying fruits, soil) (Table 1), including *Conidiobolus* (CcHHK3), *Catenaria* (CaHHK1 and CaHHK2), *Gonapodya* (GpHHK2), *Basidiobolus* (Bm|388937|), and *Spizellomyces* (SPPG_07928) (Fig. 2 and 3A). We also noticed the presence of ethylene receptor homologs in *Allomyces* (AMAG_07677, AMAG_07095, AMAG_07058, and AMAG_09825) and *Rozella* (RaHHK2) (Fig. 2 and 3A). Since it is well-known that *Rozella* behaves as an obligate parasite of *Allomyces* (19), it could be hypothesized that ethylene orchestrates interactions between these two aquatic species. Alternatively, the presence of ethylene receptor homologs in these two flagellated fungi could be inherent to their location in the deepest branches of the tree and the inheritance of ethylene receptor homologs in the common ancestor of all fungi (9). All these new EDF proteins are depicted in Fig. 3A, along with several previously characterized ethylene receptors from plants, green algae, and cyanobacteria (20). As revealed by alignment of the ethylene binding domains from these proteins (Fig. 3B), all homologs to ethylene receptors identified in the genomes of the set of EDF display the three predicted transmembrane helices containing all of the crucial residues involved in ethylene perception (18). Plant ethylene receptors are currently categorized in two subfamilies: subfamily 1 includes members displaying an ethylene binding domain together with a GAF domain (cyclic **G**MP-specific phosphodiesterases/**a**denylyl cyclases/**F**hlA domain) in the sensing region, whereas subfamily 2 includes ethylene receptors containing a supplemental N-terminal transmembrane helix, compared to subfamily 1 members (21). As shown in Fig. 3A, both subfamilies are represented among EDF. In addition, it is now recognized that some members of the *Arabidopsis* ethylene receptor series lack several HHK functional domains, such as the C-terminal receiver domain (REC; AtERS1 and AtERS2) up to the histidine kinase ATPase catalytic (HATPase_c) domain (AtEIN4, AtERS2, and AtETR2) (21). Interestingly, such truncated features are also observed in some *Entomophthoromycotina* (*Conidiobolus* CcHHK3 and *Basidiobolus* Bm|388937|) (Fig. 3A).

**FIG 3:**
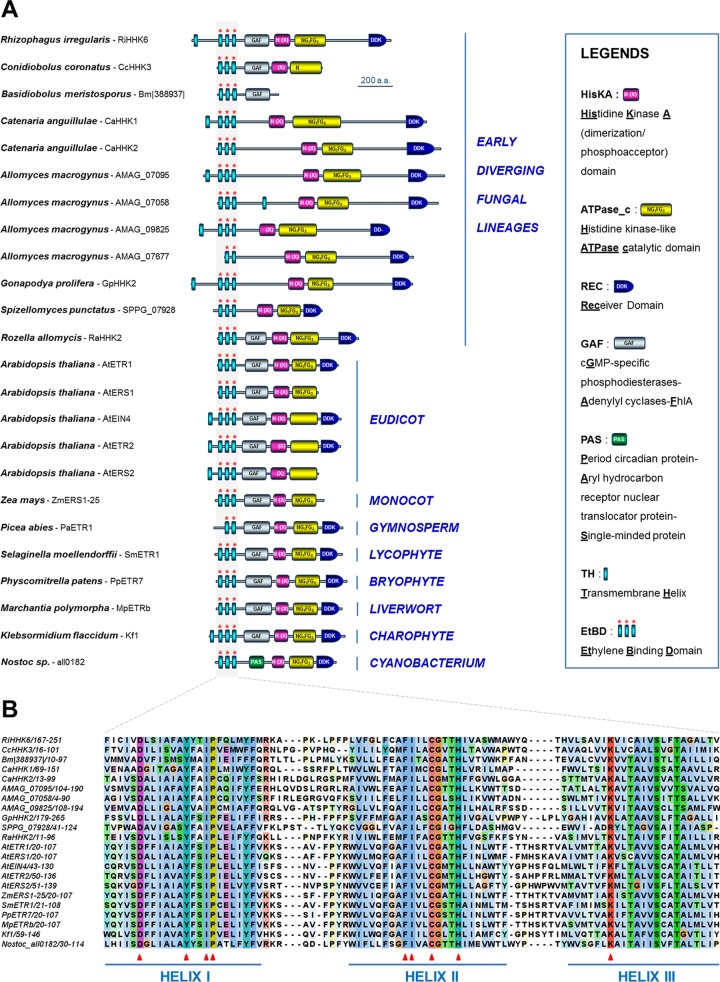
Structures of homologs to ethylene receptors identified in early diverging fungi. (A) Diversity of structures found in various early diverging fungi. The structures of several previously characterized ethylene receptors from plant, green algae, and cyanobacteria are also provided (20). (B) Alignment of ethylene binding domains from homologs to ethylene receptors identified in early diverging fungi, along with others from plant, algae, and cyanobacteria. The ethylene binding domains consist of three transmembrane helices. Essential residues that have been reported to be involved in ethylene perception (18) are indicated with red arrows. AMAG_07677 and PaETR1 sequences were not included in this analysis (incomplete ethylene binding domain). The amino acid sequence alignment was formatted with the JALVIEW program.

## SOME EDF GENOMES ENCODE CYTOKININ RECEPTOR HOMOLOGS

**FIG 4:**
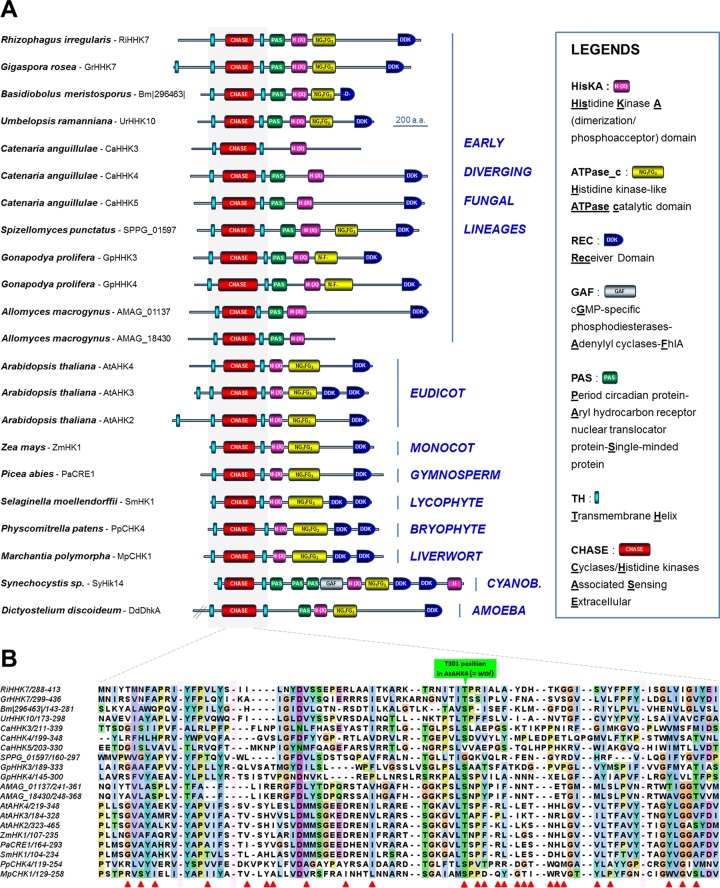
Structures of homologs to cytokinin receptors identified in early diverging fungi. (A) Diversity of structures found in various early diverging fungi. The structures of several previously characterized plant cytokinin receptors (23) and other CHASE domain-containing HHKs (which had not been demonstrated to date to act as cytokinin receptors) from *Synechocystis* sp. (Cyanobacteria) and *Dictyostelium discoideum* (Amoebae) are also provided. (B) Alignment of CHASE domains from homologs to cytokinin receptors identified in early diverging fungi, along with others from plant cytokinin receptors. The *Dictyostelium* and *Synechocystis* sequences were not included in this analysis because their subgroup was not supported by a high bootstrap value. Interruptions of the alignments are indicated by pink rectangles, and essential residues reported to be involved in cytokinin perception in *A. thaliana* AtAHK4 are indicated with red arrows (previously compiled in reference 23).

When exploring the structural diversity of HHKs in EDF, we were also surprised to observe unprecedented fungal HHKs that bear within their N terminus a CHASE domain (**c**yclases/**h**istidine kinase-**a**ssociated **s**ensing **e**xtracellular domain) surrounded by two predicted transmembrane helices (Fig. 2). This remains indeed the characteristic feature of plant cytokinin receptors (22, 23). These CHASE domain-containing HHKs among the kingdom Fungi were initially identified in this study among the *Glomeromycotina* (*Rhizophagus* RiHHK7 and *Gigaspora* GrHHK7) (Fig. 2 and 4A). As for ethylene, the major importance of the host plant cytokinins in the development of arbuscular mycorrhizal symbiosis by *Glomeromycotina* is now well documented (15, 16). Further investigations (BLAST analysis using the *Rhizophagus* RiHHK7 CHASE sequence as the query) allowed us to identify homologous sequences in other EDF which, interestingly, are known to colonize decaying plant material (Table 1), including the genera *Catenaria* (CaHHK3, CaHHK4, and CaHHK5), *Gonapodya* (GpHHK3, GpHHK4), *Basidiobolus* (Bm|296463|), *Allomyces* (AMAG_01137, AMAG_18430), and *Spizellomyces* (SPPG_01597) (Fig. 4A). In *Mucoromycotina*, cytokinin receptor homologs are also found in the genus *Umbelopsis* (UrHHK10) (Fig. 4A) but not in the other 30 *Mucoromycotina* species for which the genome sequences are available. *Umbelopsis* species display a unique lifestyle compared to other *Mucoromycotina* (predominantly including saprotrophic or pathogenic species), as *Umbelopsis* spp. have been reported as endophytes in root xylem tissues (Table 1) (24). Figure 4A depicts this set of new fungal HHKs, along with the structure of several plant cytokinin receptors and CHASE domain-containing HHKs. As previously described for plant cytokinin receptors (22, 23), alignment of all these predicted protein sequences revealed that the CHASE domain remains highly degenerated (Fig. 4B). This suggests that EDF may sense numerous cytokinin derivatives, as observed in plants that typically contain a mixture of different biologically active cytokinin metabolites (25). Interestingly, the most important amino acid for hormone binding in the *Arabidopsis* AHK4 cytokinin receptor, i.e., threonine 301 (green arrow in Fig. 4B), is conserved or similar (serine) in almost all the CHASE domain-containing HHKs deduced from EDF genomes. Indeed, this residue was at the origin of the discovery of plant cytokinin receptors, since when mutated in AHK4, this led to the *wooden leg (wol)* mutant phenotype in *Arabidopsis* (an altered root morphology characteristic of the absence of cytokinin perception in plants) (26).

## DECIPHERING THE PHYLOGENETIC RELATIONSHIPS OF EDF, PLANT, AND CYANOBACTERIAL ETHYLENE AND CYTOKININ RECEPTORS

**FIG 5:**
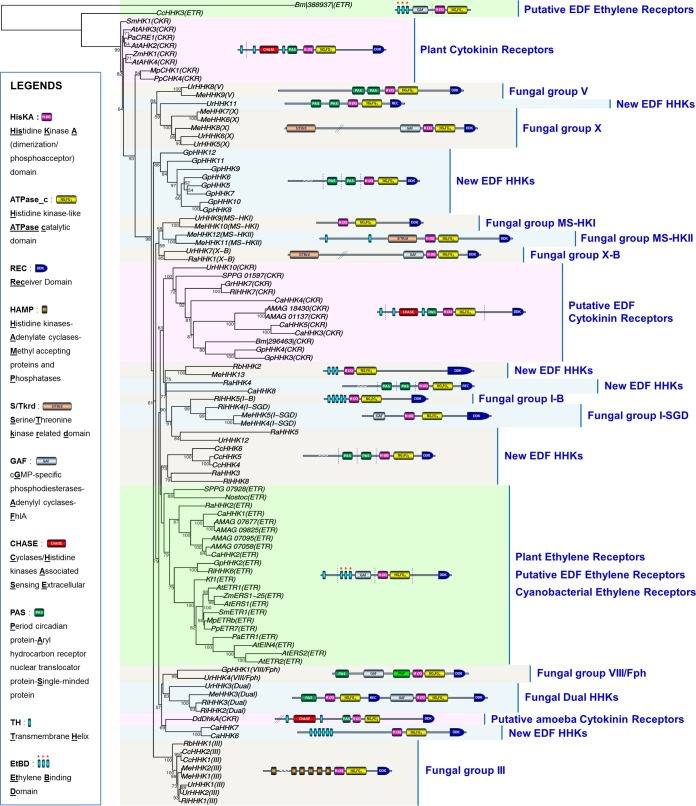
Phylogeny estimation of HHK predicted protein sequences. Methods used to carry out this phylogenetic analysis are provided in Text S1 in the supplemental material. Early diverging fungi predicted HHK sequences were categorized following sequence analysis of both HisKA signatures and N-terminal sensing domains according to previous classifications (4, 10). Abbreviations: Ri, *Rhizophagus* *irregularis* (*Glomeromycotina*); Me, *Mortierella* *elongata* (*Mortierellomycotina*); Ur, *Umbelopsis ramanniana* (*Mucoromycotina*); Cc, *Conidiobolus* *coronatus* (*Entomophthoromycotina*); Rb, *Ramicandelaber brevisporus* (*Kickxellomycotina*); Ca, *Catenaria anguillulae* (*Blastocladiomycota*); Gp, *Gonapodya* *prolifera* (*Chytridiomycota*); Ra, *Rozella allomycis* (*Cryptomycota*); AMAG, *Allomyces* *macrogynus* (*Blastocladiomycota*); Dd, *Dictyostelium discoideum* (Amoebae); SPPG, *Spizellomyces* *punctatus* (*Chytridiomycota*); Bm, *Basidiobolus* *meristoporus* (*Entomophthoromycotina*); Gr, *Gigaspora* *rosea* (*Glomeromycotina*); At, *Arabidopsis thaliana* (dicots); Zm, *Zea mays* (monocots); Pa, *Picea abies* (gymnosperms); Sm, *Selaginella moellendorffii* (lycophytes); Pp, *Physcomitrella patens* (bryophytes); Mp, *Marchantia polymorpha* (liverworts); Kf, *Klebsormidium flaccidum* (charophytes).

A cyanobacterial origin of plant ethylene receptors was previously suggested (27). Although it was recently proposed that cytokinin perception by plant HHKs through the CHASE domain might have emerged shortly before the conquest of land (23), the exact origin of these phytohormone receptors remains unclear. To gain insight into the phylogenetic relationships of EDF, plant, and cyanobacterial ethylene and cytokinin receptors, we generated a robust phylogenetic tree after multiple alignments of all the predicted sequences compiled in this analysis (Fig. 5). This revealed that cyanobacterial, plant, and EDF ethylene receptors tend to cluster, supporting a common origin (Fig. 5). Emergence of EDF cytokinin receptor homologs, as currently believed for plants (23), might have resulted from separate transfer and specialization of an ancestral, hitherto-unknown CHASE domain-containing prokaryotic HK, since plant and EDF cytokinin receptor homologs do not occur in the same cluster (Fig. 5).

## PHYTOHORMONE RECEPTOR HOMOLOGS MAY HAVE PLAYED AN ESSENTIAL ROLE IN FUNGAL LAND COLONIZATION AND FUNGUS-PLANT INTERACTION PROCESSES

We have described here for the first time fungal HHKs that share a high degree of similarity with plant ethylene and cytokinin receptors. Importantly, these homologs to phytohormone receptors were found in large numbers in several flagellated genera (e.g.,* Gonapodya, Catenaria*,* Allomyces*, and* Spizellomyces*), which are reported to colonize decaying plant material, where cytokinins and ethylene are omnipresent (25), and in a small number of some nonflagellated EDF they are known to behave as plant root symbionts or endophytes (e.g., *Rhizophagus* and *Umbelopsis*) (Fig. 3 and 4).

**FIG 6:**
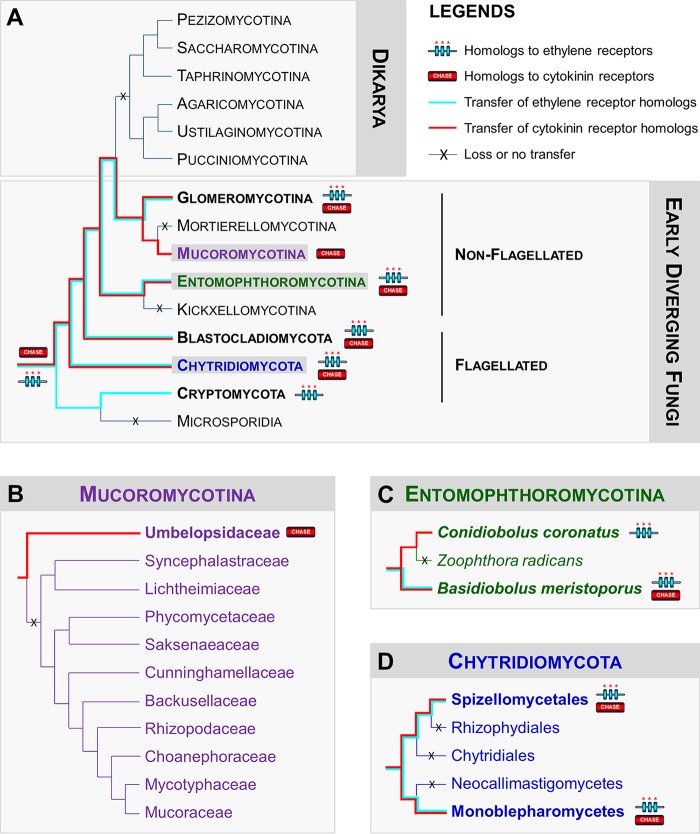
Distribution of homologs to ethylene and cytokinin receptors in the early diverging fungal lineages. (A) Phylogeny of fungi. Homologs to phytohormone receptors were found in lineages which included flagellated early diverging fungal species (from *Chytridiomycota*, *Blastocladiomycota*, and *Cryptomycota*) and some nonflagellated species, which have been reported to colonize decaying plant material or to behave as symbionts or endophytes of plant roots (from *Mucoromycotina*, *Glomeromycotina*, and *Entomophthoromycotina*) (B) The particular case of *Mucoromycotina*. Homologs to cytokinin receptors are exclusively present in basal species from the *Umbelopsidaceae* clade (facultative endophytes of plant roots). (C) The case of *Entomophthoromycotina*. Homologs to ethylene and cytokinin receptors are detected in the basal lineages (e.g., *Basidiobolus* and *Conidiobolus*) that are capable of diverse ecologies, including colonizing decaying plant materials and parasitizing insects. As the lifestyle progresses to a strict reliance on insect parasitism (e.g., *Zoophthora*), the presence of the homologs is lost. (D) The case of *Chytridiomycota*. Both homologs to ethylene and cytokinin receptors are detected in species that colonize decaying plant materials (e.g.,* Gonapodya* from *Monoblepharomycetes* and *Spizellomyces* from *Spizellomycetales*), but not in others (e.g., the amphibian pathogenic fungus *Batrachochytrium*, *Rhizophydiales*, the saprobe *Rhizoclosmatium globosum*, *Chytridiales*, or the mutualistic fungi of herbivore guts, *Piromyces* and *Neocallimastigomycetes*). The topologies resemble the current understanding of the relationships of the fungal groups according to information reported in reference (7–9 and 30).

Strong arguments suggest that interacting with fungi was one of the major processes that promoted land colonization by plants (28, 29). In addition, as previously mentioned, ethylene and cytokinins are prominent phytohormones that orchestrate interactions in several plant-fungus symbiotic systems (12, 13, 15, 16). On the basis of our observations, some hypotheses may be therefore put forward to explain the presence and distribution of these ethylene and cytokinin receptor homologs in the EDF lineages (Fig. 6A). Ancestral aquatic fungi may have harbored a pool of archetypal ethylene receptors and CHASE domain-containing HK genes that may have been transferred horizontally from cyanobacteria or green algae (23, 27). These gene families have undergone rapid expansion in some flagellated EDF lineages, including, for instance, *Chytridiomycota* (e.g., *Gonapodya* and *Spizellomyces*) and *Blastocladiomycota* (e.g., *Allomyces* and *Catenaria*) (Fig. 6A and D). The presence of these phytohormone receptor homologs may have initially participated to potentiate EDF-EDF (e.g., ethylene receptors in *Allomyces* and its parasite,* Rozella*), EDF-cyanobacteria, and EDF-green algae communication (most cyanobacteria and green algae harbor such phytohormone homologs) (23, 27). Later, these same signaling pathways could have been coopted for EDF-plant interactions and coevolution (28). The presence of a unique member of both ethylene and cytokinin receptors in *Glomeromycotina* (e.g.,* Rhizophagus*) (Fig. 6A), which currently represent the sole group of EDF that cannot be cultured without their plant partner, may reflect the optimization process that occurred in some EDF lineages to sense the host plant for symbiosis establishment. Once plants successfully colonized land, notably *Glomeromycotina* and the related *Mucoromycotina* (29), and developed more hospitable terrestrial habitats, such as soil, wood, and litter, ethylene and cytokinin receptor homologs were progressively lost in the genomes of fungi (absent in the Dikarya and most of the nonflagellated EDF lineages) that have shifted to other environmental niches. The striking truncated structures of ethylene receptor homologs found in *Conidiobolus* and *Basidiobolus* (*Entomophthoromycotina*) (Fig. 3A and 6C) and the intriguing presence of a unique cytokinin receptor homolog in *Umbelopsis* (*Mucoromycotina*) (Fig. 6B) and *Basidiobolus* (Fig. 6C) clearly illustrate this phenomenon of gene erosion, since these genera are considered basal within their respective lineages (9, 30). Moreover, we propose that EDF species that originally colonized plants and decaying vegetation lost their receptors at the same time they gained the ability to become pathogens of animals living in the same terrestrial ecological niche (e.g., *Catenaria* and *Conidiobolus*) (Fig. 6). Additional examples of plant host-to-animal host switches in the EDF will be necessary to confirm whether loss of these phytohormone receptor homologs is really linked to changes to non-plant-associated lifestyles.

## CONCLUDING REMARKS

The discovery of unprecedented homologs to plant hormone receptors in EDF suggests the participation of these sensing proteins in fungus-plant interaction processes, which may have helped these early diversifying fungal lineages to colonize land. Work is under way to functionally characterize these receptors and to decipher their physiological roles in EDF via complementary biochemical, genetic, and modeling approaches.
