# Supplementary material for: The Identification of Phytohormone Receptor Homologs in Early Diverging Fungi Suggests a Role for Plant Sensing in Land Colonization by Fungi
Source: mBio. 2017 Jan 31;8(1):e01739-16. doi: 10.1128/mBio.01739-16 (PMC5285503; doi:10.1128/mBio.01739-16)
Supplement: Text S1 [file mbo002173159s1.docx]

**Phylogenetic analysis (Figure 5)**

Methods: Because of a strong divergence between our sequences, we used a phylogeny approach in order to cluster them. A total of 98 protein sequences were first aligned using COBALT (Papadopoulos *et al*., 2007) command line application with fast minimum evolution guide tree (Desper and Gascuel, 2002) and the regular alphabet. Conserved domains were searched in the NCBI CDD database v0.75. Non-conserved positions were removed with GBlocks 0.91b (Castresana *et al*., 2000) using the following parameters: at least 50 sequences for a conserved Position, at least 50 sequences for a flank position, no more than 75 contiguous non conserved positions, a minimal block length of five and considering all gap positions. This curation resulted in 672 new positions (10% of the original 6143 positions) which we believe was sufficient to remove non-homologous positions. A Blastp analysis on the resulting alignment indicated that HisKA, HATPase and REC domains were the most recurrently found in the curated sequences. This was expected as these domains were the best conserved (Figure 1). The curated alignment was next processed into R (R development core team, 2013) for maximum likelihood phylogeny estimation with the 'phangorn' package (Schliep, 2011). A starting tree was constructed with neighbor-joining algorithm. This tree was used to build a maximum-likelihood phylogeny. For that purpose, we ran the 'modelTest' function in R to find the best adapted model including testing of intersite variation and invariants. We found that the amino-acid evolution model proposed by Lê and Gascuel (2008) with integrating intersite variation was the most adapted according to the Bayesian Information Criterion in comparison with other classical evolution models such as those proposed by Dayhoff et al (1978), Jones et al (1992) or Whelan & Goldman (2001). Using the model of Lê and Gascuel, topology and edge lengths from the minimum evolution tree were optimized following a stochastic approach in a maximum-likelihood bootstrapping procedure over 1000 permutations with the 'bootstrap.pml' function in R (Schliep, 2011). The resulting optimized tree displays the most probable clustering of HKs based on conserved positions. This was useful to obtain clusters of divergent sequences in order to infer a potential biological function. Edges without values indicate a bootstrap value <500.

**Castresana J. 2000.** Selection of conserved blocks from multiple alignments for their use in phylogenetic analysis. Mol Biol Evol 17: 540-552.

**Dayhoff MO, Schwartz RM. 1978.** A model of evolutionary change in proteins. in In Atlas of protein sequence and structure.

**Desper R, Gascuel O. 2002.** Fast and accurate phylogeny reconstruction algorithms based on the minimum-evolution principle. J Comp Biol 9: 687-705.

**Jones DT, Taylor WR, Thornton JM.** **1992.** The rapid generation of mutation data matrices from protein sequences. Comput Appl Biosci CABIOS 8:275–282.

**Le SQ, Gascuel O. 2008.** An improved general amino acid replacement matrix. Mol Biol Evol 25:1307–1320.

**Papadopoulos JS, Agarwala R. 2007.** COBALT: constraint-based alignment tool for multiple protein sequences. Bioinformatics 23:1073-1079.

**R Development Core Team. 2013.** R: A language and environment for statistical computing. R Foundation for Statistical Computing, Vienna, Austria. URL <http://www.R-project.org/>.

**Schliep KP. 2011.** phangorn: phylogenetic analysis in R. Bioinformatics 27:592-593.

**Whelan S, Goldman N. 2001.** A general empirical model of protein evolution derived from multiple protein families using a maximum-likelihood approach. Mol Biol Evol 18:691–699.

***Compilation of sequences included in the study***

>[RiHHK1(III)](http://genome.jgi.doe.gov/cgi-bin/dispGeneModel?db=Gloin1&tid=93395) Gloin1|93283|

MTDVLGYVTDVLQNINDGNVEYCKTVRYQGQRNEQIEQFDSVLRKLLEKHIYLEEEVARYQSLLNENDSVVIIKDSSLPATNNEDCLQVKQSNETAILAPPSDKCQTNQINSNHINNQIHDSNSDSSPNTPPSLEHGDRVCLECLDQCTNVALAVTSGDFKKRVICPVASGPMLTLRNAINTMVDKIDRVSIELIHVAREVGEEGKLGVQSKSENLEGDFRDMMVHLNMMASNHSKQVRDIAEVCTAVAHGDLSKKITVEVKGETLVLKNTINTMVDQLNSFASEVTRVAHEVGTEGKLGVQAQVQGVGGTWKLLTDNVNTMAANLTAQVRDIADVSKSVARGDLSKKITVDVKGEILDLKNTINTMVDQLQTFATEVTRVSLEVGTEGKLGGQANIKDVGGIWKDLTDNVNLMASNLTNQVRDIATVCKAVACGDLSRKVTVPVQGEILELKVTINTMVEQLRMFAAEVTRVAREVGTEGMLGGQAEVQGVDGTWKILTDNVNTMAANLTAQVRDIANVSKAVARGDLSKKITVDVKGEILDLKNTINTMVDQLQTFAIEVTRVSLEVGTEGKLGGQAVVKDVGGTWKELTDNVNIMAANLTTQVRSIAEVTTAVAKGDLSKKIIVDVKGEILDLKNTVNSMVEQLRTFAAEVTRVAREVGTEGKLGGQAVVKDVGGTWKELTDNVNTMAANLTAQVRDIANVSKAVARGDLSKKITVDVKGEIWDLKNTINTMVDQLQTFATEVTRVSLEVGTEGKLGGQAVVKDVEGTWKVLTDNVNIMAANLTTQVRSIAEVTTAVACGDLSKKISVDVKGEILELKDTVNSMVDQLRMFAAEVTRVAREVGTEGILGVQAHVKDVGGTWKELTDNVNTMASNLTAQVRDIANVCKSVACGDLSKKITVNVKGEILDLKNTINTMVDQLSTFAAEVTRVAREVGTEGKLGVQAQVKDVKGTWKEITSNVNTMASNLTSQVRAFAQISAAATDGDFTQYITVDASGEMDSLKTKINQMVYNLRESIQKNTAAREAAELANRSKSEFLANMSHEIRTPMNGIIGMTTLTLETELTRQQRENLMIVSTLANSLLTIIDDILDISKIEAGRMTIEQIPFSLRSAVFGVLKTLAVKANQKKLDLIYDVDNGIPDQLIGDPLRLRQVITNLIGNAIKFTTEGEVVLSVHTQLIEDDRVILEFCVSDTGIGIQEDKIDMIFDTFCQADGSTTRKYGGTGLGLSISKRLVSLMGGDLWVKSVYGHGSEFYFTVKVTLGTMSRETIEQKMNPWHGRHVLFIDTMHDKTGVVSIIEELSLRPKIACSVEEAALITASRKSDTPLFDTVIVDDLSIVERLREITHLRYIPIVLLAPEIPNLNMKNCIDLGITSYSSTPSNLPDLMNALLPALESHAAVPSDFARQQPLDILMAEDNIVNQKLAVKILEKFGHRVEIVSNGQLAVEAFKAKRYDLILMDVQMPIMGGFEATQKIREFEQETGGHVPIIALTAHAMIGDREKCLFAGMDEYVTKPLRMNDLIATINKFPVKNPIEEISIPDGRNDTISSISSKI

>[RiHHK2(Dual)](http://genome.jgi.doe.gov/cgi-bin/dispGeneModel?db=Gloin1&tid=93941) Gloin1|93829|

MDKALTFMSFAGTENISNFSEFPNQYDQIPAADLVESFDWSSTSLGPMDEWENTLKSTVKLCMHSVFPIAIYYGPDLVNIYNQMWIPILKMKHPQALGQPFKEVWAEIYDDLEPLFNEVLSTGKGKFEYDRFFFLLRDGYLEETYFSFTFSPIFKDDGSVGGIFNASQETTQRVLSNRRVKTLSELGKRTPGAKSLENACHLVTRTLHEKNKDIPYALIYLIENSTVKSTLQPREAHLIASTFDEDLDYVKCEDGVDEISFVPNKSRRVLPDYLLETKEIIDLMVVPDEINELINDDSKFELSARTGENFVASKDFLTSWPIHYVAMTGSHITITLKNGSSAVLYPVTTSSGGKSVLTAVIILGINPHRALDKEYMEFLQLIVGQVCISLTHGKSREEERKQAEILADLNRQKIMFFQNISHELRTPITLMLSPLDEVISECSNDSPMHPHLNMIQRNTRRLLKLVNTLLQFSRIEAGRMEAIYYEVNIGLLTLELASNFESMAKSLNLEYRIEIPENFDKMLEKKVFVDLDMYEKIIFNLCSNAFKHTWTGNVTVKLSVKWVENKEVIVLEVSDTGVGIPKEEIPKLFQRFYRIESRQSRSHEGTGIGLALIHELITRHGGGIKCDSELAKGTTFTIWIPTGHEHLPPGRLLFQKDMKEGKGYLGQENKLFDNKQLYLEEGLQWIQNNEPDSNEDAESPIDKMNVDEVEEIDHKGFFTGDLERSYPIEDDPIPLSGTKHVVLLADDNTDMRNYLSGLLKKEFIVHCACDGREALKKLKKLKNPPDLILSDIMMPNMNGFELLKSIRSDISTQLIPVILLSAKAGEEASIEGLDKGADDYLTKPFSARELIARVRVNIKLSYLRRQLFLQQRQQAETKQLLFSISNKIHSGFNLQKTLSTAAEEIHRTLSADRLFITANDQFENGDGIIEAFSAKDKSEKNIKGQCFKFNSEQIRLHSDPVIVEKLIKELEERRLLDEENDIHNIIDNANKAIAIMTNEQNQFFDKTLKDQKDITNEIGISTSTSSELSKNSKNQSESSNLSDKEDLDTAEIANFYSINVQKYVSLLAVAIKVNQSTWGWLIVHRPPNSVWLDSEKEFLQQISNQISLAITHAKLLEDKLRREAQIEAARAANEAKSQILANTSHELRTPLGAIIGVLSAFEDTALTDDQKDMIQIMTRASDVVLAVVNDILDAAKLEAQKIKLVNRTFDLFDLVEKTIEIFGEKAGNKQIELILDCEPNSLPKDVRSDPERLQQILMNLLSNSIKFTENGKIVLKISMIPYEETEMTSVVQGQEAGKKAKLYVELCDTGIGIDPAFIKDIFKSFSQGDASMTRRQDGTGLGLSICKHLVDINGGEIDVVSELKKGSRFWFTWNVDIPATSISLDSLNDSLNEQTGLVLPSAEQYKRVLIIDSVEAARNSLVKLFKDSVDKVDAFNSCEEGVNAVSQMVEKHNEPPYDVVFFNVYKENAELVKNSALQLRSICGQDLSIALLVFWSANGRALGKDLIYQIGGHTAALCKPIMHKRLLDCLRNNDIFKQSDITSPSKHDRGEYSYVKSLADIRVEKYYHQNRSLDTSITNRNENDSMIIGNNQENDQSTDPMVIDEDSTVKKEIKSQVSDTTSSSSTQTGENMVIGVKRNVTDNNPRAQKSRSRKITKSKCILCVEDNPINLRVIQHQLAKLGYPTLSATNGQEAVNVIEAEIANSTPNDESPRISLILMDCAMPEMSGFDASKAIRLFQSPLSKIPIIALTASAVQGTRDRCLESGMNDYLTKPLKIGQLKEMLEKWLGEE

>[RiHHK3(Dual)](http://genome.jgi.doe.gov/cgi-bin/dispGeneModel?db=Gloin1&tid=90992) Gloin1|90880|

MSVLGKESPYADEAPVADLVHSFDWSTTPLGDRSTWPPSLKTIVDLCLHSVFPIAIFYGPELRMIYNQMYRPILKMKHPQAMGRSFKEVWSETYDVMGPIFEEVNLTGIGSFQDDMMLLLHRDGYAEECYFSFTLSPVFKEDGTITGVFNAVQETTQRVLAVRRLKTLEDLGNRTPGAKSAENACHLVSLALQDNDDDIPFAMIYLLEDDNHQKNIKKVKLIATTYDQTLMTIKGDDEIEELAFVNGISKRILPDFLSEEFTNEFIYDGNIIMDKNNSNNNDISHWHLQEVITRNSHVIVTLKDDSKAILLPVLSSFAGETNTTAIMVCGLNPRKALDRDYMEFLRLAVSHVSTSLTQGRLREGERKHAQLLADLNKQKINFFQNISHELRTPLTLMLSPLEDAIACCPNNSDVLLHLQMVRRNSRRLLKLVNTLLQFSRIEVDSIKAQFHETEIAKLTAELASSFESMAKSFELKYVIDVPKELKLSRRIFIDQNMYEKILFNLCSNAFKHTWTGSVTVRLYSENEDDKEFIVLEVIDTGVGIPNDHIPNLFNRFYRVESRQSRSYEGTGIGLALVKELVNRHGGDISVVSVVDKGSIFKVRLPTGWEHLPPDQVYFDDEYNTKEHVVHGEKLYSNCDLFLEESAQWIQKNNIEDSDYKDMDLDDNLTTEGHTIPSSLEDNPLTFGENFTVLIVDDNTDMRDYIFGVLKKDFDVCCACDGLDALRTLKKLDKPPDLILSDVMMPNMNGYDLLKSLKNNKSTRLIPVILLSAKVGEEASLEGLEHGADDYLIKPFSAKELIARIRVNIKLSCLHRQLFLQQKRHLETKRLLFSISRSGLNIQETLSTAVQEIHHVLPCDRFFIVAVENEDSRIMAFSSTDQSEPNLQGKLVYFPIKENNQTENLNQNSLEVVNETLEVVIFPNYYLLAVQNLVSLIALPIKIDSKTWGWVVANRPPNNTWLDSEKSFLQQMSNQIGLAINHSILLEEKLKKEAQMEAMEAANEAKSQILANTSHELRTPLGAIIGILSAFEDSQLSEDQKDMVQIMTRASDVVLAVVNDILDAAKLEANKVALLNSTFDLFSLMEKTIELFGEKAGAKNIELILCCEPTSLPKFVKSDPERLQQVLMNLLSNSIKFTDKGEVCLKVTMKNDNESSKNTTAKKSTLFVEIIDTGIGIDPRFMKVIWESFSQGDASMTRRQDGTGLGLPICKQLVKINGGEMGVQSELGKGSRFWFTWLVESSLSWSPIPITSISQTPPGLNLPTTIRLKRVVVIDPVETARNALVKLIGSSVERIDSFDSFSEGVSAAKVWRETHNGPLYDIAFFNVNEDNVEEVKKASEELRYTCGKDQLCITLMVYWSASGRAIGQKLIKDIGGPIVTLCKPIMQKRLLDCLHNSKIFRSSSSTPNHHRRNSVKPLADIRVEKYYNDNRHLSSLPVEKELTIIRQSPKDNNEKLSQASSRDKLKRTASFSSEPTKRTALKRMEPSGEDNNQIDKNRASKSLRSRPISKSKRILCVEDNPINLKVIQHQLKKLGYPSLSATNGQEAVHLIQAEYSGESSNLSLSLAKEIPTKISLILMDCAMPIMSGFDASKAIRTMSNIPIIALTASAIPETRERCFESGMNDYLTKPLKIGQLKDKLIQWLGDDN

>[RiHHK4](http://genome.jgi.doe.gov/cgi-bin/dispGeneModel?db=Gloin1&id=138626)(I-SGD) Gloin1|138626|

MATPKKPNSLRRSKTVDCRRSKIVCELNSTAEADAITSFIKGLEDDCEQSKCFVDYNIEVDGNYESIYDDDDLDDDLDDNHYLPPPMPPNEVERRRALWRFQILNTSNDVNFTRIVALSKEHFKTSISMISLLNTTHQWFKAEDGMGCSGTTREISFCGHAILQENGEPFVVLDATKDWRFCNNPLVTDAPFIRFYAGAPLRTDDGYNIGTICVIDREPRDSFSKKDRENLKEYARVVMRELELWTDTLRLRVRNKMQESIAEFSKFCLEIQLANNNSNNDENKKKSTICDPIMKQCFNMAVKLMRDTLNVDSVYLLEMPCLYSRPLSITSRNSNNFFLTGPPPDVPSSHLRSLASVGEIELSTEALKASIITSYFTYLMQTQSQGCIYQNSLPPLPTLFPDDVHSGIVVPIYDDTQNAFGFLIAMTKDPQRQFEDEERVYLSNFGVNVVSEVLKRRVIVADRAKGAFISSISHELRTPLHGILASCELMEESKLNESQAELVKTIQGCGTSLISIINSVLDFAKLESEKQDYVDDPINQKKLSEKDNNNKLMNRNNNNNKRKKEQKERIDLVKLLEEVSEACFVGQQMVTAIYNDNNKITNSINKNNDEENLLMENNNNNNNNNNTAITQARKKRVYDLLHPNQSHHQQADDVLLMIDVEPRDAGWWVMAEDGALKQLLMNIIGNSMKFTKKGYVLISLASLSYSSLSDQYKKHAGVQDTTTTTTDTSSSSSSSSNKIHALITITDTGCGISPSFLSTNMFQPFSQENSLQVGTGLGLSIVKLLVEKMGGKLDVESEVGVGTRFRIWLDLDQANEEIKMNYNKNDDLGDFSNTFDSFNNENDNLIKDFEEKQRKIILEKVKQKRIVVKCVDGKLKEVVERYFKGWLKVKELICEDKENVTDSTLDGDIIFIIDDIGQLKKIISKVDTKQAPIVFTTTLAKHGKIADFVEKLQKEIEETQLIKRKRRKVVILTRPCGPRKLEKAIVSVLSSQQDDEKLLEEENSYFPKLIMTPPISEDDNSKDYISYNSSEKSSSISPNINDPLNKSDRDSSLLSVAPPLRPCFNRSTTLPNLHLPSGNKEPKFLPIPKSTFLLTPSTSPCATPTAMSTSLPNMSESLNINPPPLNELKKSKSVNSLSCNLGPRVLVVEDNAVNRMILATFLKKRGIRFDEAENGAIGVEKFKKALEDDDGEFPNRKGFDIVLMDIQMPVMNGNVATAEIRKIEEEFNNPKFSLLSTARTTTNTTLTKSPAASPTITNFEFPPSSLFNKTTSSSPVSESNNHLKPFTQKNRFLQRRVSNPDQFSFQNNSFSITSLLLKPKISIKIPTKSTTKSKTSPLPSPSPIPSSPSFPIIENNTINTITKRSRSLIFALTGLASEEDKDIAFESGVDGFLTKPVSLKMLEKVLKKWSEKNEQSSENSSVGSSNSSSNSSSGGST

>[RiHHK5](http://genome.jgi.doe.gov/cgi-bin/dispGeneModel?db=Gloin1&id=327799)(I-B) Gloin1|327799|

MLPSKKLNLSNYHTIFDSLIPRDILSQDYERDYLARRNLLQARAIITVIVSAMLTLIIQLVVDLYLSYKHVKLLHIAWPFSFIGTLICAVILYFIPSGRITISQAAKVGIYFFLSISAYTSCIGTQIQTLSHANFFVVPPFGYIFVSKEFGKQTTIVVCIMHVAAFFRSLGRQINPISLSSSGWVVCDVYILCFIYFLFVAYEQEFQSNQERTQKSLDEARSAATAKSMFISNVSHELRTPLHGILATVEILAKTQLNESQRTLLSAIESCGTNLISVVNQVLTYARIEHNKLELEHNVFDIYTVMQEIGDGLAPIPESKNLDFFVCVEANPLQRFLVGDVGVLRQIILNLLGNAIKFTDKGRIELVTIEITGESKKDGQEQLKRQVITSKEFETIKNSSASVQINSPGQLSENQKDNIQKSPKAQFRINVNDTGRGIAPDFISHMYQPFSQEDSSLKRKFEGTGLGLSIVKGLLDMMHSQLEVDSKLGQGSKFSFKLELPISDRLDDMNASPLPFDHKYLKLPPSEQEMLIKRLRSLSYVILNVEEFLLSQKIAHYFDQWEFSYRLLSIDKLKFECTKQHADVVILNDNINDLELFLNEFIQYYRKPKNMGIEEESIDSVMKKRQHVLFFSTIENYQQAETLLMKYKPQSVEIITKPAGPVKILTAIIKTIESSFSKYDGIMKTQEEKIRSIREEYLDFIKSSNNHKISTMDIGPSGELTISPSRLTPPAMIERIISEVSGEDKKTFFDQEQLKEESSMQLKTKGVKNKYKEETEKKLQKSPKDISFLIVEDNKINEMILTTMLKQSGYYNYDIANNGLEAVENFTKKSYDIIFMDLQMPICDGIVATKEIRKIERGEESYLLQSHTSSPKSQSPSDTSAEGTCKIGRRRRKKAIIVAMTGLASDEDSQAAEAAGCNEFLTKPVSIKSLNARMESWTKEALKVDEEAISSEEKND

>[RiHHK6](http://genome.jgi.doe.gov/cgi-bin/dispGeneModel?db=Gloin1&id=346986)(ETR) Gloin1|346986|

MARFIMLINCLFLMNLLCYFMFPVSAIPTPQDTQKNDKIIPTTNTDISSPSKIPSNRLLFTGGHWFNEPDMITFFVRMDESVNIDTIPSTYLIIGNSERILMSSHLNNVSGIGLIRTVSFPRDGHCREYHFETLQFGNVNRWPKNNDLLTVEGDRCLMPINEGLAWFICIVDLSIAFAYYTIPFQLMYFMRKAPKLPFPLVFGLFCAFIILCGTTHIVASWMAWYQTHVLSAVIKVICAIVSLFTAGALTVIIPKALDLPMKAVQYKDEIAVRLITESNLRDENQTMADFRRITHAIRATLSKEIIYHNTAFQLCGILEADRCQIFTSKSIKAKFEGWRCAQDYVDGSESPLNVNSQLNPECSIIYSTIEKRAATILGLDDKEFANLYLEGDYQNINKIILVPIAISENENGIIVIQNWDQVEYLTQEKWENTRTFLTDLSEQISIALSQASLIEQDKIRIHQLAEQNEALIQARKEASAIQAHREFLAVMSHEMRTPLYAIFALTSMLLEMPYLSHQEMSEMRDMLEIIKKSGDMLITIINNVLDFSKYEEEQLHLERVPFCVHEAVETSLDIVSLQGQETSRPRINYVIDKNVPYNVIGDMTRFRQIIVNLLSNACKFTNADGDVMITVCSEFVEKNNTKKIRIKAEVTDTGIGISKEVLPKLFEKFSQADASITRKYGGTGLGLAIVRKLVKLMNGDITVEQNTSASSGTKMIFYVDLDVDPNSPTCPSLSPFLRQKSLSILEKHENNKIGLKQILNRCGLQRSPKFFTSLKQIIEDVDFFKGHIDAIIIDFQLLVENNEIEFLKNGIKDKLLKIPILILVNPSLQRSIKSFKIEGDNVIFSSLPVKFKPMLSFLEWNLREEDENSTLVMTESIGTNNEGSIMHYSCNGSYDSDFNSEIMSQNNDTQTEEELPLIDLSSSPDEYYSDITSKQRRISLSSNERPGYNRKNSASTTNLPTLSLVNKSSESENSPKIGSHAIRRTATLPVSFDMSNFETISNDNTSVIPSECLKFGNVETDIPKLKVLVVEDNVINQMVTSRILEKLNQKCEIAGSGKTAISKCEEKDYDVIFMDIMMADMDGFQTTEQIRSTSQDLRRPWIIALTANALWYDRFRCIESGMNDFVSKPAKKEDIREALMRYLTRNSATATKNNNQQPHQSHQSRPSLSTSTSSTNVNGGYGSKI

>[RiHHK7](http://genome.jgi.doe.gov/cgi-bin/dispGeneModel?db=Gloin1&id=22775)(CKR) Gloin1|22775|

MPHSSWFFSSKDKNSERDRLHSFRQRFMFNIFTRLFPQMTDKNTSNDNLRQDKEEKEEKEKEIRNSNITTATTITADSSITNEKNALIQVVEGESNKEANISSGLGNLSTTAYDFCNQSRRPSDISSEIIRPTEHVVNLSTMEPHDTVDISTLPSNNYTRQYRNPYFNTGSNSPIPQQPKGFFTRIKPTKETLAIIFLPLIIALIGVTIAIVVLLFVFDQEVKNYRDSFDYICMERISAVVSTFNSGLNTGRDFTGFFTVFASVYPNLMDDADEIVSNYGNLSAINNMNIYTMNFAPRIPFANRSDYEVKHGQIKQLNPQNQLVIRADAAEYFPVLYSIPWRYNTPIILNYDVSSEPERLAAITKARKTRNITITPRIALAYDHTKGGISVYFPFYMNNFDDISGLVIGIYEIDDTIRSVAKNFNDKPGIGFDIIDTESNTPIYSKNDSIIWHSANFNKQANYLIGDRNWQFTCYSSKEAYNRSVSMYGPIVYFVLILSFFSIIALFTSNYFRKLFKARYKFTEQAVKLGRTQSLLKAITADSKAVLEAIADPLFALNAKGEIVGANKHALTLTGYSPEEIKVANKMHINTLLIPVAETPTEERNNNPLNGDYQLIEVPVRPGMRDVLAKKKDGTLFEAEANFSQPVVEKNYFTQVVMFRDVSFKKENERAVMDAKRDADLANQSKTEFLFFLCHEIRNPIHAILGFAEMLKNSLKEKEQFEELEYIISAGKFLSFIVNDVLDLTHLTNPNPYEIDLKCDGFNIYTLVDNIAKIQSIQADHKQIQVKTIISGELPRNLYGDERRLEQILMKLLARSIEVAPSNGIVELIIEQKIVHHSKGILLRFSVKDQSKGLSSDEEINELFKPYAKTNSSIGSRFHAQGLSMALVQAIVRVMGGRLVVEKVDRSSSGSSKRKSNVNKKNNNHVWFEIWLLTEESKDRGRLLRESYDSLVISKNGSAGRNSFKISSAPGSIDGKMMDDIVINSDNEDAISHLRNRKSMPAKPKRDSKSYMNTIRKKRRSTTVPATSAVMNNGGEDPIDDEAGSIKNVAEQSKSAAANFFRKGSMMLASGLARAGSTIEKPSSSPLSTSFSNITANDDSIHRSAGKNIASSVQIESQESTLPLPIISYYSGGNTSISPGSPVPPVPPLTQDHNLLSSSNTNKAASIKSKNSSNISLIITNVNTSPNVSTSPHDTPASISPTSTGSLNQHSLHITLPNNNLPPLTSYDAHISPLTLTADAKNIGSITNNSNNDNDNKPSSTLDNQADTQVNAVANISNISAATEKPKKLRILLVEDNLVCQRVTYKMLNRNNYSVDIANHGKEAVDMVEAVQQQQQQQYACILMDIITPVMNGYEATQILRDRGVKIPILALTANSFESDVKKAKEVGMDDFLTKPIKEVELITAMKTQIEKFENTDSSQSQQDDFMAEHKDQYLTINS

>[RiHHK8](http://genome.jgi.doe.gov/cgi-bin/dispGeneModel?db=Gloin1&id=94431) Gloin1|94431|

MDHANSFFSTVLENSSPTLVTDISGRILYVNRPALCLLERCRPFKRPNANVLNNDDPEPSSFINSHYSSIIEPLDAYEATSLRNHFMNNVQILSKRSHVCRTIKCRTFSSKRPIMLEFSTRTCTYPEFFKDNSGEKNFSSPSSPPKNDDMYLRNSSVISLNTSSSIDGKEGIESEGGGGVEEEDDDDENEQFFDAKEYSEEDILENNNILIQQSVNNNNFNVPMNHEKNSYHRTNKMELLNPKTQTITDSQLLTVVGSTEATNNLILISNIRDVTDSKFQEFVLNESDHRMSITYDVFGIITEISRSCKSILGYDPDELLGIDGYNFIHPDDSTKVRAFHAKRAVENYGTTLQRQSFRHLKKDGSYCLLEIWSQGIQPDGSWSFVSFNITDASENLIDKRNMCDQTFIRKLLPKNVEGETNNNASKNFDSYIFHYMYPSIKLRNPLNGIIGFTSLILDTELSEQQRDFAEAISSISSYMCNIINQSLDLCQTQSNRTTSSDIGPLRLREIVEFGFLTVTVLARTKGTELKLQDHLTPLQMSGQKYQSINGTTSQVKNEKKWYGDEDIVRKILLNYLTNAIKHTGSGVVTVRLEVDSDKLQDGKEGFMAKCSVEDTGPGIPQKDLKRLFLPYSKLVSSFGELKTLKEGNNGTKSPNHQQSHRSSGLGLSICKELADKMGGKVGVESVMGKGSTFWFTFPVYTSKPENMEDESDSSSSVREVRDNGKSIKNVFLTKSYSNSVFENGRDGVTTSSSTSAPSSHSNSNHTETNSKGDLNSENHRKHRKHRKPAKVLMVEDNETNQRIALHYLEKMGQKVTLAANGIEALEKMKNETFDILFVDLQMPYMDGYTLTRRIREAEREAEREAEQEACRCDKQDCESLLFSVSESSTPPPTPPGSSSALTNGHQSVIPLVSLIQSSQSISPLHINTQPPIISSTSSSLSSLSSSNYSYSSSSSSSESSSSSYSQSLLPSPTTTSGSTKNNVTIKNSHQSVPSSPPISPPSSSHKRLKHVPIVACTANVLDEEKGRCKQHGMDEELVESDDESLPGKDEKNNVENRDYNLPINQTNQINQYEKNLSIAPI

>[MeHHK1(III)](http://genome.jgi.doe.gov/cgi-bin/dispGeneModel?db=Morel2&id=1347040) Morel2|1347040|

MATTATAEEVYLDQVATIIDALLTGNHSDIPPTLLSLQQPTDSSNTPPQQPEQKQQQSQQSQQSPSISKVDLALSSLVQKILLIEADLARLRLEQKANATITTTEKVENHIQQRAPLILTPPVDILSPPISTSNRWATSAEGDAASTTWDFMNDNSTSYPSPYSAHPSSSIDLDGPSTIPSIHSYSDAASLSGSNSSQNSSSDSSTDAMPLPAPLPPPPPTPLTSSSSLPTTSSLNIPHSHHHQHRSLSPLSSSYESVGGGMSPYLGFHPGELSNSSRPSSPDSFNSALSSFPIDLLPELAVSPATPAASVLTPGLPTQGPGLPSSEPSALVLSALSFSGADLPSEEVPCAACIHHCALVAAAVVQGDLSLRITCDRPACNQSFLATSINAMVAKLSNFTEEVIRVAAQGVEGKLGVQANMEDEHGIWKEFVSHLNTMTVGHSEQVRDIASVCTAVAHGDLSQKITVAVKGETLVLKNTINTMVDQLRSFSSEVTRVAHEVGTEGKLGGQALVGGVDGTWKELTDNVNTMASNLTTQVRSIAEVTTAVACGDLSKTIDVQAQGEISELKLTVNSMVEQLRTFAAEVTRVAREVGTEGKLGGQAEVKDVGGTWRELTENVNTMAANLTTQVRDIADVSKAVAKGDLSKKITVEVKGEMMDLKHTINTMVDQLQEFATEVSRVSLEVGTEGKLGGQAHVKDVSGTWKELTDNVNLMASNLTGQVRDIADVCKAVACGDLTKKVSVPVQGEILELKETMNTMVDQLRTFAAEVTRVAREVGTEGKLGGQAHVKDVSGTWKELTDNVNLMASNLTTQVRSIAEVTTAVACGDLSKKIDVQAQGEISELKKTVNSMVEQLRTFAAEVTRVSLEVGTEGKLGGQATVKDVSGTWKELTDNVNTMASNLTTQVRSIAEVTTAVACGDLSKKIDVQAQGEISELKHTINSMVEQLRTFAAEVTRVAREVGTEGILGGQAEVKGVDGTWKELTDNVNTMASNLTTQVRSIAEVTTAVACGDLSKTIDVQAQGEISELKKTVNSMVEQLRTFAAEVTRVAREVGTEGKLGGQAQGEISELKKTVNSMVEQLRTFATEVSRVSLEVGTEGKLGGQAVVKDVSGTWKELTDNVNTMASNLTTQVRSIAEVTRVAREVGTGGKLGGQAIVKDVSGTWKELTDNVNTMASNLTTQVRSIAEVTTAVACGDLSKKIDVQAQGEILGLKNTINSMVDQLSTFAAEVTRVAREVGTEGKLGGQAEVEDVSGTWKELTDNVNTMASNLTTQVRSIAEVTTAVACGDLSKKIDVQAQGEILDLKNTINIMVDQLSTFSAEVTRVAREVGTEGKLGGQAEVEDVGGTWKELTDNVNTMASNLTTQVRSIAEVTTAVACGDLSKTIDVQAQGEISELKLTVNSMVEQLRTFAAEVTRVAREVGTEGKLGGQAVVKDVSGTWKELTDNVNTMASNLTTQVRSIAEVTTAVACGDLSKTIDVQAQGEILDLKNTINSMVDQLSTFAAEVTRVAREVGTEGKLGGQAEVEEVDGTWKELTDNVNTMAANLTSQVRDIAGVSKAVARGDLTQKISVNAKGEILDLKNTINIMVDQLSTFSAEVTRVAREVGTEGKLGGQAEVEDVSGTWKELTDNVNTMASNLTTQVRSIAEVTTAVACGDLSKKIDVQAQGEISELKKTVNSMVEQLRTFAAEVSRVSLEVGTEGKLGGQAVVKDVSGTWKELTDNVNTMASNLTTQVRSIAEVGTEGKLGGQAVVKDVSGTWKELTDNVNTMASNLTTQVRSIAEVTTAVACGDLSKTIEVQAQGEISELKKTVNSMVEQLRTFAAEVTRVAREVGTEGILGGQAEVKGVDGTWKELTDNVNTMASNLTTQVRSIAEVTTAVACGDLSKKIDVQAQGEISELKLTVNSMVEQLRTFAAEVTRVSLEVGTEGRLGGQAVVKDVSGTWKELTDNVNIMASNLTTQVRSIAEVTTAVACGDLSKKIDVQAQGEISELKHTINSMVEQLRTFAAEVTRVAREVGTEGILGGQAEVKGVDGTWKELTDNVNTMASNLTTQVRSIAEVTTAVACGDLSKKIDVQAQGEISELKLTVNSMVDQLSMFANEVTRVAREVGTEGKLGVQAQVNDVRGTWKEITSNVNTMASNLTAQVRAFATISTAATDGDFTQFITVEASGEMDSLKTKINQMVYNLRESIQRNTAAREAAELANRSKSEFLANMSHEIRTPMNGIIGMTSLTLETELTRQQRENLVIVSNLAHSLLTIIDDILDISKIEAGKMTIEQAPFSLRAQAFGVLKTLAVKAHQKKLDLIYNVHNDFPDQLVGDPLRLRQVITNLIGNAIKFTTEGSVVLDCVCKNKTDVGVELQFCVSDTGIGIQSDKIEVVFDTFCQADGSTTRKYGGTGLGLSISKRLVTLMGGDLWVNSTFGKGSQFFFTVRFNTGTMSVDQINLKTKPYTGRHILYMGTMRDEAISNSVMRTLDELRFKATHVSSLEEAAALVSMVPGERSSSSSSLTMSSEKSVFDVVIVDNVKDIRKIKEIGMLRFLPIVLLSMTTPYISMKVCQDLGIASYFNPPVHLPDLMNALLPAFESASALPSDAEHAIPLHILLAEDNVVNQKLAVRILEKFGHKVTIVANGKTAVEFFSSMHFDLILMDVQMPIMGGFEATQEIRRLEMLRRAAGGSAINGNNDHLPIIALTAHAMIGDREKCLAAGMDEYITKPLRVNELIATINKFPPKNCPDLAQNDHFYEYPNLRSNYSPSGSKTPIMFKSIDK

>[MeHHK2(III)](http://genome.jgi.doe.gov/cgi-bin/dispGeneModel?db=Morel2&id=1278143) Morel2|1278143| MVDQLSTFANEVTRVAREVGTEGKLGVQAQVRDVRGTWKEITSNVNTMASNLTAQVRAFASISTAATDGDFTQFITVEASGEMDSLKTKINQMVYNLRESIQKNTAAREAAELANRSKSEFLANMSHEIRTPMNGIIGMTSLTLESELTRQQRENLVIVSNLAHSLLTIIDDILDISKIEAGKMTIEQAPFSLRTQAFGVLRTLAVKAHQKKLDLIYNVHNDFPDQLVGDPLRLRQVITNLIGNAIKFTTEGSVVLDCVCKNKTDVGVELQFCVSDTGIGIQSDKIEVVFDTFCQADGSTTRKYGGTGLGLSISKRLVTLMGGDLWVNSTFGKGSQFFFTVRFNTGTMSVDQINLKTKPYSGRHILYMGTMRDDVISQSVMRTLDELRFKATHVSSLEQAAALVPQSGSGAAQDKSMFDVVIVDNVKDIRKIKEIGMLRFLPIVLLSMTTPYISMKVCQELGIASYFNPPVQLPDLMNALLPAFESASALPSDAEHAIPLHILLAEDNVVNQKLAVRILEKFGHKVTIVSNGKMAVECYDNKHFDLILMDVQMPIMGGFEATQEIRKLERLKGKGDHLPIIALTAHAMIGDREKCLAAGMDEYITKPLRVNELIATINKFPPKNCPDLAQHDPYYDYPHQRSSYSPPHDTSAHFLFKKP

>[MeHHK3(Dual)](http://genome.jgi.doe.gov/cgi-bin/dispGeneModel?db=Morel2&id=153930) Morel2|153930|

MESDHRSCAPTEIDPDTQRVAASLDTSDMDTENSSITTTPSGHPLVDNGDFLFEPQTIDSNNDDDKARNADSDLFTSVAAQQQQQEERRQAHQQQKEEYYQQQGQQHLINSNNATQSNSINNSHHHHHNHHQGPSNPLRQKITILHPALVAEQLPSTKAPTVYALASSVPFPTVNQLSINLPNQPTASEKIAVPFKTILHTPTVNVTSERKNAPSGVSIDELSPVVNTMPSDTMDTDSIPEPPSQIPYASSTVEVGEQQPIEDDVHPYFAASNLPLRPRAHLPNGFSTPSSRTASPLSGSAANEWSKIDTGLISPPLSTTFFTPPCNSTQAPVHHAVHDQQLDPNLVQRTAEEQQIQSDAAATAAGESVSIGSRTTVFGFAPLVNPGDQQANMKSSEERALSDGHCQTMPLDTPSRQFDLAGQTAPAVDPTGVPHVRAGISPTLNLDQVETRPMEYRNEPSVESLVNSKDWSTTGLGPRSSWPVELSVLMPTMMRSASPLAIYWGEQCHLIYNDAWRPILKQKHPYAMGLPGANVWSEIWDVIGPQMAGVRELGRGSDNKGLRLDLHREGYEEECYFDFTFTPIFLRDGTAGGILAFVQEVTQSILNQRRLMTLNQFSKQAPLIQSVNGAYSMITTILQESNNLDVPFSIVYKTLDPTKEVVPTLTHTSFLPSQPPSFSSPSTHDDFLSTESIAGTGLEPGQGADCTSSTVKLIRSHFSVGVPSGKKNSRRIPQSAILCSTSFDRNLQVVDYGGVKEKVFSKISSTRHIPDSLLITPEEYEPLDPASPVYDDPWTWPVRSVLADGIPRLVTLPKSTHKLARALLLPILENPSVLESRITTVLVVGINPFRMLDNQYLDFLALLVANIASLLHFGRSREEERKSTEALYELNKAKISFFQNISHELRTPLTLMLAPLEDVLNQTPENSPIRSNLEMVRRNSRRLLKLVNTLLQFSRVEAGKGQAIFEETDLSKATREISANFESVALGFNLQYNIICESLDGLPGGIWVDRAMWSGIILNLIGNAFKHTWEGSVTIHQYPCKGKNGRDGVAVDVKDTGVGIAAEHLDTLFGRFNRIENKQSRSHEGTGIGLSLVKELTEIHGGVVSVTSVVDKGTCFHLWIPSGRSHHPNAQIKLGDSKEDDPLLRAPRDDILNNRTDASMFVEEATQWISQKSMPGSTLTASGTDSTEEEKLEEYADNIGVEYMRDRQDSIEMSNSDYEVSTMNLDEGEISKILMDLPDLAHEPTSDLDPLLPRRSSSVAHSPKMSKMDSPTPKTLPASESLFGPIVTDHGAYGGGQIQRNEVSEEPKDDSPMHRSKLRRSYIVVVDDNNDMRAYLREILGKDFRVRCAVDGLDALRVISERLKQGKRIDLVLSDVAMPNMNGYELLQRLRNDTTTMMTPFILLSARAGEEANVEGLDLGADDCLVKPFSARELLARVRSSIRLSDLRHELIREQRHALEMKQLIYSISVRIRSGLSLPQILDTASMELFKVIRCNAIRICRFRGVDEETGQHLVRYVAEISKVGQPKALTPMDRLYPKGLEVPETRVQSGEHDTASELRTVTNFVHPVYGIKSFISVPLIYNNKIWGYLLASRDADTRDWSQSEKLLFEQTGNQISLAIAHATLWNLKKSQQVEMEAAHAANEAKSQILANTSHELRTPIGAIVGALSALEDTDYNLTGEQRDMVKIMQITSDVALSVINDLLDTAKLEAGAMSLNIKECPALTETLEQSVRIFGDKAGRKEVDLIMEPSEDLERLETLLQQGMSIWTDGDRLQQVIMNLIGNAVKFTTNGKVMIHFSVAKNASSSLPGEASADGSQSQQATNPRVHYPEPFPPDAVVTPTTFHFEVTDTGIGIDPDFLKNNIFKSFAQVDQSMTRRFGGTGLGLAISKHLVMMNGGILGVTSNIGQGSTFYFTWPVALVSVSTKPRPARSLAVNPVIPAELALETRAVVVEPVTESRQQLGWILNHQGVQVTLYENFDTVVQDEQARNPHLLGVDGTVLAANHRPNAHFFLCTRSNTVEATVETARALGEIFKARNAKAKMEGDQHYRDHILSIVLVIFSSPQGRSLARDMVKRIRANGLEDTLHCRYVVKPVKPDRVVECLQMIDSSTSTQRSNGSSSTRQQRQEASAEARRQVAQSQSGNLTDTSASQVIPADGNLSLVSSDYDTDNYYNKNTPESSSLDPDGEADTIAGGNVNKSQPHMNGTLRPSTDTGSESDRKMDISTSKPLFTPPQRMRLARTTSGPKNSGGTSTDDSKPPTRSNPAFSNRAARAAAGKRERKGKCVLCVEDNIINLRVVQYQLQKLGYDTLSACNGQIAVDIINSQVEMLGERTASLRLDGEESGVGNTDGDVSMDTADEGDQHRHHLIKGGLLVIPESNGNMSHIFDQTEPSSPSNLPGLSTFSAASTTTGSPLLSSSSAHFEGHAAPLASTLAAALGASTYSPTMQPTVSSGSRPPKIDLILMDCAMPVKSGFEAASEIRAIGQASSFAAQIPIIALTASAVPSTKEKCLAAGMNGYLSKPTKLADLEATLDQWID

>[MeHHK4(I-SGD)](http://genome.jgi.doe.gov/cgi-bin/dispGeneModel?db=Morel2&id=1828054) Morel2|1828054|

MLPTPTTSESISQNQESTLRLPTLPQRPPIMRAVTSPIPMPNAYSMQSGNNQPLWNPHTLVKGTDAQDIESASPVDNSLGLSRPGPQPLHAGHQLSTPPRSSLTLCETTTMRSVSSSSSILQITPAASGPGHCNSSSPTVPSNHKSSITDSLKRQWSKGQAFHWFQDAQDVEGVTPKPALRPSPCDRRQAETLGLSILPSSSSQMSDQQKGCFKLDSISISSPADDGSTNRMGQDSSVGQPRMGDTRSRRISSSVSSTSSTSTTPSIPSAQVQPSIGLAESLTNLAKPKREKSPSSSDKRTSNKSYQKAKNHATRQRQPSTQQGPTSDSDYESWDDFLKAYSQGHFPDDAQIRPRHLGPDLPPFPPYARALTQSQPPPFLAAPLPPNEERRLRALYSFQILETGTDLNFDRIAQLVGTVLGVSGCMICLVDKDNVAIKANYRAANMECRREVSLSGHAILRAPDDPLVILDATKDWRFNGLPAVRGGPLVRFYAGAALATPDGNNIGSLCVIDPEPRATFAEKERLLLVDFAAVVMREMTLWNDQVSLCTRTRMMRDITCWVHACLDMTDGVSMPTPTPSPEPEFVRRRSFDVSQARFTPSPDRARTPGSISTVTGSPEGHVQIPYPLSDAAIFPTPSGSPTFPSKELKANGRATQLQCINGSAQENSPTGDRLRDKAFPSACNMIQATLGADAVYLVQAGPNRSFLPQSGSEVSWNLWSSKTPTNGAVGTVGGAESLMDHPGLALECLASSSPQLIDKPVRNARRQGTAWVCAEAGCRPHRIRDFQQEMADPVWERDLPVISEAMCYVRQLKSAPERQPEQCPLYTFSPESEENNWFESTRSSSRSSARLGSSVCPGLLCHTFQGTLPEFEAGSSSPYISCVIMPIRCASSADYTRVKDDEPWAYFVVLSSSPTKQFLPHERIYLKNFGSCLVTEVTKRRIEAADKAKGIFIKNISHELRTPLHIILGILELLQANTEETLTEQQLTMLGSAEASGKGLIDTINNIIDLADLDPNNQTDVERGRKQLSDLFANVSEIDIRELCEEVAGSMAKRCVDKNLVINTSWSPAPLASLSSSISSMPSSHSAAASASASAAGAMARALSNQALPQAHLASVGDSVRDHSTTDSKAGLTGCNMWPDGHTSSLELLVAMDEPEETPEEDTHWKFMLNLPMVKRILTQLLENAIKFTKTGFVEISAMSPPLSMFPLKPPQPDARPVLFTVRDTGKGISQEYVHSHLFERFSQEDPLEAGTGLGLALVKLLVESLGGWLEVWSEGVEGKGCVVRVLIWATPSEKSEGVRSLRDEVGPWRGKACRFFVGGSAVGSDRLWTIMGKRMMGEDFGLTVERGQEQDVGAEEMLKGLKDGSPCDLLVVNDDLGRLEAYLNHWRDQHQRDSTVQDGQETALIPMPTPLLMLTTVSKEKKARAMVDAYLRTWKESATPLDKPIRVVILSKPTGPLKLMHFLRDCFSDSTCKVKCVNSTTSNKSGLDQGPQTLAGTTTLCEQGRANIGPCKVIRSSSSPRTMTLSMMGGGIGNNAQDNISGLFCPAESVIKSSFKFPPRSVTPGLTVGGHLAVGIGYFDRPYSPGGLRLPRKNEEDNHDVEDREEKDGLTAMTKPDGQKQEPGLGSFLVDHRRPLLIPTMFAKRRSSHIAGDLLGESLQTPPQPTPPQPHPDQQAQKREHGHGHSQGRGQEKPVVKQKTRRSIRKFMGSGHLNGPSRCGDGKIGTGASLAIGMDSPSLSSSTAAMSGQQALVKDSVTATATPVNNPSNSTCSNDASFPWSNLRILIVEDNITNRMILRTFFKKNGVVTVVEAENGQIGLHRFEEELVRQGPGGRAAFDFVLMDLQMPVMDGNMATKRIREAEGKWIKALEGKYCPSTIFALTGLAGEEDKRLAFECGVDGYLTKPVSLKGLANLLTTCRPPPPPLCSP

>[MeHHK5(I-SGD)](http://genome.jgi.doe.gov/cgi-bin/dispGeneModel?db=Morel2&id=1828755) Morel2|1828755|

MAPAPSTPSSTNATNEPSQDQTTQDNSQRRRPRFLRAITSPVSVNHGLKTTPTTSSTSSSSSSTLHASMDHTSPLSGSMAAASSASGNITFNAANNNATSSQDFASQEQLTRPSRPQLHPVQVDLQQNEGQLRSFSTSSVLPPFAVHPSNVPASQSRNSITETLKEHRWSKGLKLNWMSAFGSRTGTGLDGSLQTSSSISSSSRSSQHKSIQRRKSEGTGLRISRTDPGVDDPISGPGLGLLAPVTTAGKSSSRSGKTGLETSVDQLKVSEARTRRTSDSGLSTSSRNSGTGLAERLSGFAQTVRGLSRSPFTAGNSSPTMTSSSSSSTPTTPGSNTPYTSGPTSCRQQTMSNMDPDNMQFTSWDDFLYHYRQGLFPADHPIPRPHVDTAYPPTPPYAATDSPLPYLAPPLPLDEERRLRALYSFQILQSGIDPNFERIVQLVAMVMGVKGCMITLVDHKELTVKAQHPPSGFDCFPREGSLCGHTILRAADDPFVVLDTKSDWRFQNLEAVNADPYVRFYAGAPLTTSEGLNVGSLCLIDDEPKSSFTERERMMLIDFAAVVMREMELWNDQVQLCIRNRMMRDVTRWVRGCLDFGAPDSPVGDDPASEDTRLRGAHPLDSLQPPLPTPSGLSASSSGTLNMADGPALISPPLDPVFPTPSGSPTLLSRALGATTKSASQAASTTSGNPLQDRAFPAACILIQATLNVDAVYLVQASSNQSIIPPSGSSVVWNYLDAAGRRKGSVGIVKGGHSLGDAPNTALVCLASSQKNTDIFSHPVLDEKVQHTKRQGSAWVCTDEGCRPHRLGDSLLNAIEPVWERDMPVIKEMLGYVRQETPVPPQMPGQTRLFSYSQGQDAIENDWFGASTSIPSLIKDSNRRRLLCHTFQGTIPQLSSASNTPYQSCVVVPILGPNMAHSQSSTDDEPWAYFVILSASRTKQFSFHERIYLKNFGSCMVTEVMKRRVEAADRAKGTFIKSISHELRTPLHIILGILELLYANPEEPLSETQLAMVYSAEASGKSLIDTINNIIDLANLDPDNNTDSQTIHGNRSATPSPESDEPVMEEIDIRELCEKVAESMAKACTDKNLIVIPSWTKPSLSSLTSSAPNSAPNSASGITLMRNNSMPTGGRSNPVTDDAANGSTSPAEAHGGFSSRKLRSDRKPVLELMVAMDEPERDPDHDTLWSFALDIKTITRILTQLVENAIKFTATGFVELSAIALTNSPIPMKPPHPDARAILFTVRDTGKGISADFVQSHLFKRFSQEDPLQVGTGLGMALVKELVKKLGGWMEVWSEGIEGKGCVVKVLFWASPASHPTKSLKDMDGPWQNMSCRFYAGESALGSDRLFKIMGERMMGQQLAMKVERGNEQDTSTEDMLKNLSDESQCTLLVINDDLIRLRAYLSYWSDKQAATTEEGEAIPETPTPLLMLTAPYNLKSVQKLVDEYLEVQAESDGVIRPASVVILNKPIGPLKLVQSLRDCFTPAFSSPGLATPQGSPQLSPRTDLARVPPMPMIRSATVPHITTTTLGVNDSRLLSAGSMIKQSFVFPIMQNDRQPMIFIPPHSPGGLVIPPRVLSVDPSPATTPERERELGPNHQSSSSSSSLEDISRDITAKERADLEAFRANIEANAPKIPSIKNYHIRRQLQLDRAPAVGPTIDTSCSDRPLPRVLIVEDNITNRMILRMFLKKRGIGVVEAENGLLGVERFQEEVWRRDGKAGFEFVLMDLQMPVMDGNMATKKIREFEQGLVRQGQLNVNRANGFQTGPPSTDQEGAYRRTTIFALTGLAADEDKRLAFECGVDGYLTKPVSLKILGELLETNPADLKIPA

>[MeHHK6(X)](http://genome.jgi.doe.gov/cgi-bin/dispGeneModel?db=Morel2&id=684347) Morel2|684347|

MSSTPSSTPSSAPSAPLRTSFSSSTTTTKNSQTTNEPATATPTATITEDNLAVITDCPNATNAAQWFYKPYLQPSAISTTAMSTFTPLEPHHKKNKRSFSSSSSSVSSSTIPLPNTPASLYSNAVSASRLQSQTAIGPGHDPVGGGGIPRIRGEDLYSTYSTGVGTGGYNKRTAGPYSSQSTPSLTGGSFHLNPQPLLPIKDESIYHNPYRAPSHTNTRSFVPPMVQGYEFIGGSQAGNMGLMMGKRISDGVPVTGKLHQSRVLLQHEYRILKRLLIANARYDPTSTPPPKFNPSAEPQTKPEPSSCGETSTSASRSGVCNSAGATTQTTPPTSPHSTATPSGRQQPARESKCHAKPGVKQAVYETGSVETEKYFNRVVEDFIDLDQEDLSILILERLGPNLLSHTHHQFMGLDNLQELYSADGKIEKCVGSPFRDVYTFLVFALKAACVLEALQSSNIAHLAICPTALHWMPAEPKIGPWSPNTDAQSESNGAGNGIGSGAGYSSGGGASSQYTKGPSTLYSLLDTMDINDTKLRLFDFTHSKILSHERARAPNNIVEWQIPGYMEYHLQFLAPEQTGRAETWMDYRTDIYGLGATLFSLLTMQYPNTGNDSVQILQGVLTRDLPPVSDFRPDMPPIVDAILRKMTQKQPSQRYQNAFGFKQDILRCLNILHRTGKIDPFELGNHDISYQFVLPNGLFGRHSEQQLISAAIVQAASAYQQALNSELISSDDDDECYGDFDGGMSCGRPSTLASRNTNDSEDLYVFEDDIVSLKCQLSSKIPLSSKTLLRKPRLFDRGVGKSVHRGTELSDPVVRVIFVSGPSGVGKTVLIRRMSTVARNSGLFAGGVFEAGNSAPYSAILSCIQSVLQQLLTQQTDALASLVIAIRTAFEPDSGIGIICDLVPELKYFFNGSDLPESKDVPLTHSVARFHALILKLIRVISTHFFMTWLIDDLHFADENSIDLLATLVNVNKRLPIVLIITHRDTVDCLIKVKQILGGGNVAGARHPTGFSALGTMQNEFNLGSSGTGCDLMASTPSGSGNMGGGGSDGKSSLPRRSLNRSSSGTSLIVRGGGGVRFIRLQNPPLEAIQEFLASLLHRNKEDVVSLAKALRQKSWLTIRQLVLELYRTQTIYFNCFSREWEWEPCEETLGEIVRRLTGEEFAFLDQRFRALDCDTKKTLICASICGPFFTIRDLQLLASAAFTWSGSGPRTDASDVSAASDSSSTPAAVNSGCKAMAGLQSAIREGILVYTSEPNELRFHHGIMRRVAVNLLDSPEQTEKLHFEMAKILFNTPGQELRTASHVLQSLNLIKKSLAAGKMSKSLMAATLEPVVEADKSNDHKVHCFSGNHSPSVLEEEDNKSSELDGRALRVILSLAGEKSQKSGAQDMALAFWNAAMTLLPEHCWVRPDETETVDGSINNATSTATTSPSCSSSVHSLTSPNSDLQDTDMEELLGSPKSTRSSLRDDARYTLSRQLPLHHEALKLHLQCIEAERWRENFGQAMKMCEIVLANVTDPIDRARVYQHQIEMSVWAYSSPEKATAITIKCLQELGMPEDTQFNPTEEEMMVIFGETHKMLLRHMPELEADPPKICHDPKIAMMMEVMSVASASLYYCNVPFMASGIIQSMRLTCEHGITKDAGRALVSFALTYSTWYGCLDNAYELGKLACDVSNGNQHVIFLFYMTVQQWGEHIAHAVSALEETLTDVDLTCDRLFHTAGMIHVAVIKVLIGRVHLNDLMTSTIDHLAKHIEFGPKSHGVEVMQGVLQLVKCLKGRTQSTTNPELIFDDADYSESKAHAAKRSKNSLNVVHNNSTYQMLKIVAAFVFGHYAFIEQMTDVWHDDPKSIMNFEGSWIAHSIFTVIGLSLANLLRTEKDPEVRKRHQRRLLSIRDRMKMRANKYATNHAAMYYLLEAEMADQRLCQEEEYDDDDDCDEVEVNMEVDDDDEYAAQCRVYNVLSDSRRRGRGGMFIPALKKVFLLYEKAIKFATDGDFPLHKCFAYELAAKCHLRHRLFTSARCLMANSCKVYHNWGAKGKVQWFHKTYPEMFPAPQDDDPAGLARSMFYRPTSDNESSRYAAAASSSCPVANATAAASSGSFRQAASMANAASVCPLAAAAAASTSPWMTASPRSTMSPSDVGNDALNAATVSAAADVCLASTFQAGIGGGGCPSSSSVANGPASATSTGTSILSAPLYSLQNTWINSPRSPEVENVDLDVIDFSSVIEAMQVIASEIDLELLLVKSLGVLNQSVGAKRCSVIISKDHELVLAASLGGDRGRCESVNPPMRFDQCSSLFQGVIHYVINTSAPCLVVNAKDDPRFCADEYLKQHVDLKTVLCAPILHKTALVGVLYMENFPERAFANKRLLVMNLLVQQLGISITNALLYQSVLQSETKLNGLLENMPCGIALWDANAENCQYINSTWGDMTGYTVNQIMKSRWSILVHPDEIVGHGIAWKDRVRAGESCQWEARYGLSDGSYRWGIVRMLPIKANTERANILQWLTVTIDIDDQRRAVQLKSNFLANMSHELRTPFSGILGMLSLLKDSSGLSDEQFEFVDMAKASCEMLIRIVDDLLNFSKLEADKVTLEYIPLCFEEILGDVCDLLVPLASRKGLELIILLDNTLPVQLIGDPDRMNVVIKYWHEIRKRETIPANKPPQSVLDILTGKCRLDYYNTSGVEQLRRKVALESDGSHLGDEVSLHCSVTDQGIGMTPEEQKMLFVSFQQTDSGTTRKYGGTGLGLSICAQLIAHMNGKIVVDSEKGKGSTFTFTAKLTTMTEQDKAEHPAENARIQECRVVLEKRVTALKDKRILILSPNTYLREQLKQTIGEQVQFMEFDSVDSAIEARAIGVLCDSEEGCPELADASSSHSGSEEEEEAEDCLPVTQAERTCKGSVASAGQCQSQEQNLSKAPQSNNEQEKLKQSCKGQSGQGGGNCQKNQPATSTTTCNSNTPSPCSGKTCSQDEQKPSGSPCSKDPSKKGDSPCKGKSSETSPDLPQIEQFDFILVDHVLDSAELDCIHPSPRVAYVLLLAPTTETLRWILPPATEKRYDANLDETESEQVDVGGRGRIRQSSEMDFAARAMGRVHSASYMSKIDHSISVSAPIAPAKAPICAGVKSLAAGHSTSGDASNATAATATKVAGLTRLPSQLFRRRKNGVSHQGHVRKVLSNSKQPSFQVVRMIKPVRRMKLLQIMSNALMQHEHRQSHPEEYTEEDEDDRWPTGARLTELDEDDSSMGEEYNRSRRGSYVSTPTSSAYSTPAAPSSPMSDSSSSRPSPSKTLKRQRSNLALTGEDSSAQSDSTKPDERPLPKSARLTTKSSTAAAAAAAAGRKKGKSRVRSRDMDMDQDNDVRKRARSNDALSLLLSPRQLNMCQGINVLVAEDDFVSQKILEKQLTKLGMNVVIANNGQEAVNQWLAAERGHYTIAIFDHHMPIMDGLAATRMIRSLEAEHKAEEEERRKLNPNNDDDAAHKDQSTHGEGSASSGIFKPNRNPNRIPIVGLSADIQHATKEKCIKAGMDEYMTKPLLTQGLAILIQRYCCE

>[MeHHK7(X)](http://genome.jgi.doe.gov/cgi-bin/dispGeneModel?db=Morel2&id=128130) Morel2|128130|

MLPANSPSTPSAPSPDPSPTETYSSRLGQVTLASILKQAPPPYPNVHHAPLAGHDTSFPLFTAAATAGASTVPPLPKSQLPIPLPVPPPPAIPTMQRPRMKTTGSSSSLTDSITREADMHDPVPPHPRELDNSSSTDDVASQWNHRPSLLFQPSFVPNIIADNSSRSSTHPLGSISTSTALLPPGMRTEHDYITASSAHQRGTPSRSSATFPSTASFHLHPPPAPHSHHREGPGHDNPYKPPPHTNSSAFLPPRVAGYEFRGGSQAGNMGLMLGRRISDGLLVTGKLHQSLTLLQHEHRILKRFQIKDGRYNPHNNPSQPNPQTTAPGSDGGTSGSGSSNQTTPGDCPTEYESGLVEGEKYFNRIVVDFVDLDRTKMSILIMRRLGPNLLSQHHLRFTGLDETDGRQNPELHEDRMDGSPFPDVYTFLVFCLKAACVLEALQKSNIAHLALSPTAFHWSLPESYGAQQYSQGEETVQRSAPRNPSSLYHDADSFDSDRVHPYSDQGAAGNPSTTHSGSGYFGAFSQLPKWDVNDTKLRLFDFTHSKILSHERARAPNNIFEWHIPGYLEYHLQFLAPEQTGRAETWMDHRTDIYGLGATLFTLLTMQFPNRGTDSVQILQGVLSRELPPLRNFRPDMPPVIDDILRKMTKKQPSQRYQNAFGFKQDILRCLNELHRTGTIEQFPLGKHDVSYQFVLPNTVFGRHIEQQMISAAISQAAAAYQHNNDASDSSSNEDDAPTPKMMTMDDDHYVFQDEIVSLQNQASAKISLSSKTLLRTPKSFDGGKAVHRGLETTDPVVRAIFVSGPSGVGKSMLIRGMAPVARSSGLFAGGSFEADESEPYSAILSCIQSVLQQLLTQHTDALASLVVAIRTAFEPNSGIGIVCDLIPELKYFFNGSVMPESKDVPLTHSVARFHALILKLIRVISTHFFMTWLIDDIHYADENSIALLATLVNVNKRLPIVLIMTHRDTIDCLIKVKQILGGSNPIGNNPTGSYGTLGSIHHDPLVDEISSLNTSMSAPAPMTKTTAIRATGLPLVVRGGGGVRFIKLQDPSTDNIQEFLATLLHRTKADVAPLAKVFCHKSWLTIRQLTLELYRNETIHFNSLTKAWEWESCAETLAHEVRKLTGEEYAFLEQRFRNLDCDTKKIIICAAVCGSIISIHDLQHLASTAYVWSAKGDQGTYPHEELNYAPETAELDSNKGSSAMAGLQSALREGILAYTTVPDHVRFHHNIMRKVALTLLEGTDEKERLHYEMAKILLNVPGQEFRTANHVLLSLDLIKKKLAPVDPDVPKEVPYWVPVVDEGAEAEYPELDTESLRAILSLAGEKSQKSGAQDLAMANWYAALTLLPENCWDLNVTGTAKESLESSEMDVDEAKDSVHEVPQVKTPLYLEALKLHIQCIEAERWRENFDEAMSICNTVLDKISDPIDRARVYQHQIEISVWAFSRPDEATRIAIQCLQELGLSKDISFNPSQAEIRKLYDETHLLLLSHMAELQAENPKVCKDPRINMMMEVLSVANSSLYYCNVPFLAVGVVESLKLVCEYGFSKDAGRALVSFALTHSTWHGVLENAYEVGKLAYRVCKDNHHVKFLFHLTVQQWGEHIANSIPALEETLTASDMTCDRLFHTAGTIHISVMRVLLGRVHLRDCMTATTDLINKHNEFGPKSHGVEVMQGILQLVKCLKGQTQSENPETIFDDNDFAESKVRGKVDKSLILVHNNSTYTMLKIMAAFVFGHYEFIDDITKSWHDDPKSMMNFEGTWISHSILTVIGLTLVNLMRVEQSEEVRERRRRQLLAVKDRMEKGALKFPTNHASMFHLIEAEMADQEEEPKRDLKSILLLYEKSIAYAIEGDFPLHRCIAYELAAKCHVRHGLVTSARCLLANSCKGYQIWGARGKVLWFYKTYPEIFGTPPEIENAVPPRGFFYRPSDSPSGRRPRANSAAGAIPTGTSNVSSTIVGAPSHATTSPWLAGSPKTIYSPGGYGGDAVSAAAAAMAESCLSSSMQSGASTTSSNSGLLTNSSWIASPQSPEMDNADLDVLDFSSVIEAMQVIASEIDLDLLLVKSLGVLNQSVGANRCCVIIAKDQELVLAVSQGDLGRCEAVKPPIEVSQCSSLFHGVINYVINTGTPCLLTNAQDDPRFCADEYLKQRSDLKTVLCAPIVHKGAMVGVLYMEAFPQRAFANKRMLVMNLLVQQLGISITNAMLYQSVLQSETKLNGLLENMPCGIALWDATAENCQYINSSWKDMTGFTIDEIRDSGWQILTHVDEVEIYAQHWRERVRAGVPCQWESRYRLKDGSYRWAIVRMLPIKSPSDNKIIQWLTVTIDIDDQRRAVQLKSNFLANMSHELRTPFSGILGMLSLLRDSSGLSQEQFEFVDMAKASCEMLIRIVDDLLNFSKLEADKVTLEYIPLCFEEILGDVCDLLVPLASRKGLELIILFDSTLPILLIGDPDRIKQILMNLIGNAIKFSTTGNVVIEFWHELNKKKPRRSTAASQDLLKIISGKTSGRVELEDESHFGDEVILHCSVRDQGIGLSPQEQKLLFVSFQQTDNGTTRKYGGTGLGLSICAQLIAHMNGKIVVDSEKNRGATFTFTAKLRTLTDYDQEQNPAETFRLKECEKILSIRLDLIRDKRILILSPNRDLREQIRRTLQDTISVEYETLEAALESGVIKLTGLSNSVTKSSGSIRVDVDAKDDHSGLGVPAEDEIMTDGYRDSLGLEALVPFDFIVVDHVLDSAELDRIYPSPAIAFILLLAPTTETLRWILPPAAKEQPDEPEESGAIDVGGRGRIRREGDAHLGDKPVGQVQRVSTASATDSNISNPYLAQSYIAARPSSSGRSGNAKSTLKVSSELFKKRKSRGQITVTRPVPHYALQGGEEPKMETSTFQICRMIKPVRRLKLLQIFYNAIHQHNHRLQREDYQDDDGTESVDSGYRRGSTVETSTENDSDSDTGVDKKRHLSSMDTTSESHSSTPPPLLRSRSNSPMSTSSSSGRSVSLKRRRRQDTASDTNTDPDLKRVPKAQKSSADDTVVMRGGSATNPTVVDAQPMLATVDAEIDPPRNLGLSGAVIGVDDKSFPKRTRNNDALTLLLTPEERNSCRGKNVLVAEDDFVSQKILEKQLTKLGMNVMIANNGQEAVNQWLSVERGHYTIAIFDHHMPIMDGLAATRKVRALEREFAQEQNDGKEPIRIPIVGLSADIQQSTKESCIKAGMDEYMTKPLLTKGLALLIQRYCCADRRHESES

>[MeHHK8(X)](http://genome.jgi.doe.gov/cgi-bin/dispGeneModel?db=Morel2&id=1837074) Morel2|1837074|

MAPDNTSSQHSNSHEPDIAYNSDKNCTTPPPDDTFIHTLSPSPLPLPSFSHTMTSSTHGFTHSHSRSLSQPAIPLSQEHPLAFLPHQQQLFDLMAEQESSSSADSSSYQHHLHHSSHQHHQNQTQQQQQQYQQHNLDMSRFLDFAVEASVLLETLHQNGLIHGQLCPTSFRWEESSSLKKTIELASNTTLTTTTTTNAHANASSTSVVLPPPTTSSSNNSSTASKAEQDYALLTITSTLTGNSPSAVVTSTNAPSSSSPRTSYQSHPSRKDNHTSPASLSRPHTASNTPNNTNNINNSSSSIHGNTTGNNSSTGASNSSNKPGRRQRRYSLVLDCTHLGLGEKSSEPPDSPARKGLRDQSHSNNSSNNLHHPFASPPQSASSSPSAATLTNGSFENNYINRASSNNASPNPNAGSFLDPLLFSPSDANLLPYSNTNSAAVFHDAIATRMARIKRSLLPSSSSSPTLGQSSSHSSSTPSSTAASQAGPTSTGASGSPLPFVLKRHFLLHVPQELHSPPGCVLPVQIDIYTLGVMFYYQLTSYTILLPEDMLAGSIEHSLAASSIHCGHGQQHKSGARGAVLASTPSRLANVIQRMVAKSSKDRFLSMIQVRKELSLIRDQELEASNREAESKALARTYSDNINVNAGITKPSQTHLEPSSYPSSGLARPSSPANSTHSFKSTDTRVSQEIDTSATGARIPAASNASSSSHSQETNQGGLTHEDRAFVLESVLNIGFNSDLRVMLRAISHALDNILVGHPPEEMAIILWQKDEHLPNGGTWAILEDKFQPGATNQSNLTWTDLSERRDHMPVLVRKALDTQEPIFSTAVGSRSQLPTIACVPIVAGPQQHQQSQQQWTTPGTSSPAPSVSSSISSSPTLVGAIYLHHLHPRFYFTKRDQDMLMLFCQKLAPSLQHCDKVSSLEKQLALATHRSRVLEETNARIRKNEDEVFSWMEALPCFVWAAEPDDIASRRYLSRSWFEFTGFPGDKRTSDRWISAMHPDDVAAFQKEVSQSYKTGVYKDCEFRLMRFDGVYRWHLSRAIPVLNQYGAILKWVGVTIDIDDLYLAQKAELHKKSNFLANMSHELRTPFSGFHGMLTLLGYSSLDEEQQECVYTAKASCEKLLLIIDDLLDFSKLEADKVTLESSPFDLQEVFDEVEDIVESLASQKALELAFLKADNVPDVLNGDCNRLKQILLNLVGNAIKFTHTGHVVVKCRVLDRDTDMTALSPGSSSSIDDDKFYFRHEKYGFGQCQSEMRPPNSGRFSSPEPLSESSVKLMFSVEDTGIGISLEEQEVLFSPFSQVDGSATRSYGGSGLGLSICLQLVKLMKGRIGLVSERDKGSTFWFVIQCEQGTAAESPIRAVPETDVVDSTKEIKRITRTLGTPRILIASTSEATISTLQSYLSDFNTEVANLPSTAASRLEESVVNGIRFDFVCWDFPKYDPQHAKMLELKSRPDLNNVHFVLLYTPLPSPDLIRRAQSLQLPPSSSSVSPSLGNRKRPTLSQSVSLRLDNNGSVVGSANHNHNAGSTTECEVPGLSPEKLNSLRITCISKPIRRLKLLRAFVEILDDSTKIHGSHAVKKAAVNNAGGATPIPPASPTASSATAINSPISSPTNSTGSSTTLSPVGMVRSPSASMMATLDGSKTKESPSVKESASTTGPIAAPTRPSFPNADSAAQLLSKSQEQKVISQTVAETGSRDALDSPVNLNESSSNNPGHRHSSSSANALEAVDEVTALEGSVFEHHHQRQRSQSMSAPSSVASSKTMFSASGNGGDVNGNDSSSTPHHNDKGGDAHEGDKEKEKEERDRLSSPPPVKVLPVKQKLPVNSSRASKLRSNSPKPTKTSKLVTEEATENSLSLEEANRITGMRILLAEDNKIAQMVLSKQLALFGLVISCANDGADALTLFKSHPRGYYTMGFFDHHMPNCDGVQATQQIRLLEREHAAEVKGPVPRLPIVAVSADIQETARKACLNSGMERYVTKPLMQKDLVAMVRHYCVIGDAEASTHAYVSPPEGAGVSKGLGVVSSSSDSSAVTSTVEAMVSAGHVSAPIGISSGPTGPMLLSPSPIGPQLHIGSPLQKQPTPAPKRELELSPAAMRGLALIRETTLQDESSKVGGGGNIKAMNLVMSTSMATSASTGCLPTQQQLLQQQQQQLQAASGVNHLGLRSHGSQSQLRTPTTTFSLSKSISTSSLSGSYHAGAASGGIISSPMIMSTSTPVVTSSPSPAPGPIALCGGAPNILNLSSSFGSHHHHHHPIAAMAAGAAAAAAAVVNTVSSAISEHIHPPSIYAAQPNSSALSSPWKGSLEGQQIQPDQLQQPFVLPSFSAESSTEQVPETGKETETGGHTFHPPQQVFVPYLHPCEPSPTMDRDDPILTSGSLSAAALALGSSATSAAASNPTSTSSGDSADSSSSGAAGTAAGVAGVQAALSATATASALTGATAKAVAATNATMAATATVQLNNWL

>[MeHHK9(V)](http://genome.jgi.doe.gov/cgi-bin/dispGeneModel?db=Morel2&id=123498) Morel2|123498|

MDSSSSRYDSEGSYFPPVESAMSGAFSNAANFVAAQSKAGYADFVQGLNPTWSQSPAPSPNIIKEDPTINPFFFQPLTSYPAMDDDTFDPPDPLSESTEDDSCSPSDHKDPQGRLCTSSRPPMSTGESHGLPSLPPLPPPPPSAMAHARSLIHVPLYRSTDHNNQRYLPSDIRHHRPPAMPVAIVSFLADQIPYPPEALDILTELNPFLANQITNALRLEDMVARSNRWGELSTSGMSESSRTNETFADSIRRKMKDTNDMTPSDLATPKADHPPVNGKSGYSRSKQPSLFGQSNSADPVVPASISRDNPLSTPTISSTTPSAAPPKRPALRRDMPVGDLSVVMDRQATSTKSSRTKPHDADAQSPSTFTRIESDTSVASTPSDSPFNPFMLQSPDPERFSKGDPLSDVKQPESFYNLDSTTDEIHRSLNLLNLDITGSATCPLAPQTKRMLGKQHQGAKGTQQHHIDGAVLNNWANGHRRAESSPNMPETEESDGPDGMWQTLQTEETDKKPVSLPNPRTTENDLSLAGQESPYFGSIHSVSPSGTSKRHLFSTSKRSGVRRRRKSVKDHAKLYQPQVPPTESPVPSSPYTPSMPGFAPHQFDASLDHPGSSRPAAFSPEPSPVASPSAQYPRTFDRAFPGQLEAGSFKSQWKAPTREEFEKERAPLSRHGSGWSFNSDDTSLASPGLHHDRHATGRKHKKRHHGFAEKGGPAQIPRTRLLRMIVDAIAHHVFTLSPQTGCLTWLNQRSMQYTGIPLPHLLHKPWTGILHKDDEMKFQPLFKDSFEKGEMFNAQYRVRRFDGQYRWFLGRVVPVRDCGGHIVQWFGTSTDIHDQKIAELQLNRQVELELNEKKYRLLAEAIPQIVFTAAPQVGLTYANAKWFTFSGQVFEQASGLGFMDHVHPDDRAKCFLPTNISEDNIGPPSLGDQDPEYKPSTSPGTAFGQGPGEVSYQTEMRLLRKDGKYRWFLVKCISVEVVEQGRKWFGTCTDINDQKLLEHKLKEAHDAAQKSTESKTRFLSNMSHEIRTPLVGITGMINFLITTDLTSEQLDYVHTVQQSADALLLVINDILDLSKVEAGMMKLEMEPFSVHAMTEGANELLSTLAIQKGLELGFLVEDEVPEVVIGDRVRLRQVLINIIGVGALASVCFCVQVIIVIIFLPSGGSASMEGAKPNADPDVLTIKFEISDTGKGFDESERAVMFKPFSQVDTSSTRKHGGTGLGLVLSKEFVELHGGKIACESIKGEGSKFSFTFVARVPPPNVTARATTPIASEDIRVNGLMQNFSKPAFKAAKLPSGAGLGSGAGPGVGSFASKPPLMKDSKRVSDPALLKHVTGLKLIPGEMCFAEPDFESTKKTATKALESATKKIKRGDFLTADLTLAIDEEDQLEQRSAATTASTTATTVTATTESPPGDVATAMTRTESILDDRRYLKVGQLTPSNPPMGEPPAVAVIAASLPNPALDMKLAIPAKVLESKALVAENRKANAALASTTRPDVLPNSESSSSSSSSSYSAGSSIESKQSSMSTANSSVGSLTNTTGITSSTAANHAPEQARILVVADLLHARETILHLIKQLVPKKLDPLIDVATDLKEGVEFLVGGRKVATKAGKYNYLVINLASYMSVMDIFSAMQQDGVLVEPTVVTCVITTPIQRAAVMEAVKVEEASMEIAKSSLEASMPKVIPSNIEWVFKPLTKAKLHVALADIVTHQETLTSPNGTIGGGLGGLASNGKMSVKRRTAQQVVVSQKEIFSQMHDAMEGKNIRILLAEDDLTNQKVIRRYFQMVGAELVIANDGNECLDKFKSQPKGHFSLILCDLFMPGKDGYEATRAIRQWEDEHLEDGEQRIPIVALSANVMANVAEQCLSSGFTSYLSKPVDFKRLSETLRKLVL

>[MeHHK10(MS-HKI)](http://genome.jgi.doe.gov/cgi-bin/dispGeneModel?db=Morel2&id=1241069) Morel2|1241069|

MPTSRSNSISLPIHSQEPSVRPSARSTSLGHLLSLPPLSPLPPALANRPRALSIAATAISSTSTGAQLSVSLSASADKSRLDIHHNSLAISAEPHHSTSLLPGETRLDREWDDPSFSYLAPIATNLLTCQGDHFFDLVVEELSVNLGVKYAFISQLVSLEELKELDPTEYMNLIEQFGGLVPPLDGVMHNISSWPGESHINPHAFQGYLADCTIQDKVTFIDSNLADQHQDVAECLMDHTIESYVGMRLETVQGEIVGVIGIIHDRALTEEDSLIVKLVLEQVGIRVANELDRLKIESNLIYARDVAESTAKNKTKFLADMSHEIRNPMNAVVGVTDILLDTAGLSDEQTGYIEVIRTSGQHLLTVINDILDISRIDQDVKFLLERRPLSLRKCLKDAVSLAGLTPLHDVSRSISVIEWPPEMDDLGPFSELEETNILPLLWSIDHNVPEHLLGDITRLRQVLINLCTNSLKFTQRGRVSVHVSIHQPSAQYPTIRPNSMPMVTRNSPQNGQAYSPRTDGGPSSSSSPSSPMLTGSDHRTQMVFQQRYDVKTDSSQSTHTKPSPASSRRRQADKRHLPLPSPNTQPDQSSTFAPPSLTVVAPTANSLESDKMDENTVILEFAVSDTGVGIPANKITELFTSFSQVDISVSTRFGGTGLGLAISASLVEKMGGNIWVESTEGVGSRFTFTIPFTICQSSETESHPSATAPSSPVSTPIRHESAGRINLDTFQLPASNTSQSPLLSPAPASSMAEQPAQENSAKEEGRLNESRAPPSSVATSPTELSKPTVPTYAKVVESTMKPLTIDGIPLRILLAEDNAVNQKIAVGVLKKLGYENVDVAENGLEVIQKLDEGSIYDVILMDVSMPVMDGIDATKTIVDRRLRGLLSNAAETSNCDTLAPEGSQSNGTHASQESSPERGYKDYQNLYVIALTASAMGSDKERCMEAGMDDFMTKPFALLEMKRVLNEYIHKWDGGALQVRNETCVSAALAAIKSRCNTPNGSSDRALTPTMSKSPSAYSSTVSLADHDSTDSLCSLGCTNGTDTQCGDGHHSGSSSANGSSPPSSMGRSRIETPRPLRRNLDAIGCSLFSAGRRSHSEAVLNGLLGPVAVEGGLLASRRASDVFSLAKREWRSGLGLSNDNLNGVQDVHPLDPLVDGAQAYQDRESGVTSPSNSNLAPLSSHGANGFSHPHLRVKRVMSPSSLSKEQDFGGSFKPTASPLSPLSLLTTDTEGSSSVSPPLSSTSTASPTEATARRPSSGDETLLTSSREDSRFLSVSVPVKDKTKSESEATKAQVANGDVKEVKEVKEVKDKQQQQEPANGQCGPAPSRIV

>[MeHHK11(MS-HKII)](http://genome.jgi.doe.gov/cgi-bin/dispGeneModel?db=Morel2&id=1153927) Morel2|1153927|

MVHKTTASSPSSLSSPPISTKSSACCCCVVPRSNIPSLHRQPQPHLTRPPRRSSISKPMSSPHSLSSSSELSWSSMLSHLFAKDRCPRRVRTSSARHTRTSSTCKSSHHSIPSDYQAATATYFPIATATSSSSSLFSRARSARTLLETLPRSTSPLSLTKHRPQSSLLPSLASSSSKASRMDLALYLLLRFAILFLVPLALLQGTDAFPLESLRKPFARRDVTLVSPPPFNSWTVQSNAVAMNLSKIVACDPDSMTGYNGRLPLVSTNGSIRLAGIMHGTTTYWNRVLDGANDAANLTGVEIDWFLPKNKIFDPTFMANQIHLAANSGQYDGLFLTIPNSEIASAVMRVAREQIGMPVVVVNVGQQTASQMGYLSVLQNDTNAGEKLGYALYDRGARNFLCISPSQIVQSLNDRCAGIMRAFNNRGITFEEGSSYNKTIFVGPTNIDTPANLDRIRAYLQSHTSIDAIIGVSLAAVNLTLKASVLSRNVTMTPAVGRTGNYWVGTFDVDDTVVANVKSGAIAFAVSQTPYLQGAIPIIELFLQVATGQKLVEPVITTGPNLLTADTIEREYNLDMTASLQDFAKAQKTVVVLNRDIPLELTRWNEALGGLVQSASMLGYDTVSATSMDQLNKIHADLKSQTNVSSSVNYGPYSGVQGVVVSLADTTQYDQLMNNTAILGADMPVIGMGSVSNSTVLQSRTVWLGPSDEAMGSTVIKELFSSSYSVPLCLVEENGPWWQYKHCEQLYNTLVHMYGVGRVGPKDNMILRIKADAVDLAYNMTGSLAGDLDIDIDLRVGHQASAQAAPGMNSTAPSRNATQQILDAFGPSAVVAYDSIICTSLALYAKVDEAYNDLINLRRNSGESNGAALQTNAAMLAALPSMSKMSPDPNTPGVFVLGLSPKDLYSLAHDQQVTGILNPQQYLQGFHSILSLTTRMMFPSRAAVFNQFFNTGPVAMDYACSAGSFYSSFNQTPVDLGSIYGSVGANLAALASDPGLTGLTGATSMLCLDAQNRVLVQSMCTRCAKGKYSNSTDSHECTSCPSGQVTSGIGQTQCEICEGGECSGSGGMSIKTLLLAVLIPVFVTIASVVAVYCLWVRRKKSINNKKLNDDSWQLDLAKLLYSGIGDEPDGTFGFPRMGGGGGGGGGDGVGGGGEEGSTTNSHSHNRSKKKNRNSHNNITLPAIIVGSGSSQNTPSATRQLSVSESQDASDIPRSGSRAGSASISARSGSTNTKGMQGYSNSQFSLVMNRGSSAVGTWRSMPVFIKKIGSRKVTVSTELRKEIFNMRELRHPKLVEFVGVCLAQPNICIVTEFVPKGTLASVLANTDHKFTWLFKFNFMQDLCRGGRLTSMNCLISSRWELKIAGYGLNELYRSQQEGPEDHQTNNLPQIMHSSSTSSQQRSIGSGSGSGGFRPWSSDSDRSQRLNQHLQQQQSIYDHSHEAPSPRVRDLTDAMDKEERAFYDQENATAAAAAAGVPLTATSTAVSPRLHRNGSSRNGSIYPAAPLSHSGVSSMAEYSGIDYSTDATALLWTAPECLHLDKNGDYEAVGSQRGDLYSAGIIFNEILTRNLPYHDYNDDANVLELVKDQDFRPTLMAPTDGTFTEEDRENLEQMNQLIQLCLSKDPSSRPHFTAMLTRINDINPHKSSDFISSMSAMLEKYGNDMEELVRDRTRNLQQRTVELEAEKARTNRLVLDLQKAKEGAEAAATAKSNFLANMSHEIRTPMNAVIGMSRILLDSKLNPELAECAETIESSGNQLMTVIDDILDFSKIESGNLKLERRLLDLSFVMESAVNLISSQASSKDLSLIYEIDRNCPVEIMGDVTRIRQILLNLMSNAVKFTKEGVIHVSVAVETQHEVRFEGEEIINGAGNSKTTTTTAADPTRLMPPGSGGSGKRPSRKITSTETPATPPVVISEGEVQEDLQKSVPLTDAADMGRTSSGTLVPPQTKPVKLIFAVKDTGVGIPANRFDKLFTSFSQVDESTTREYGGTGLGLAISKRLSEMMGGSMWVDSTPDVGSTFYFNIVLDSPVGCPSYGQQFELSKLADKKLVIVQDCPKGQEEWRRRTQAWNMNNVKILGSDQILPYLREDQESSKSDGIISSTSMAKALTREALQEKIEALIIETELKGMVSATPEGILNAIQRSAVVTTHLSGPSRKSALHRRRASVRAASISSSNGDLSETVSPTALPLIPVIIFKNMRDVRTAASVSSSYHGHARRDTSRWSGERISSSDTGDEEAVSTSSGSIHHRLDPQGRWKVYGDLSHHPHNQQQQQSNCVDASTASLPLERSSPPFAQNGVEGGSQTGRLTYTTGPGNLLTPHTQATIYEHSISSVDHLSTTVPSPAPSLGRTGYFSNSDNESVTTTSPPLPHQGNGGVSGSMSYQHQRVMQCGGYNGMGIFTTPIYFTKPIRHSKVLQALGEDPILFDPRDFEEEDDEDDYNDIDEEDEIEDEEDHVKIEDASIQTNDNPNLFKNALRANASANPMALLPPMPLVMKRQQQQQNQMQRTDSPEPYVVAPVRLDDQSVPGQDRFFPANILKMPMSDKTFTDSVDPTGTVVIDIPSDDGSGIAATTTGPAPVITTRSRQVSAGSTSGPEIKFLDQPPPQPEPRSRVNENKETMMTMSPQQHQPTVLPKRLLPGTPKRKSIATMNMSSPKSSIMSPTGSESGFATPRSASMSAAAGGNGGGSAYSSPAMAAVAAASSSTARKMAKVKVLVVDDNPVNLKVVCKMLGRLGVEPETANNGQEAVELIEKKIALLSLQLEGASAPTQADGSEAAPLPLALPLPIEHALSDANMQRQRPHLVPKAGTQQRVQHSDSSSTTSSTHGIDSGLGLLTDSDDQSIVPHLPHANSSNLPMIADFTSESLNSTSSDLTATPAAPQKPAGTGPRPRRVQLHPQEPLWQHPRPPLHHRYDRLRDARRSREVYCLGHERLHLETPSEGRVGAVLEIVYDPLLEEFSFSVVVLGE

>[MeHHK12(MS-HKII)](http://genome.jgi.doe.gov/cgi-bin/dispGeneModel?db=Morel2&id=1827482) Morel2|1827482|

MRDTVISSPAASTTLHRPLLLTFLLLFSLLQAINAQQPTTSTGLVPAPAVPTYPPSIDPGFSNTTKESYQWPNCTDTRKGNLRISLVTHSSVILPEGAKLDAKAILPLTVSITDDLADTEYDPQRMSNAIYSAINNPVDALIVSIPDYEALREPILKAKEHGIPVIAVYTGLAAAKELDILAVMSDEFEGGRTLGQQLIHQGAKDFVCIGPSLKIPTMADRCGGVLQAFLDAGISLSSDVSTRMLHVDKPRNTTLGNSIQTLRDTIVDMKTVTGVVYLTSPVFLETSFNLARALNGTREFVYASFDFSPAMMTAFTTGTLHYSISSMLYLQTLVPILLLYVQLNFGEMVNQSKILTGPKLVTPRNAKTMLMQEQWTATTFKEYALNFSVMTGSSASDDHWNALSTGARDAAQALGWEMTEYRYGSPIRTEVVAYSIDTALSYRHTQGLIVSNSHFSNVDYAVVKSLDQVPSRTSNTNKTEQLGCNNSNNNNNINTPGTVDCKALIPWNYTTNKALPVPIVGIGSPVTNLTSFQHLSWVGETGYLAGNEYADAILANGRRRPICVVSSDEPEQQMLMCQGLYDRMLAILGPNSLPAFNTFCVRLDMTEFLQSEQKFFELSKVYPYDSLHTTSTSLYGYVRRYSSAINNISITTTGRSAYALADFVEGKVANLWSQQSYLNGFMSVFQLAFSTVVQDSTWSFFETGPARVTYVCAKGQMFSLESDTTSLFCRQQNGAHVGRPYCHPCPAQHFSSTYNSQNCTACPDGTFTNHTGSTFCWSCDEEGQSVPACQKYFLSKQQQQQSNDNALAIFLPIGLVLFGLTATAVFMYCFQKRDRNRKILDDSWQLSYSKLMGQEPDPSQDDDNDSYGNGRDGGNTESNMEKGFFSSSKKKPAYGARPTGRFHRSHSTFAVGVNGIQPMDASGNAIGVYRNLPVFIRRIGGSKVNLTRKLRIEIMDVMELRHPKLVELVGVCLQPPDICIVYEHCSKGTLTEVLANPDLNFNWLFKLSFMSDISRGMEFLQNSKIQCHGDLRSSNCLVTSRWEVKVGGYGLLELMETQRTRYGRSTTTVSTPAVTVTAVQAENGAPAADGTEGQRNSVMRASTDSRALSIGSVVPPRPLSAIEEDENEKDGCPYLIASTAAEIHAGLWVAPENMIHRGRVYHKVATKSGDVFSAGIVFNEIMTRTSPYERQLQDMDPVDGPSQLLDLIKYDGLRPDPFVDNERDEGIAHLNNLIRNCLQSEPLMRPSFANIQHRLRLISPDGDMIGGMAALLEKYANDMEELVRTRTMHLQTRTAELEEERLRTDALLIDLKQSKNQAEAAATAKSNFLANMSHEIRTPMNAVIGMSRILLESDLSPDLMDCAETIESSGNQLMAVIDDILDFSKIESGNLKLAPEKLDLPHLLESVCNLVLMQAATKGLGLTFVIHPDTPIEVLGDLVRIRQILLNLLSNAIKFTEKGNIVVKLEPKPRMARSFHKTHYETETSESLPSSPEHAVAAGTHETSGLLVNVEHACSDSSLDLADLGAQLRSGSGSDTGYSTSSSAPAPVPESATVNWSSCRSQESPGDSHSSSSSSSSDENQVDLLWSVADQGVGIPAQRMHKLFKSFSQADDSVTRNFGGTGLGLAISKRLVEFMDGEMWAESEEGVGSTFYFTTLLSSPKSSQTVSQQLNLAFFKDRTLLIIDDRKVTRTSWIHQSSTWGFHKTLVFNIQKGLDYLKQHRNEVDVVLIDVDRPQAKVNPGLAILQQIRNLPAQGQECDIGSSGLSPPSPAPSSSPLPEKPIPCVLVSYHRRNHPDLSLYSSKIATHGCPSPISPISPMSMAPSSSASGRGGPVKERKSSKGSGSSDNLTAATDSSDSFSSHSNSHVGCMPNTMNSGMLVTKPWNSRKDRSSCFNTSPTMSSSSGANGAGTLPKCGSPTSPHHPILSGGLTTATYQSDQEDASVGHLIKPVKQSKLLPMFHGLMTGAWPLACSAVPDNDVRSDQRKKQMESLECLLVDDNPVNQKVISKMLSRIGIVPELAANGLEAVEKCRARAEAVAAAATAEGGGDGSSLTSNTTAGGGGAKKTKQYDIVFMDIWMPVISGHEATKEIRATVPGVTATSPLIVAMTACVMPGDQQKCIDSGMNRYLSKPIRKEELSKTLEEWLDERAKAEEELKLLNQRKLIQKKKREILQKRSCLAVLTTGVDEFGQQVEVGLSSATVLSAEDDDEDDEDEGEDNNDSDLLGQSDEGLDGEHEEDLVDSNDGEVFPCCVQANNNNVETQLHSLENNRIAAARQKRRHQRIRLSADPTLMSDSEGVLGCCDGGGGGLKIISVGADEVRAAKRERKQRGSRSRGASVSIQDPLTDGVVLIQADRSSGGDEFDDDEFEDEESDMDAEDGLQQPQTVFAEGVSIERLLSQMTIDSFHTANTHTHNTHTADAASSQRSSLLSNSSSLRTVRGV

>[MeHHK13](http://genome.jgi.doe.gov/cgi-bin/dispGeneModel?db=Morel2&id=1836877) Morel2|1836877|

MDPDPVLTTTSSSIDPVSPDMAPAMTTSTTTATSAAMTTQSTSTTTTCLEPGDDAEPHFTHSSNNNNPNSKSDKNNSIVAAITPSLTLSSTSAKTSESPMDSTINTTAAAAAAAKESATVAPSIANPSTVSPRTSTSSCSSSSSSAFFSNSSPSSTITHRSVSPPGSKPSLASKRPAAQTMSLSSSSSPSSMSDLLRRESSSSSTTFTTSRANSTSYASPKSSDPMQRHHYISFSSFLLWLKEHPASSKVGSENLARFLLVKSVSLIAACLGCFYGLVFDRQPSLSACSLLLFFLPAVQGFTGATRICGLILSLALASVFLNFQTRSMPLVGPSLETTLLLCLPMVTGMLVGRRVGLATCIVVICYSIEEYRSKTLNTNLSEDEASRIWAGFGAYWISLLFMGALTCLYQWCVEVCVRDANLFKDIAVANAKSKDGVVSSVSHELRTPLAALIGWTELLMSDQTLSSSAQSTVSMLHSSTLSLLTILNALLDVSKVSAAKMTNCSQNFNLHDLVLDTARMMTGLSGTRTVELLVDFSAQVPELVRADPGIIKQVLGNLISNAIKFTDVGYVNVGVSLKDEDDDTVTVEFVVQDTGKGIADEQKDKLFCEFSQVEGGNCKNHEAGTGLGLFLVKNLVEVMNGTVFVDSKLGVGSTFGFEIKMEKQYLAFRTPGYDHVVTVSSAFPTAGQDMNLLSPMIPVTPGLARSTTPIPVNDPLANCRMTPVSTPLAAQSEAKDYFAIQPAEITRANANNITLRTRDNNFLSNRQYYLHSQCGFFEEFLWSTSTERWKAQAVRKLSFNECTQGWQTDFNQEDSMTRFGDVFLIDLSPTTNRKTAAVLTDQDHARVFLAKLSHLLVDRAKGRIGTARQASIVLFYPFGHPPSLAQEPLLSLGQYYQISLCRKPVSERALGAIIKKQVVTIQAAVAECVSTPTTPACPSSRSSSDSTCRHLSSETIDSSTSGSSSSSSDEGSSMTRQSAGISATTGALQKNKPVREASSTSKNPSPSATQASHVRFAKRNGSVPRLALNGDAVAPPASSPSPSSSIEKALAKKSLLKRPLNSVQQQTRPSLASQQSIQGLNAAGKRVLIVDDNSFNRNLLFHQLTKLGVSKIDQAGSGQEAVDTFIPGTHALVLMDLKMPTMGGFEATALIRKKEVAYWGPVPVQRSTKNWTDASKLETDPWSPERFVAAPEIPERRSNCSGSGEGEKKNADKAQSEEGKSATVIAVTADWTAEIGEDREKAVTGGFDDVMVKPISLPSLSVLLERYMDYRN

>[UrHHK1](http://genome.jgi.doe.gov/cgi-bin/dispGeneModel?db=Umbra1&id=243181)(III) Umbra1|243181|

MALVQVLQHVCSVLSNMEVQTYEFQPFDIPHLTDEENEMVHLTNSLLDRLARKQASQDRFENMKETEPPSKKIRFYESPTATAIVLTPEEMRYQDSQGRASWISNNGAGYFALPTSDDTSSMVSPPELIMSPPASFCCTQCIQQAAAVATEIRNGRLNCRISCDNHGASSQSQALKVAVNAMSDHLERIILEIIRVVREAAVEGKLGGQAIVDKDDAKGVWNQMITNLNVMARNHSEQVRDIAEVSTAVALGDLSKQITVEVTGETLLLKNTINTMVNQLNLFASEVTRVAHMVGTEGTLGVQAKVQGIGGTWKLLTDNVNTMAANLTADVRDIATVCKAVARGDLTKKVTVEVKGEILELKNTINTMVDQLQTFATEVTRVSLEVGTEGKLGGQAVVKNVGGTWKDLTDNVNLMASNLTNQVRDIAMVCKSVARGDLSQKVTVNVQGEILELKNTMNTMVEQLRMFAAEVTRVANEVGTEGKLGGQAVVNNVGGTWQDLTVSVNTMAANLTAQVRDIANVSKAVARGDLTKKVTVNVEGEILDLKETINTMVDQLQTFATEVTRVSLEVGTEGKLGGQAVVKDVDGIWKDLTNNVNIMAFNLTTQVRSIAEVTKAVANGDLSKTISVDVGGEISDLKTTVNIMVEKLRMFAAEVTRVSKEVGVEGKLGGQAFVPSVGGTWKDLTDNVNIMASNLTTQVRSIAQVTKAVADGDLSKKIEVETRGEILDLKNTVNNMVDQLNVFSAEVTRVAKEVGTEGKLGGQAVVPSVGGTWKDLTDNVNMMAGNLTTQVRSIAQITAAAAENDFSRLITVEASGEMDSLKTKINQMVYSLRDAIQKNTLAREAAELANRSKSEFLANMSHEIRTPMNGIIGMTTLTLETELSRQQRENLMIVSSLALNLLTIIDDILDISKIEAGRMTIESIPFNLRSTIFSILKTLSVKASSKKLDMTFDVDSDVPDHLLGDPLRTKQVVINLVGNAVKFTSEGSVDVRVCVVGDAGITDGRITLQFCVTDTGIGIPEDKLSLIFETFCQADGGTTRRFGGTGLGLTISRRLVELMGGRLWVESTYGKGSKFYFTLPVAVGSNQFESLEQRLAPFQGRQVLFIDSLGLPESNTFMEQIQQLKLRPVRKTNVQAVAKHTIPGGTGGPILRETGDPIDMIVLDELSLATKVRELSHIRYTPIVVVAKTLPPLNIKMSIDLSIASYVNAPATLLDISTAMLVALESNAALQGDSSTSVPLTILLAEDNIVNQKLATRILEKFGHKCEIVSNGKLAVEAFQNKRFDLILMDVQMPIMGGFEATQKIREIEQNAATGERIPIIALTAHAMIGDREKCLSIGMDEYVTKVNIHISEGGKNDSKLTPLYIAPQVP

>[UrHHK2](http://genome.jgi.doe.gov/cgi-bin/dispGeneModel?db=Umbra1&id=242096)(III) Umbra1|242096|

MKRSSSPHSSSSHPTSEHQTRNSNSKRQKINSPSTTNEQDSMGTVVLPYINDLLQNFEQGHYDQCLKGSPPAFDQLSIDDKQSISAIESTIKSLVERHVRLETENLRYQVALRQQQQLLSSTHPSEAQDASEPATKPERNGSALLPVTPEDGIWSMKDQNVVDKVKTKSSVFPPLSMTSYFSSNNIPTPPFTTSSSPNNYSLTSPPFTPSPATHHFADNDLFCYECLIQATKVSTAVHQGDLKQRITCTHERHTSLPADSPFNLLKNSMNVMADHVEHIVQAVNQVARDTHKEGKLGLRAEVDVDATDGVWRELVVNLNSMTQSNQEQVRDIAEVSTAVANGDLSKKITVDVKGETLLLKNTINTMVNQLNLFASEVTRVAREVGTDGKLGAQAKVAGVDGTWKLLTDNVNTMANNLTYQVRDIAMVSKAVARGDLTKKITVEVKGEVLELKNTINTMVDELSTFATEVTRLSVDVGTEGKLGGQAIVKDMGGTWKDLTENVNLMASNLTNQVRDIATVCKAVASGDLGKKVTVNVRGEMLELKDTMNTMVDQLLMFAAEVTRVSLDVGTEGRLGGQAHVKDVAGIWKDLTDNVNTMAANLTTQVRSIAEVTKAVANGDLSKKIDVETRGEILDLKNTVNNMVDQLNVFSAEVTRVAKEVGTDGKLGGQAVVPSVGGTWKDLTENVNLMAGNLTTQVRSIAEVTKAVANGDLSKKIDVETRGEILDLKNTVNNMVDQLNVFSAEVTRVAKEVGTHGKLGGQAVVPSVGGTWKDLTENVNLMAGNLTTQVRSIAEVTKAVANGDLSKKIDVETRGEILDLKNTVNNMVDQLNVFSAEVTRVAKEVGTDGKLGGQAVVPSVGGTWKDLTDNVNMMAGNLTTQVRSIAEVTKAVANGDLSKKINVNVSGEISDLKDIVNNMVDQLRVFAAEVTRVAKEVGTDGKLGGQAVVAGLGGTWKDLTDNVNMMAGNLTTQVRSIAQVTNAVANGDLSKKINVDAYGEILDLKITVNNMVDQLNVFSAEVTRVAKEVGTEGKLGGQAIVPSVGGTWKDLTDNVNMMAGNLTTQVRSIAQVTKAVANGDLSKKIEVETRGEILDLKNTVNDMVDQLNVFSAEVTRVAREVGTEGKLGGQAVVAGVGGTWLDLTENVNMMAGNLTTQVRSIAQITKAVAGGDLSKKIEVETRGEILDLKNTVNNMVDQLNVFSAEVTRVAKEVGTEGKLGGQAIVPNVGGTWKDLTDNVNMMAGNLTTQVRSIAEVTKAVAEGDLSKKINVNVSGEISDLKDIVNNMVDQLRVFAAEVTRVAKEVGTEGKLGGQAVVAGVGGTWKHLTDNVNLMAGNLTTQVRSIAQVTKAVANGDLSKKINVDVRGEIADLKTTVNDMVDQLTIFSAEVTRVAKEVGTDGKLGGQAVVAGVGGTWKDLTENVNLMAGNLTTQVRSIAEVTKAVASGDLSKKIDVETRGEILDLKNTVNNMVDQLNVFSAEVTRVAREVGADGKLGGQAVVPSVGGTWKDLTDNVNMMAGNLTSQVRSIAAVTNAVANGDLSKKVNVDVRGEIADLKNTVNNMVDQLRVFASEVTRVAKEVGTEGRLGGQALVQGVGGTWKDLTDNVNTMAANLTTQVRSIAQVTKAVASGDLSKKIEVETSGEILDLKNTINNMVDQLNVFSAEVTRVAREVGTEGKLGGQAYVNGVAGTWQDLTENVNMMAGNLTTQVRSIAQVTKAVASGDLSKKIEVETRGEILDLKNTVNNMVDQLNVFSAEVTRVAREVGTEGKLGVQAQVEDVEGTWKEITSNVNTMAANLTSQVRAFAQISAAATENDFSRFITVSASGEMDSLKTKINQMVFSLRDAIQKNTQAREAAELANRSKSEFLANMSHEIRTPMNGIIGMTSLTLETELTRQQRENLMIVSSLANSLLTIIDDILDISKIEAGRMTIESIEFSLRSAVFSVLKTLAVKANQKKLDLIYDVDNAIPDQLVGDPLRLRQVITNLIGNAVKFTTEGEVVLKTKIIGTDENMVTLQFCVSDTGIGIQEDKLLIIFDTFCQADGSTTREYGGTGLGLSISRRLVQLMGGTVWVESVYGRGSEFYFTMQVMVGSTPFQVIEDKMARFQNRHILFLDSIGDTTDLQSSIEKLKLRCTRVRSIQEAANMNAARSKEAPIFDTVVVDKVAHAEKIREIVHLRYTPIVLIAPSIRRLNMKLCIDLGITGYINSPVNLPDLAHALLPALESHAALPSDLTRPSPLEILLAEDNIVNQKLAVRILEKFGHKVKIVSNGKMAVDEFGDRPYDLILMDVQMPIMGGFEATQKIREIERSAGLGQHIPIIALTAHAMIGDREKCLSAGMDEYVTKPLR

>[UrHHK3](http://genome.jgi.doe.gov/cgi-bin/dispGeneModel?db=Umbra1&id=261491)(Dual) Umbra1|261491|

MAESSRPIHSSMVPNDSAEKPSANVIPAKKKQFGLEQPELVFYAPSDNFPYKLKGEPQILDILHNTDWSKTVLGPAKDWSNILVNSIRLALVSRFAMCIWWGPDFMQMYNAKYGAFLGKRHPRAFGRDMRESWHDVWEDIEPLIYAVIDDGESVFLKEGRYWMTRFGWMEETYFTYQYSPIHDDVHDKPIGIFHVVTDETSKVFSERDMVTLRDLSVGTNAATTYSYWKRAVAKVFKVPSNCTDVPSIDLYEIDRETNQFRRIISEGCHAEQPRLQFPETVQIWEEFQSHHSDTFSADNMKIPSHKLRGSSASLVSSTTSDSIENKESVRKSLDPATVIKNRLTKAMNDCFVHNKETHLTLPVETLPIPNITVPDSEKVYLSKTGPSANAVIFPIHGSPQDYHDISLIAVLATNRFRKYDEKYRSYFQLLVSQLSGSLGTTSAIEDSERRADELTELNRSKTTFFSNISHEFRTPLSLILGPVDQVLHDPTLNPKSKEMAKMVQRNANRLLKLVNTLLQYTQLETGRMRAQYKPVNNLPETTREIASAFDTVAVRFGLQFVLDCPSWEWSEPSNRNYGPDDTTAFVDVDIWEIIILNLLSNAFKHCIKGSVRLTLRRHLRPSSSSPTAAIYRNPFITPSLASSDTVTPESVSSISSNVSSAGYYELTVADTGSGIAEEDISRIFDRFYSVRSTESRSHEGIGIGLSFIKDLVESQKGIITVDSTLGVGSTFTVKLPLGYRHLNPHAVVRSVDDNDSVRSPSNNLPVIKVDTLHKNTVNRMMPNNGKLFVAETAGWNDFDEHPQEPSQHPEETPLEPSTPSQLVTPAMESEDYVNHHADIFKPTVLVCDDNTDMRSYIRHTLISNFNVIEASNGQEALDIAVRLAASEASEMSMQGDDAVPRRRIDLVLADVMMPVMDGIELSKNLRANPFTRTLPIIMLTARAAAGDSMNGLFAGADDYLFKPFDAQELIGRVTTHSNLYRMRLEYADARNKIAVLEAANDAKSKLIALVSHELRTPLQSIIGTVELLREGAQFEEERTDLDNIHYSSHVLSALISDILDVAKIDAGNFVFEPADFNPRILVQHCCDILTEKANSKGLDLVCHIDQGIIERANQDPARIRQCISNMLSNAVKFTEKGYVAVKTYTRLSFSDGRASRRFVASELESEDSWPDKSSSKGDPNATAELVVEVEDTGIGLAQESMHEIFEPFCQANTSSTRPYEGIGLGLAITKQLVEKAGGEVGLVSELGKGSTFWIIWPISPPLASYRTPGEDSTSDDNITLTDDVNVPKDISILIVTKNQVAALAVAENLISANINGITTTTDINHAMQLVSEAKKPYDVLIADSFLESQCIPLVRKCKENKTFVLTIVSRGLLRTLSKGLKDLTDEVIARPVHRANLLDKLSKLSIEKRTRKISIADQHKSFLGKLEASLQKHIIARTDEQEVPLSPESMTSPGIGSRASSSTLVEESSSNARDTLTPRRQSSVRAPGVIKGTILVAEDNLINQKLLKKQLLKLNYESDVVGNGFEAVNMLKTGKKYIAFLCDCNMPQCDGFQATAIIRGSEESFRNIPILAISANAMAGDREKCLNGQMDDYISKPLTIPQLGSALERLLSNSKY

>[UrHHK4](http://genome.jgi.doe.gov/cgi-bin/dispGeneModel?db=Umbra1&id=252734)(VIII/Fph) Umbra1|252734|

MEPDSENLISMREDVLHVKHEGNFVPCDQEPIHIPGAIQHHGALIALSLSNLKIEYLSDNTPDLIGLPHIQPNHLFELKSFKKLLSDYQRDRFYKHIDALRDASKVGPSSFVLELDTSIFYRSKRFADILQDHRQSVEAASPASHDSDIGYYSEKTESRASFSTASSTPSGQTTGQDDYLNGLWNDLSDDDDDSDAEQSINPHKRQFYCSMHKSQRNPNLLILELEVYNRLAEVAYDKFFFGLNTLVHTFETAKNIQDLCSLAVRYVQHVTSYERVMMYQFDEDWNGVVVAETLAMDSDAESYMGIHFPHTDIPKQARDLYLLNKSRYLCDRGALTSSLLQANGKGSASRSKATRPLDLTFCHLRAMSPVHLIYLKHMGVNSSMSLAVTVYGRLWGLICCHHMFALPVTFQARTACSYLMRQLAEQYWTGSRNASQKAPPEPTATKVNDSPADTSVDSPEDEYCSSCDERDYFDKSVAERLENLHIDVNAVKRQTSRSSSVTSQRSGKFRRRPDHDRRSTEDWARMYERFVRVAGPTVCKLFGADYMLFMIQGRSIVVPNPALIPDPDALTRFVQYLQSVRLKKTVVSSCIAKDFQGMESTRALSGAIYFPLSDDGIDFAVICRQEQIMNVTWAGEPMKSGDTMTIQAYNGRQNDVLMPRTSFQKWTETIEGTSSSWSTHVNPELLLTTLSVFQESLKTWKNRMLRIDGREARYRNSQLEQAKRLAEESDKKKSLLLANVSHEVRTPLHGISGVINLLLDTDLSSEQSQMLQEANQATKTLTTIINDLLDFSKLERGEVHLEKASFNLEDAISDVTQSYAPRFKEKNLGFFVKFDPSLPKWAIGDVIKIKQILRNFVSNACKYTSKGEVSVNVSLIEVHEDGNISVKVGVTDTGIGIPIDRQQLIFEKFVQGDDSLSRNNGGIGLAVSHTLAHLMGGKVGLVSKPEIGSEFYLELPLEPVDQHPDSTVAPTNGSDTKNVRPQLVPTIPIPLAQTLPAHTKDELIANFSSPTEAISPYPVMNVSEIESILAQYEGAHRPFRVLCVEDNKLNQALICRMMQKLKYVYEVADNGQEAIDIYVRANTTKSTLQKDSFDVILMDLQMPICDGFEATRQILEYQRHTFGAAVIAAPIIAVTAQAMSGDREICLSKGMKGYVSKPIDFQVLRQLLEGLRCQKLVAETGSPSLEK

>[UrHHK5](http://genome.jgi.doe.gov/cgi-bin/dispGeneModel?db=Umbra1&id=237333)(X) Umbra1|237333|

MSHAGDSGSARSVNDTSAHSFYSDDMLQMQGYHFSTPISGSDTGESNDFAKGYRIADRKPVVAKFSSNTLKLEREYHTLRRLYQQPDGPKYIVQALERINVRNGITVIISADEGTSRFDRHRLQSEDTIKTGETRSYSLSTSTTSEFNSIDTTSTSIVIHPAPSEANTQQYDLATFLRFAIKCTDCLEFIHRNNTVHGELRLSSFFWSEEDEGKVKIASFGQGARSFESYLTSEGWRKNFSSKDGLAKLRNTLTYLSPEQTGRTNYIPDHRADIYSLGVIFFVLLACKEPFEGGPLDILNGILSRSLTPIHELRPDVPAVITEIIEKMTAKSPDCRYNSAIGIREDLKECLRRLSAGTDSYEAIQTFPLGQCDVASIFTLPSILFGRQNEIHQICSTIRRTAGFYNRKRSSRDRSTVTATATESSNLASDLNPSSASVVESISDTSSGLGANVQYERSSPSHESMAGESEMSGSTNRNYGSRKSSSLLMAITGPSGVGKSALFNAVQTTARHYGYLATAKFDHRSQVPFACIARCISQILRQIMSESSDTNVLNVVKETLEAQYVNVHKLLEWVPELSFAMSEMEDEDKPTIADIAISDNRAELHFVFVQVIRALAQHRMITLFLDDLHQADQPSLDLLFALIAAKTKILFIVSYREETVNEKITQILELDKSAVQHIHLDNLDMPALTDFICQTLHRSVQTDAEAVAPLANLIFRQTRGNPFYTCQLLRAFAGKENLIFFNWDENKWDYDTAGIEAYITSGEEGALDSDLDVSYLVARLRELPLDTQRFLRYASFIGNTFSWNAINYLMSSSFEDDNPHEEEAPTTAASLSAIHPLVKLAHTYDESHTPRKSRLSKKRSAINGLQTALQEGMIVPLDKDEFRWSHDRFSQAAMSMVTPSVGEQIHYKIAVYYLQDPECDSVIVADHLLHCADLISTCSNRPHYRDILSKAGWKQHNSGAHKCALGYVNAALQLIDDDPWKPSTYDQTLHLYTLGASLSLVVGDSSDTETYLDVIFDNVTEPMERLPAYRIKGRYYYAKGIHDDALNVLFACLGEFGVTSIKADPPRNEVDEIYYSLKNELTTIGLDEVAKLSKCNTADKVKHQALLGILEETLNVAYWLGKQTLMFYIAAKIIDISIQRGVVACTGIAFQWLGLAAVEFYKQYSYGHDLGATGLALVDRIGGNVEKGRAKHTYASFLAIWKYHVKDVIPVMESGARYSAAAGDRIYTSYGLLQTAMFTFYSGMNLWDVLQSAETAYEEVHTWAPSIDTNILIISLLRTIKALQGKTYIADAKCVFDGDDGFNDAHFVNEICLHSVNPAVPVNWYESLKMLPLVLYGHSEEAIRVGDQCHFESHNHPCQRHTRFMMFLHSLAIIDVIRRTPNMDGRNRFHYLNKVQQNQRLLNEWVQNSRINYELWYVLVEAETISLQDNFVKASKLYEKAMDLARDGHWMLEKCITHEYAGEFYLRNGIANVGVALLHKAVKAYTAHGSYGKAQQLSKKHHSVFVLVEDDEPKEVNAGVQTDVAPLLGHRDSWGELSMSNIQPAALPNPQNGLIFNDQEPTDSVTAVTAEQALATLDIVDLASILKSSQVISSEVKFDSLLKSMMTIILDNSAAECGAIIVKDEYFGICAFGTRHGGPSTFEPPKPLREEEDMISNRIVQHTLHTGESTLIPNVQNDPRFAVGSWFERAGIKSIICLPVLHKGVLAGCLYLEGSPGIFTQRHITVLTLLCQQMGISLTNAFLFKSIQKVTMTNVRMIETQKLALEEARKSKEDALRATHLREIFLANMSHEIRTPFSGFYGMISLLSETQLDAEQQDLVRTAKESCEMLLRIIDDLLNFSKLQAAKVTLDVSRIVLEDVIADVVEILIAMAARKNLTVVYFVDDDVPPCVMADGNRLRQVLMNLVGNAIKFTNQGQVVVRCSLAKQVPEEPGNVVLLFEVVDSGIGISESQLKALFKPFSQVDGSTTRLYGGTGLGLSICMQLVQLMSGKISVTSTPGVGSNFFFTIKVQTADDPAHENLELEIRDDIKGLTKALGRPRILVGSNAKSMIEMMCGFMKSFDVTSANSFPAVMAELSETSYDILILDFPITAEFAQQVRVVESGTQWQNMCIIVLHYPSGDVLHLHQDKSNLLEVSAKLVRMAIPVRRLKLLRAIADLLNLAPTKKASRSIKMSSVSQIYSPQELELFKTVRILIAEDNPVAQKLLFKQLSRLGFNVDCANNGLEAVVLWESHPETYYAVALFDHHMPVCDGVEATKRIRHTEKTSNRKRKLPIIALTADVQQTARDVCSSAGMNGYLTKPLNQNSLAELLRRYLIDEPANACLTSLSEAIES

>[UrHHK6](http://genome.jgi.doe.gov/cgi-bin/dispGeneModel?db=Umbra1&id=235482)(X) |Umbra1|235482|

MVVPLDACTSLKSSYQLDQIDYHDELAPVAIASGYRLSDKQPVIVKLSTYANKLENEYQITTRLSASKVDGQYLVKPIELISEEITALVFEDDGYKVYDGKLLSLGSTPYARYQTIRNFLKLAVQICECLGHIHSHKVVHAGIRPSSFLYQPDSKNIRKIWDFETSSRAPESIISIMDRMHMRKQFSQTMSNEQLAYLAPEQTGQTAYQVDNRTDLYSLGITFFFMLSQGLPFMDQDPRIILRNILTKPLPLTEELKATYPPIIWAIVEKLTSKYPHDRYQYVTSLHADLVRLQAVEGEAALRDINFELGVDNNSFSFEMSNKTVGRTAELHTISNAIRAAADRRNSSNQSSPPTIVSDGSRSYFANVIDEYEDLQELQEIDSDDEEATQKAEARNATVIAIHGDEGIGKTKITLCGQEYARRHGYVATASLNSLQVSPYHYVLLSLKHLLRRVLSESDEEIELFKSILDHCLRKSGFRDIRGLPAEVLDQLEDIGEFFNYRDEPLRTGFHHVATFFETKTLYHRAISSIFRALTTFRLCTIIFDDLHHTDESSLELLNTIATTNRRLVLVFTYRTDEITPALQLFAQYFNKSLVDIKLPPLEYSDIVTLIAASFNRKEFTDRADLLPMVDLIYRDSLGNAYKISQLLKSLASRNIIYYDVDEQYWDYDFEELRAATTVNQDSDEPVSDTDYIASQIESMPVEGQELLKWASLMGMNFSFQTVSQLMAADDYKHNLDGTSLELCDSDDEESSTDSMSKSSLGLQHALEHGFIHASGLDDYTFAHKRYLRAAATMIDEEQKANMRSKIAQRYATESSLDVFWVANHLLGAFRIISKLEKKAIYRKILVQAMDKANANGVQDMALKYCKSARALLAPSPWVDGEDSSYDETLHIYHNLSQLHCFFAHQADSRAAADEIIYKARNGIDRSRAYKVIYLHLYASKKYEESIKLLEISIDDLGLITVARQAQKEQVDKSYNRLEHEISAIGMDNILSIEPSQDPILHATMVNMEQLCISLFWKGEHTSLFANTITIIEASLKHGLSVASGTAFTILAPFIATRYGKYQFGCDIAQLGLQISEKYGNHSQKARTAFYYYTFMSTFNNHIEKDIAPLRAAYDNAVLSGDAMFARYIPVRIAVAMYFSGMPLDQVMKEAWDSSVKIRNWTTSTESYNLIKAVKRNILALQGKTYGTAEGIFDDDEYNDNDFIEESFPTGEQSDAVMNWHYSYKIMCLYMFGHDDFAIELAFKCFNILDSQPCHRHTRWMLFYFSLSLIRKLHQKTVADDVRVRYMEQIRVNKSLIEEWSQISPINYQMYVIALDAELASLDNQLHISQKHYEKALKLARAGGWNVEIALFYELLGEHHIRQDNTFVAEAMLQKALSAYSAHGSYGKARQLRGYHKDLELIDSLPIEVSVQTEFARVDINVERSSAKESTESFQYGETDENLMSLEIADLASILKSGQIISSEMNFDLLMKQMLEVILESSGADSGVIIVKDGTELNIVGKGSKTSGCRYLSPPERLESSIDHVLVRIALHTMQVSEPTIVTDSMTRTTFLDEYCSIKSAICTPILYKGVLIGCVFIETYQKYLSGRQVRSLQALTRQIGISVTNARLFNSLQGAMKDNAKMIERQQIALRDAKESREAAIKANRAKSNFLATISHELRTPFAGFYGMISLLTETDLDDEQLDIVETAKESCQMLLKIIDDLLDFSKLEAHKALLDLGPTSVPDVIADAIDVMASLAIQNNVNVTYSVDHQVPSTVLADAARLRQIFLNLIGNAIKFTKNGDVQVNCQLDSRDTHQDGVYATLRFEVIDTGIGIAPEQQKGLFEPFAQVDGSTTRLYGGTGLGLSICLQLIQLMSGSIGVISEGKSKGSTFWFTVQVKCVYPTQLTRSNSHLKKLSSNKILLATTHLPTAKMVQSMTPDLTITVKELTNDLFSDCHSIDLLIIDLPLIQQPGLCEELQSLIKTKKLTCETIVLYHSSVDWHRRLLGDENYWKEYTPSARITKPLRREVFTKALLEILSVPISQREVKEETKPNPSSGSRNKTFWTVKEEVWISTHNNVLIAEDNPVAQKLLVRQLQKLGFAVESVKDGEEAINIFTSSKRDHFSFAIFDHHMPKCDGPEAAARIRHIEATDNLNRMPIFALTADVRAIARKASEQNGMEDYLTKPLIMERLVAAIRNHCMHSSTATL

>[UrHHK7](http://genome.jgi.doe.gov/cgi-bin/dispGeneModel?db=Umbra1&id=260316)(X-B) |Umbra1|260316|

MKSRPSLSLAGMLPDSSKAVYQENDAVDFDIAINGYTDIQPHGLMASRLAGDARLYTAISTKLNQRVVVKECDANLYDMTKLRHEYNLLMSLPEDAPAILKPLALESSSRGLMVIFPKQEGRLTLRELYLEGLPKRNVPIAAPVMPLDKFLDMASQIASSLVCIHELSIVHRNISPGALSCDNSNRAALHNFTLASRLTTEVTSFSKSNAKILEGNLLYMSPESTGRMNRTIDTRSDFYSLGATFYHLLTGLPPFDTNDPVELIHQHIVQPPTPPHAIVPSIPTPVSDVILKMMSKTPEDRYQSAKGLKRDIEILIARNRNNDWHNFVAGEVDRNSQFILPQTLFGRSKERGVILSAFEHAKEAGGSHLLIVKGYSGVGKTSLVNEIQRPVIKAKSYIAASKFDQFGRDIPFVSMIQAFRDLIRQLLSEPLSELENWRKSIQNAVGNNGRVITDVMPDVETIIGPQPAIPLLGPTESEARFTNIFQRFVSVFGRPNRPLVLFLDDLQWSSKTELNLIRKILCSPEKQHLLLIGAFRSNEVLPGDLLAVILDQLEEDPTVNMTTVALGPLDHESVRCIVSTTLIGHVDPIKHTLIERHQSKQPTMHHIQELTTLIYDKTEGNPFFVLQLLKSFYTSGDISFDYGTNAWQWDIKQLYTKEISPNVIDLLINVMSQLPAPTRNVLLLASCIGNRFTSDLLSIVNQKDLSATVMDLWDSLAAKLVIPLDSNYKVPMAFFEEHLESIDQHLQLAENAVMDSKDEMFHHSPSMMSSIDESVRSKHILYRLTLDSETTRISKEPIVIHFKFLHDRVQQAAYSLIPEKDRHKTHLQIGRLMLQYADNQAQTEESTMSDEELFNRSRQGFFQLTSYLDRNIFNITNQLNAGSDLLKKLPNNGDEIRRLISLNLRAGLIAQQATAYDASVKYFRHAIDLLDDSSWETNYSTTYFVHFGLADALYHATAFKEALHWFTTALNQCRFAKDKAQLYHGLLKCHMGDGQTSESIDCVLEGLQILGYELPSTPESMESYCATISHRIESMNVEEIRSMANLTLTKDVLHLATIRILVSSIPPVYFSRPELLPYIILTGLNATLDNNVVPEVTYIYTLYGLLTIGTAMNKFTTPEELANVIVIAYEWGKLAVTSLEKYERTLVECATLKVYASHVQCWNEPLANTYATFEQSIEEGISTMNGEYVGYGCVEQCMYMFFASEALEGISDRCARYKVIMERFRQEVGNAYLRVGYQTILNLMNRGNPNPCDLIGQVYTDSYHQIVQSNNLILNAFCYDLFNLILVTTFRKKEEALKFSRSGFSSIDGAIGLLHTAMFYVFSSISFLENWSQITEQERQRVTDAIQRCKGWAINAPQFFEHKLALLQALHSEFAEDNFLCAVDLFDAAIDGAKKHGFIQITALACELAAEFYMRRGKQRIANDYLLESYYNYVRWGAEAKAKDMAMRYPSTIGKHRREKAIGSGDQSGHSLGSMLPISPATSGSRNRVSESANPFEALDNSFVNRERPTASTSGGEIVGTQMDNIPPIFEDTSPMSTNDTVSAITSLSTSQSYIFGESNTITSLDLDTVMQASLVLSEEIVLDTLLEKLMHILLQTASAERVVLLLEKQKKLFVEASACHSDKRQVTLADSLLPLEDCRETLPVSIVYYVVNTKQTVINDTNQPNSLFQADDYILAKNPKSIMCIPITHQGLLIGVLYLENTQVYNGFTQERRDILQLLSSQAAISIQKARLYKDLNDANENLTKSHEQIKEYSIDLEHKVNERTIELQEKNRRLELEIENRKKAEQEMRVAKEQAEQATQMKNLFLANMSHEIRTPFNAVIGMTHLLQDTELDTQQMDYTETIRNSSEELLNIINDILDFTKIETDKLDLECHPFSLRSCAESVMDVVSAKAASKGIEIVFWNQDGDQVNDWILGDSTRFRQIVINLLSNAVKFTDHGQVIITVKLYTKEEGLQLQNSGVAKASGAGMSSTGETSIPFPTPPTHMLHVSVSDTGIGIPPDRFFRLFKLFSQIDNSTTRIHGGTGLGLSIAERLSATMGGYMWVESKGTNQGSTFHFTIQTTPQPSQESPDVEALIKLRQYGLHCLIVDSNEVTRDVLRTLIEALGVHVHVATTFQDATALLIQFNIRVLLIDARVGAKNAANADIKVADLEGIRLLSRIRKWESENKRVAAEAIMMTPLGVRLSATAMEGRRIGTVCNKPVRRSRLITALLDAFSALVAGTKQSISDGRPRRLTPSIDAVKRPSVIPKNISNRTGLLKILVAEDNIINQKVIVQLLKRMGFTTDIANDGAEALDMMEATTYDIVFLDLNMPKKDGLTVAREACEKYPPDVRPTLVAMTANAMRGDREQCLQAGCKDYIAKPILIPELSRVLESCSKHTADEESIPPSDHRSKRQKLT

>[UrHHK8](http://genome.jgi.doe.gov/cgi-bin/dispGeneModel?db=Umbra1&id=253934)(V) Umbra1|253934|

MQPAASTEHERAGALDLSRRSVTVSSDPLVYARSTSQYHAQLYNDQQQRVSSNDHPSVKLPNLPQFNQVVLSLEPPNLYKTALKLSSEIDLQNWWYATIDILSQSSFHASRVCLSLPQDPSDPYHSPWGVKAVYSKSNSTQYQTSDAENDPSTEHQPSDDYFSKSSLGMPDFTSDGSSDKPKVPTLPLCFESLQPFDRDDEPLVDKNSVDRIIRRGHTVVLSREYRQSPSSMHSSNPSELLHEQDGSGMFKRSLMERLDSYHHHHQVHHQQHAPPGTQFSQQPPPPPDRKDLLRRRSSNAISMAQTPPPRGTPCLNLCDHPRNEKDECRFEEAEALPHSSSSGDITTNNSAPPGEVRELYYDEYEQHQPSPWSQSPAPSPIMMDPNVNPFFQSNIGIDDDAFNPTSPESYSTTSIPYPVPIGNIHSIVHIPIYHHTGRNTGKLPTGAPFAILSFLSTVVPYPQILISCIESIVPFIATSLSTCLAHQHLQRQLFYYRHQDSGNHNPFSTSDTQSPSKHEEISPETASSNPISPAQSRQHPYNATTDTDSATPTPTAKGVFEDPGRGPFSAGGIAMLQQMETSVPEDDMAQEEAPDFNLNYDKHTQAIVQLTESSSSSSFPQGQDTATHPTSNRFTSSTAVSTPDSSGSASPLSSAMRHGWDAISPTTGLPIVSSIPPSSVYRWRTLSSGSSAYDSEAILSPGTSEEIAKRQGGRRIILPARTNIVHPTMDDDALASDEADEPPEKPKPKVKRRHPPPSTQPTLSGSEDTEGGERKKVHQFFEANGDTDVVPSDLPMDETRMITPKSRLLRLIIDGIPIHVFTCSTRTGRTTWVNSRVMQYSGQPMKHHLGSHWLSHMHADDRHACRQQWKDAFERGVGFAGQYRLKRFDGEYRYFLWRTVPLRDVKGNIINWFGTCTDIHDERVAKELSIRQLEIEHNERKYRLLAETIPQLVFTFSPEFGITYANQKWEDYSGKPLDCAKGLAFMAQVHSEDRHKLRLPEVGEDESVSWQEEIRLLGKDGEYRWFLIKCNSVHQDEIPDARWFGTCTDINDQKQFEQKLKEAHDAAQKSTESKTRFLSNMSHEIRTPLIGITGMVNFMLDSDLTPEQLDYAHTIQQSAESLLVVINDILDLSKVEAGMMKLEMEPFSLLGMVEDANELLSTLAIQKGLEMSFWVDPDVPDVVIGDRIRLRQVLLNLIGNAIKFTTSGEVYTKCTVQKPITSDSEIMLMFEVVDTGSGFDADGEAVMFKPFSQVDASSTRKHGGSGLGLVISRQLIELHGGSVTCTSRKGEGSTFYFTVKFSIPHPATLPRPQTPQEETIKSPFFRASAYGVHSHGMTTPLQQQVISSALETSGFGTSSPLEHSIKHTQLGVVTSAARVQEALLYKTAPSGGLLPTGAGQANPDVTTDFAGAVKKRDLSCKISRELQLPKAFRTSTDDIEAPSQPTLSVPSHIQSTPSAPIAARALIVSEWIYSRDVAIKHIETLLKEHYKTIDTLDVCHSHVEALQILTNPDKLPYSWIVINLSMQQQILPLIRHIATSPAHKDAITIVLTTHMQRSTIFEGASDEQDRNLFDNCEFVFKPLKRSKIQQLFPSSDVSSSTSGEDPAGSNATVGRKRSLRNRHLPPAQQMVAGQHEVFQRMLAEVGNRGHRVLLVEDNLVNQKVMVRYLVRVGLEVDVVSDGSQCVDAVLSRPHNYYALILCDLFMPVKDGYEATKEIREWEAKTLHTNAYPIPIVALSANVMSDVAEKCKQVGFSNYISKPVNFTTLSNVVRGYIQDHEKHQTTTV

>[UrHHK9](http://genome.jgi.doe.gov/cgi-bin/dispGeneModel?db=Umbra1&id=240602)(MS-HKI) |Umbra1|240602|

MKSASHAIQHQDNPLTIPQTKAPLYQDLSPQIQQLYTNTFSDVGLRFLHLLASEITSITSCRSVFVHELLSYEEYKESDLYRKENTFSEEDKLMVIRALHVNPKDAVTLSDPLKTVLRLEGTPHQHAIQHGQQIILQDLAISYPAYQGFQSMVAVGLPITPSEDFPISTPPTPPKECIGVMGILHDKPITSQDAHHILKLLEAVKLRTGRELERIREEERLMSTREAAKQDAENKIKFLADMSHEIRTPMNAVIALTDLLLQERETLSEEQTEHLEVIQTSGHHLLTVINDILDISKLNHDPKLKLEKRKFSLRKCVKDTLNMARHQATTNLQNKIVHLIECPADVEDSSSLKQVLSRVSKESANSPLPRVMKGKTLLPLVWKIERDVPDILIGDSMRLTQIVLNLCSNAVKFTKEGHIEVNIKRYVPNAVQTNTTTISSITGKPVYEAKIENIRSRAVRESTAQKAKQASAANPSQEGTDALIGVDQTVILEISVRDTGIGIPSDRLPKLFKSFSQIDISTARRYGGTGLGLAISSTLVNHMGGGLWVESEEGVGSRFALTIPIAVAAHQPDTPGNRSSDGWTPLTSPPSPCSTSSEGTSGTQSLNTDGSQEFTSPASGCLSPSHNAAHAQGYFSQPVAHTTRPASPAVSLAQTLQSSTGHHQSAIPPLTWPVPQPPPSSFHLGIQSNTKHPSVAINVDSKPLSSASRGQSASTKSDPTMAVGVPSTGPPGYSPGLKPQTGVTTGSGSNSLLTTTATSGGQVASHSRSSRASVAKQHHHQRRGGNTNEENLAISFPIKILLAEDNVLNQKIAISILKRLGYTGVEIANNGREVLDAMRRSRFDLIFMDLYMPEMDGLEVTKSIISARHSPNTELNNVHDVYIIALTASASMQDRQICIEAGMNDFISKPFTMLEMKQSLKKCMYRQRKKRRKQQQARQDATNNHADKQSDGEEEEEDDDEEDVATNNNDPMLGVEGASYQGLIRGRT

>[UrHHK10](http://genome.jgi.doe.gov/cgi-bin/dispGeneModel?db=Umbra1&id=280328)(CKR) Umbra1|280328|

MRKPSFGGKPPILELPTFEGTPREATFMYTPSDQSTRDVEGLSVFESDGQIDRDSVYTESDKGEAKKEGYRLFGIIPYYISVACLALFVSLLALTIGLVLYYYLHGEDYQKFRTQIHTECSNKATAIEKQISGIAAAFGGVSFWLDMSPESSRTQSGFDLAVRLATANSMNTNAVEVIAYAPLVNDSDRAAWESQYQTTIKQSQPDNSLITSEQKPYYFPVQWQSPVGEFILGLDVYSSPVRSDALNQTLLNKTPTLTPFFSLVIANVTGFCVYYPVYQNYSMNDDALSAIVAVCFGADIALNEALEGLQDYVTHVRVMDHFGTVGYETTGSQASGYNETMEFSVMNETWVISCYENYTAQPFAAVILSIILLIGFLATIAIMVCSRRYHRTSNDAIIHKFRYNRSKRRAQAILQSIPDPLLVLNGDGRVVDCNQQALHVFGVDHKKRLLGCHIKHLFVSSFTSALIRDNVIQPGLHEVRVKRHQTGEYFIAEANFSYVEDINDANDEKPFMTQVVLMRDVSAKKEAALQLQNAKQQAEDTNQQKSQFLAFVCHELRNPLHVISGMSELLNNMVVQPDKQEYVRNIITSSNHMQSIVNDVLDIAQLSENELRLNYQPFNLKQLLEDEGKFMMRSIQEQEGDVKGVVNISYSVPEYVNSDSTRITQLIKNLVDNAIKFTQQGVVTLEVSASDKKNPDSTIENVLFLISDTGIGIPVEHQDEIFQPFSKANISMGNHFGSSGIGLSIVHHLVDLMGGSIEVESEVNVGTTFKFTLPLALATEDDFLAFQSANDSTSSTALSPVSNFGPSAIKPTLLLERPQPKSSWSIASNASNDQSIGRSDEQDSLLVNRGSETNLISSASTDARLSSCLSLSGLSNGGTSSGEHDSDAVSTPNSVHEGGSYSTTRKDYFGALDTSELGTIEEKPVCLVAEDNELCQKIATKMLGKEYVVEIAANGQIAVDTVMAAPDRFNIIIMDIIMPEMDGIQATIELRKNGIKIPIIAVTANAAEADKKEAMRHGFNGYITKPYKRNLLTAMQRALSAAKQESSSS

>[UrHHK11](http://genome.jgi.doe.gov/cgi-bin/dispGeneModel?db=Umbra1&id=229472) Umbra1|229472|

MYGKSRVQRNDNNDMDSPSTSTPNLQWFIMPETVLMTDANLNIETTITPNTKSFNQSLRGQPLLKLVADDEVQVIREIVKKTQIGDGDPVVESITRNRFMNDITYKISPVLDIAGNIVQYLITRTISSAPKESSPPRFMERKHSGSKDKVANDIEVHHKSSIGNDKILSKFIKVVENADPSVAAAIWLADRNNASTLHLVAFPKIRELIGVKISINKKHLNINKHEITSIPLKDSHFQTPELVTKLEDHNLFTESTVPLHSTSHSLVGLLVFYGPKSHQPSFSLKELVNDATSLVESVIALEYEHQTSRHLIEERMSTSLNASAGSNATLIVDSHGRLQFISTIVRDMLGLSKTANVDLHNLENFVDEKDRHRVTSAVTKSKQNPQITRYVDCVEFHRRIPSQEENSQESDYEADGFYLDLKIQGLWDDPSVNGCLIRARDISPKAISDRQTLESQKSLAMTNNSLNEGLIRTNSEGIIEYVNLAFIKLIGMDEWMLTSRTRPGSGGSGSPTAELLSSSSSASDSTSTDDDDQNVSLMTTLIGTPIDDLLQLIQVVIQERSPSLSASDTAGSATVTDNSTAPTRNHQQKSIINSRSASPLVTESASDTGTDTKLDSSNSTSTDDDMFNDGFSYKTLLVDMKSMIKRTVITKDTMTLADNKFHNACHEDVYSDVLTQDMVDIVNFQLGEPDLGRSIGYLIRRGGQTAVVAVEVTVNPLQRISYKRSKARAHSKNTDGVVLVFRNMTEVMRTQSANRQLADKSRWISVITHEMRSLVNGIIGMLDLVKTTQLSKEQTGLVNCMEVSANALICLVSNTLDHARLEAGKVSLEYLPFNMQERLRIVVDLMNMMADAKDVKFSADIDEDVPETMYSDFNRLFQILLNLTSNAIKFSSHKRKQSKQGQVKLIVKKVDEEYQAMSPLAVNTSDPMKASKFSSMLNADYESPKTTPQSPSHKSPPLPTEPNRRLVQLLFIIEDNGIGISPRDKEKLFQEFGQAEVSISRRYGGSGMGLFICKQLVRLFNGTIGCESTLGEGSQFWFTAWFQSSEVKSAAGSDSNNYFSMEQAMLVNNNYDNETSSSNQEESTESGSDTTTNDVSSEEASDSNQDTPHPRHSKKSVRRKEDVRILIAEDDRINQQVLQKFCQGLGYPNVTVVGDGRSAVKKCNKRHFDIVLMDKSMPIMGGMDAVQAIRDGQAGDKDLHIILISGDTNIDYARYRDLNINGHLVKPIRMPDLKRELDRYSASVLHSTVPK

>[UrHHK12](http://genome.jgi.doe.gov/cgi-bin/dispGeneModel?db=Umbra1&id=255834) Umbra1|119427|

MDDNPHLQFSTSPLLSPSQSTNGDMSEESSSVSWSEVSSNDFSNISSYDESSNEMLNLQASAPVASEHGHIVQFYYDEEYLYNSIISFVAPGLKAGGGALIIATKAHLAIIENKLRERNVNVEKKKDAGRLLFFDAYDILKALQRSDGTISGDKFEELIEGSIDQLKANSNGIIYAFGEVVNILSQRKQYHAALVLESKWNDFMKRRRLQLLCGYALNSFSDASDVDAMASICQAHAHINTAEHNPGDYRIDYKADSNALRNHQLALLKLQQRFKSVEVELERRKVAEAALHKSIRMISETTESVLSREKDRYQNVLSSLPVGVYGITFGDDDDFYINKRFSQLVGRSENEIREHGWIDVIHPDDRKAVETNWPFCSFHNTEEFKFLANCHEYRLIHSNGDIIWVKADTTPTTAEDGHILGYLHTLMDITELKNTERGRLEAKQAAEEHQRHRAEEAEHHKERQDQWIDSLCHELRNPLNGISGNVELLEDGIEHRRAILSKPTLEESDIQSLREQLPLDQESVLAVKNCVAQQKVITDDVLSVSKLELGKVVLKKVDFNLDTVLSDIIKMFQAEVAAKGLGIVTKFDSLPSTIRNDPQRLTQVVINLLVNAINFTDSGKITVTTRRIPSNGMENMVKISITDTGVGMNPKEKANLFQRFTQPTSKNTFHEYGGSGLGLYISKNLVKLMGGDFSVESEKGRGSVFSFTIRDDHIEDGALKSHTEKIQVSKPLDISSPSNNVSSANGLPESPTQACVNRQIRTILLVEDNKINQNMMKRLLKMKGYEVLIADNGKLALSAFETKSFDLIIMDIQMPIMDGITATKEIRKLEKENAAAPIPIVGLSGNAREFHAVNALKSGLNRYMTKPVNKDELYAVIEQFEQASICQ

>[CcHHK1](http://genome.jgi.doe.gov/cgi-bin/dispGeneModel?db=Conco1&id=38317)(III) Conco1|38317|

MASNLTAQVRDIATVCTAVACGDLSQKVTVSVQGEILELKVTINTMVDQLRTFAAEVTRVAREVGTEGKLGAQAEVEGVGGTWKILTDSVNTMASNLTAQVRDIANVSKAVATGDLSKKITVNVKGEILDLKNTINTMVDQLRTFATEVTRVSLEVGTEGKLGGQAVVKDVGGTWKDLTDNVNLMAANLTSQVRSIAEVTTAVADGDLSKKITVDVKGEIHLLKTTVNSMVDRLRTFAAEVTRVAREVGTEGKLGVQAHVKDVGGTWKELTDNVNTMAANLTAQVRDIANVSKAVARGDLSRKITVDVKGEILELKNTINTMVDQLQTFATEVTRVSLEVGTEGKLGGQAVVKDVGGTWKDLTDNVNIMAANLTGQVRSIAAVTTAVACGDLSRKITVDVKGEILELKNTVNSMVDQLRTFAAEVTRVARKVGTEGKLGVQAHVKDVGGTWKELTDNVNTMASNLTLQVRDIANVCKAVACGDLSKKITVSVEGEILDLKNTINTMVDQLRMFASEVTRVAREVGTEGRLGVQAQVTDVGGTWKEITYNVNTMAANLTSQVRAFAQISAAATDGDFTRFITVEASGEMDSLKTKINQMVYNLRESIQKNTMAREAAEMANRAKSEFLANMSHEIRTPMNGIIGMTALTLETELTRQQRENLMIVSTMANSLLTIIDDILDISKIEANKLVMEQIPFSLRSSVFSVLKTLAVKANQKKLDLIYNFEGSIPDQLIGDPLRLRQVITNLIGNAVKFTTKGRVILTAQTKELIEDEVVLVFCVSDTGIGIDAEKIDLIFDTFQQADGSTTRKYGGTGLGLSISKRLVVLMGGELWVHSVFGEGSQFYFTVKCKISNIELEQAMTRLAPYATHCVTILNSQKSPEMYDLTKMLETLKLQYILVETVEQAGEISSKATAKGTRPDLLICDNLDAIEKLREVPSLRYVPIVLMGHDMPDLVMKVSIELGISSYINLPASFSDLVNALHTAMENYAVLPADPSRTLRTPLHILLAEDNIVNQKLAVRILEKFGHKTTIVPNGLQAVEAVKNNHYDLVLMDVQMPIMGGCEATQHIREWEREHGHHTPIIALTAHAMIGDRKKCIESGMDDYVSKPLRFNELLAAINKCTMTQPISVAND

>[CcHHK2(III)](http://genome.jgi.doe.gov/cgi-bin/dispGeneModel?db=Conco1&id=148367) Conco1|148367|

MASNLTAQVRDIATVCTAVACGDLSQKVTVSVQGEILELKVTINTMVDQLRMFAAEVTRVAHEVGTEGKLGAQAEVEGVGGTWKILTDSVNTMASNLTGQVRDIANVSKAVATGDLSKKVTVNVKGEILDLKNTINTMVDQLRTFATEVTRVSLEVGTEGKLGGQAVVKDVAGTWKDLTDNVNLMAANLTSQVRSIAEVTTAVADGDLSKKITVDVKGEIHLLKTTVNSMVDRLRTFAAEVTRVAFQVGTEGKLGVQAHVKDVGGTWKELTDNVNTMAANLTAQVRDIADVSKAVARGDLSKKIIVNVKGEMLDLKNTINTMVDQLQTFATEVTRVSLEVGTEGKLGGQAVVKDVGGTWKDLTDNVNIMAANLTGQVRSIAAVTTAVACGDLSRKITVDVKGEILELKNTVNSMVDQLRMFASEVTRVAREVGTEGKLGVQAHVKDVGGTWKELTDNVNTMASNLTLQVRDIANVCKAVAFGDLSKKITVNVEGEILDLKNTINTMVDQLRTFASEVTRVAREVGTEGKLGVQAQVKDVSGTWKEITYNVNTMAANLTSQVRAFAQISAAATDGDFTRFITVEASGEMDSLKTKINQMVYTLRESIQKNTMAREAAELANRAKSEFLANMSHEIRTPMNGIIGMTALTLETELTRQQRENLMIVSTMANSLLTIIDDILDISKIEANKLVMEQIPFSLRSAVFGVLKTLSVKANQKKLDLIYDLDGTIPDQLIGDPLRLRQVITNLIGNAVKFTTKGRVVCTAQTKEIHGDKITLLFCVSDTGIGIDAGKLNLIFDTFQQADGSTTRKYGGTGLGLSISKRLVVLMGGELWVHSVFGKGSQFYFTVQCTLANTTVDQALNRLSPYSTRGVVVVDTKRSDDLSDLVHMLEILQLHYVVVHSMEEAGVIAAQGNKQVPRTDMLIVDDLEIVEKLREVSSLRYVPIILMSPNMPSLVMKISIELGISSYINQPVQFPDLVNALYSAMENYSIIPSDSSKAKKAPLNILLAEDNIVNQKLAVRILEKFGHKATIVSNGLLAVEAVKNNRYDLILMDVQMPIMGGFEATQHIREWELESGHRTPIVALTAHAMIGDREKCIASGMDDYVSKPLRFNELMTAINKSVTLNQQNSKKKEST

>[CcHHK3](http://genome.jgi.doe.gov/cgi-bin/dispGeneModel?db=Conco1&id=8639)(ETR) Conco1|8639|

MMNTSDSPSDYYVSLFTVIADILISVAYFAIPVEMWFFQRNLPGPVPHQYILILYQMFILACGVTHLTAVWAPWTQTAVAQLVVKLVCAALSVGTAIIMIKVIPMAFSLPVKAEMLARELGARIRHGHHLQATNELLVKFRKMTHNIRKTLDVSTICDTAVYELCNALTVSGVAVFFPEDGRYRCTAEYIKHKGENPSSSPLSPTLVKTHYWKTITVPSDCETLRRMDGVTCASHISHSEISSMVAKQRGVLYSSGMIMAFALPNTDQRGLVLAFNEKDQWCPSQEDFELFEDIVSQVQIALEQAYQIRQESKRNDQFNNQVQKNDELSKAKRNAEMANEIKSQLLSTLNKEFGGPIIQIKLLCQQIANTDLEAGQVKYVESILNYNQTLSDVLNEANDFVHAEAGDLTLNQYTFNLKDTFSTVIPNIENLCENKQVEFTWRIDSLVPSHWMGDNLRLRKMLLNMATNSLQFTHQGRLDMVVSLEDIPPSEWMLPNMDLKSTSSWSHYTHPFPPTLTKDSLPASSKDVQDSLIPLKLYVHIKNTGKNILDKSLDQMFRSLALGDPIINHPTNAKLSLNLTTLCQLVKLMGGSISVWTHGMEGTIFSYGLTLCMTSPKTTKS

>[CcHHK4](http://genome.jgi.doe.gov/cgi-bin/dispGeneModel?db=Conco1&id=77417) Conco1|77417|

MDLNQPITLKEAISIICSGTNTPTIVCNKELVFDCNPEAAAALKTPSGKTLADMSYTSLIQDHFSPSVFNKPPTPPRPSHPTELCYARIPRLDNNYYLAPVKVKKWIFGEEEFFTLTFASSDLLSLNLTTNQEPNLGSDPPSSCDVPSPEDHKLIPIDFKVCDIIHGTKSIMEKRYQQRLLEEFTNLANLVPVMIWACDAYDNNYYVNNLYREKLGVDYNENWECVLHPDDVAEYMTNWRLARESGKTFEGQEIRLRKNPAKVLNNEPNSYRYHLVRAEPIKDQKSGKITQWIGICTDIQDLKDAEMERKRLIVSEQTALESSKMKSRFLAVMSHEIRTPLFGIIGNTSLLIDSKLDSDQKEQLENINYSAQLLVNTEPFLFHSIFKRAENMFKAEAHKKQVSLTFPQFTNAQCKLIGDPNRILQVLTNLVSNSIKFTPEGGSINVSCEHEAFGEPSKYYFKVSVEDTGIGIPSETVPFLFSPWTQAKNNSQHMHGSGLGLSISKSLVELMGGSVGLSSQLGVGTICWFELELPIVENEQPQINSNSTGKGARSYEMIEDGISNGTGCNSCEDDLEPSQKVIKTTHSSELLIPEITLATPSGFPLGCDRSDHEHQVLIAEDNLINQKIMKKFLGKIPNIKVTMVENGLEALSSYHEHPNNHYCLILLDHMMPVMNGDLVCQLIRENNPSIPIVSVSASTLNNELENFKRVGMSDHLAKPFTSKQLESLISKWLK

>[CcHHK5](http://genome.jgi.doe.gov/cgi-bin/dispGeneModel?db=Conco1&id=77419) Conco1|77419|

MDWNRSISLQEAINLICVGNITPTIVCNKDLVLGCNPEAATALKTPNGKSLVNQSYISLIQAHFTPSVFNKPLTPPRPSHPTELCYARIPRFDDSYYLAPVRVKRWMFENQEFFTLTFASHDLLSLYMTTNHESSLNAQPSPSFDVPSFDDHNLIPIDHKVCAGTQGTHSFMEQRYQQRLLEEFTNLANLVPVMIWACDPQGNNYYVNNLYREKIGVDYNESWEGSVHPDDAAEYMTNWRVARENGILFESQEIRIRKSPAKVLPDEPNPYRYHLVRAEPVKDPVSGKITQWIGFCTDIQDLKDAEMERKRLIISEQTALQSSKMKSRFLAVMSHEIRTPLFGIIGNTCLLTESKLDSDQREHLENINYSSQLLLRVVGDILDFSKIESGKFEVSCEPFLFNSLFKRADNMFKAEAHNKGVSLTFPQLKSSSCRLMGDHNRILQVLTNFISNSIKFTPEGGFVNVRCDYEILDNPIRYHFKISVEDTGIGIPSETVPLLFSPWTQARNNSQHLHGSGLGLSISKSLVELMGGSVGLSSQLGVGTIAWFELELPLIEHDPSEFVQGIRENATRTHQTIERNSQVDSSCSSCTSESHSPRKLFKSSHSQELLTQETQNVCAIDIPLGCNRLDHEHEVLVAEDNPINQKIIKKFLGKIPNVKITMVENGLEALNAYHDHPNSHYCLILLDHMMPVMNGDLVCQLIREKNSTLPIISVSASTMNNELENFRRVGMSDHLAKPFTAKQLETLIRKWLR

>[CcHHK6](http://genome.jgi.doe.gov/cgi-bin/dispGeneModel?db=Conco1&id=77150) Conco1|77150|

MDKSSIYDISQVICNSIDSPLLVCQPTQIINCNESFSKALGYESIEKIRDLDINYLFNKHFIYSDKLEASKKYTIDKLLWVKYQSSLGDRIASVRVKQANSFGLEYYTVNFHDDLLNEQNITNNKPDNLKNSPVLQPKQFSNQSTFSSENVFDPTKYDLTQLSREEIFERFYRMIYSIPCSLFIVNADNTHCYVNEYHQMLFGSDTYESWAECIHPQDVELHLRRWKHSMKSGEPLTNSRVRIRKHPSIISEDQPNTYTLYVINIYPFWDKSGKTIINWLGVCTDIQELKDAQESSELKSKFLSMMSHEFRTPLFGIIGSTSLLMDSTLDAEQLENVKNINRSAELLLVVIQDILDFSKIESETLSLNPEQFNINNLIKDTYHMFQSLAKSKLIDFKVNYPEKNYELIGDSARLLQILVNLTSNSLKFTRQGFVNISCNLTTNYQFAKVCFCIEDTGIGIPPESIPDLFRPWSQASNAKTEYIGSGLGLCICRSLIDLMQGNINIESEVGRGTKIWFTVDLPLAYPEQIEEIEQVATKEVFDSILPGVESPAQKLSKVNPLYRSNNLADKFQCQKVQEFDTNLRRKRPILSNSIANGGGTTGTNGQPIDSPISDTLHTHFILIAEDNPVNQIIIKKYISKLSTVGFVLVSDGKLAWEEYQKHPAGYFDAILLDHLMPEMGGDEVCKLIKEKDPSQVIISVSANALPTDIEYFKQIGMDDFLEKPFTFPKFKTLLQKWLKEL

>[RbHHK1(III)](http://genome.jgi.doe.gov/cgi-bin/dispGeneModel?db=Rambr1&id=162570) Rambr1|162570|

MIDASSSHAALLSEIESLLDAFEANEAISWDSSLTTAVKGGHSPAAILSSELSRTTEAVTPLHLDGTTNNGDAAAAAASVSDRHARVTARICTVLNRNRELKAELSRFKLAAATAHTASNTAPATAAAAAAPPSTNLHSHTNQPHRIDLQNHLNSNHSLAGVHSVNLSNHHMSVSSPALDSITECRESDASVDSVGLTSYDDSGSISTSDGNDDSGCNSSNSSSNNSSDESSLDVQSSPFSVSIDDDVDDVLSDNNIPSLNIDRRGISPDASLFAPQDSPMTGASILATMPSSLGRTHVNSGVSNGLFDGLATLGSAAGSLHQRTPSAVSCSSATGGNDALHFAMSEPYTLSVAEYECLVRCINAIGDIIKGNLSVRVQPPSALTPSLATPSGTTTTTTTAQILSDLGIVSRTASVASGQAGNNPTRRNSRASSAADTPLPPAQSPVEALCTSLNDMVSRLDTFTNSVTKLTNDLGVEGKLGVRVPSQPKMQGRWMTFIDAFNAMSSSHTLQIRDISDVCAAVARGDLSRRVIVPVRGEKLVLKNTINTMVDQLISFSYEVNRVAHEVGTEGTLGGQVHVKEVGGTWKDICDNVNRMAYNVTAQVRDIANVCGAVSRGDLTKEVTVAVRGEVLWLKNTINDMVSKLAIFASEVTRVAKEVGTEGILGGQATVDDVEGTWKDLTDSVNLMASNLTNQVRNISSVTKAVAQGDLSKKVEVTVKGEVLDLKNTINSMVDQLQTFASEVTRVAKEVGTEGILGGQANVDSVDGTWKDLTDSVNLMATNLTNQVRDIATVTTAVARGDLSKKVEVSVKGEVLELKNTINSMVEQLQTFATEVTRVAKEVGTEGILGGQATVENVDGTWKDLTDSVNLMATNLTNQVRDIATVTTAVAKGDLSRKVEVVVRGEVLELKNTINSMVDQLQTFASEVIRVAKDVGTKGILGGQAEVHNVAGTWRDLTDNVNLMASNLTKQVRDIANVTKAVAAGDLSLMVTVDVQGEIHDLKMTVNSMVSQLNTFASEVIRVAKEVGTEGKLGGQATVEGVDGTWKALTDNVNLMALNLTNQVRDIATVTTAVAKGDLSRKVTVDVKGEIHDLKMTVNSMVSQLQTFASEVSRVAYEVGTEGRLGGQATVEGVDGTWKDLTDNVNQMASNLTNQVRNISSVTKAVARGDLSKKVEVAVKGEVLELKNTINSMVEQLQTFASEVTRVAKEVGTDGILGGQATVDNVDGTWKDLTDNVNQMATNLTNQVRDIATVTTAVARGDLSRKVEVAVRGEVLELKNTINSMVDQLQTFASEVSRVAYEVGTEGRLGGQATVEGVDGTWKDLTDNVNQMASNLTNQVRNISSVTKAVARGDLSKKVEVAVKGEVLELKNTINSMVEQLQTFASEVTRVAKEVGTDGILGGQATVDNVDGTWKDLTDNVNQMATNLTNQVRDIATVTTAVARGDLSQKVEVAVKGEIHDLKMTVNSMVDQLRDFAAEVTRVAKEVGTEGILGGQATVDNVDGTWKDLTDSVNLMATNLTNQVRSIATVTVAVASGDLSRKVEVDVKGEMLDLKNTINSMVDQLQNFASEVTRVAKEVGTEGILGGQATVKDVDGVWKSLTDSVNLMATNLTNQVRNISDVTKAVARGDLSTKVEVDVKGEVLELKNTINSMVDQLNNFAAEVTRVAKEVGTEGKLGGQAEVHNVDGTWKALTDSVNLMATNLTNQVRNISDVTKAVARGDLSTKVEVDVKGEVLELKNTINSMVDQLNTFAAEVTRVAKEVGTEGKLGGQAEVHNVDGTWKDLTDNVNQMAKNLTNQVRDIANVTKAVAAGDLSLMVTVDVQGEIHDLKMTVNSMVDQLNTFASEVIRVAKEVGTEGILGGQATVKGVAGTWKALTDNVNLMALNLTNQVRDIANVTKAVAKGDLSRKVTVEVQGEVLELKNTINSMVSQLQTFASEVTRVAYEVGTEGKLGGQATVAGVDGTWRDLTENVNQMASNLTTQVRAFSQISAAATAGDLTSYINVQASGDMGSLKAEINQMITNLRETMQRNIVAREAAELANRAKSEFLANMSHEIRTPMNGIIGMTNSTLETELTRHQRENLLIAQSQATQLMLILNDILDISKIEAGRLILEKEDFTPRQLVFNLLKNLAVKATQKNLDLVFDVDINVPTLLVGDSQRVRQVMTNLVGNAIKFTSQGQVSLSIYMNRRWIPRIQNAAQIAHDFMGRDADNFNPTTPINHPSNWSFIDANGEQQVILEICVSDTGIGIESDKVEIIFDMFSQADGSTTRKYGGTGLGLSISKRFVGLMGGEIWVESEIGKGSKFFFTVVVQPGVMTEAMAFKKLERFKSGNILLFDSDPSECDPYEVTSNDYGVLIGGEPPKLYHDLTPLSEDAPDLSSPLSTPSQSVASRSPASGSTSMFSRAISGNGANNSNSGDDKQASSRRLLAPAVPLHQGEQPNPTFSKSDMLLKFLDELNFTGYLVRSIREVDALCNNTANSNSLTFVCIIATNDESVRRVRSIDRLRYVPIIMFTRHPRPGSNRRFVIRHIIDLGMQSIFDTPRTLFDLATALEPGLESFVNMSVQPGKSDPHLHILLAEDNTVNQKLAVRILQQHQNSVTVVQNGKEALECIIAGRYFDMILMDVQMPIMGGYEATQKIREWERRNIHIDDRHIPIVAVTAHAMKGDREKSLAAGMDEYITKPLRADALMQLIRQFYEKGRLRTRPKEKIPRELAF

>[RbHHK2](http://genome.jgi.doe.gov/cgi-bin/dispGeneModel?db=Rambr1&id=29108) Rambr1|29108|

MLAGAEYHNDQRVALTVVAGVSLLVTFGSLRGSLAPLFSAETFLPTLVRLVSATLIHLSVPCLTLLHSRRLGLIVAVCIVVLSVGVGEGLFGVNSTSQLPLPLPLINSVRRANELLQTEGFKVSVPLGPFYEYGIYSLMAWGVAAELGLIYERHLRSQHRALNDATERIRQKSSVLSRVSHELRTPTTAIVSWTELLLIDKTMSEDVRSQLGIVHDAAKHLVVLLNEILDAGKLEATFERTPICNVDLDFCVRDTANMMSQMAVVKGLELIIDYPRSVPTNFRGEDGRLRQVLINLLSNAIKFTSRGTVTIKVEVDELTNENAVVVLTVFDTGIGIPTELMDILFKEYSQLPIHPPSSAGSDGSSSSYTSNISNISNISRDSVSMITHSTATGLGLPSLIQSSAPVSPVTSVSATNSAAEDDEYNNNNNNSNSTIVPGISKSSITFNNRLLRSKARGRSSGNSSSNNSSNSSSNSSGFGSSSGSGSGSGSGSAFSTTSTSTASGSRKSESQTSTLRSRGRSGGGGGGASGDAACVRQAKAGTGLGLSIVKNFVEAMGGTIKADSEYGKGTRFTARIPLGRQVILAAPSAAPSVAPSVASTNTAFSIPQSTASSTISPGSTESDMSYMSTLASMVSNQAMQSDSVYPRPEMGPTLESLKLDSSHLYIVAETSFRDLLAKIFSALPRVTLLSGFDLPEIGQPIQSSDDQSESAPRAQLSGDSECNNNDSASVLHSSPTQDALAKFEEEEVQRSDSGVGPSSSHSRDSSKSSVQQQQQQQQHRRQASMAGRSIFLVDLCPQPHTDDSQEILAENVLHWLLPLSTSSSTPQPAGQRDIIIFFYSFDQLYDKQFTDQFSSHYDIIRSSKPVSERSILNALFHASSAPSAQVPVPPQMPPASILPSPPPLADSLRAPEVFSDTVVGLTEDLAGLLSTRPSGSTNTGFSDSIALPISSGFPATLPPATATASATPGLSVSPELNTRRPPRRNTDILGLRSPHFTPLDMTADTAIGGKTIPPNRRTRSSPVVVPSIQLDGISNGLSGSADASTRLAPCIESPLTATIPQQLQQQQQQQQQQQKQQQQQQQLQRPTSAASTTASLASSELTPLPSLPMLTTSIYSKLRVLVVDDNSMTRSLTVRQLKHQHILNIDEATDGSFACFKYSSAQYDLVLMDLHMPRMDGFLAARRMREIDSQRAKSMQISMASSIATASNTPITATFDSAEFNALKAAVSATSSMRRRRSSSAQSVDGLNSAGCDAPNCIGTCLKVFEDMDASSDCSWTSQPDVATGSQAVTATATATATATTAVTSSTGTATAFHRPVIVALTADRDVNQDAAADSGFDGTLVKPVPLNDFSKLLNKYFGPPDLTMPSST

>[CaHHK1](http://genome.jgi.doe.gov/cgi-bin/dispGeneModel?db=Catan1&id=49608)(ETR) Catan1|49608|

MQPALALACVLVAAVACSAQVASATPAPRPYAHPPAAIEVHLAKQLQQPGDAVQANASPFSSTPLEMSVENAADGITAGAYFAIPLMIWYFQRQLSSRFPLTWVLWLFTAFIMCCGLTHLVRILMAPFWVLTSIKVVTALVSSATAAVLLRLMPDLLTYPMRMSRLEEELGLLIRRDAITQSTDKWAENFRHFVTFIHGRTSLQSTIDAAVGELARSYPKFPSVTVFLPIDTGMAAPASSRRRHPLDSSPANASSNRACRVLRCFAEHHVSASGRSVAGPFFTDYTLETSQRVANSILTQSQIVKLSTVDVAYLLGCARYSSGLAFRLRFGTRTGFIVLGASDETALHLSLAETLRYTDLLTQLQVSLDHAHQYVTRQSAETQLALAVQDRDGMEKRLAAAEAHVKEVSQFVATTSHELRTPMNAIIGFVDVLLEDPSLTADVRDTLDTVMMSSQILLNLVNTILDLAKLEHQGSLTLSSAPFSLRTCVETCVDLIAKRVEHFDLPLNYIIDPLIPDKLFGDRTRITQILANLVSNAAKFTEQGEIFVVVAREPVVFANDTKLVNRRCLDFTGPLVTGAARPAVSFEDPATMIDEIEPEVIEMLPTDFKTPQPASSSYSSAAMHSNLTNKYGSSASSSGLLSSSSMDDDNDDDVDDDIRMTRPLTGSNSANALNSSSSAKPPRGTKRVIAVTSFKTGTSVTSPLLGNKPGPPPPPPPPSTSTAAPSPVPQASASAGRKPMPPFLYFYVVDSGQGISHAGLQRLFERFIQGSDLASAPPSSTHGTGLGLAISAKLVELHAGKIWVQSAPGVGSVFGFALPVRVPYDNGPGLFTNKTPSLRGGLSNDTLVGAPGPQAPAMPIANVDAPPAVLDEVSGLFTPSPRSLLMPPAANNDLVPLSPASTLSSDGTMISPLTTHSNNSGPLAPISLNPLAVLMVAPSASATRHALASMVNAMGGSCWPHTTVIDALHSAQRLRTLAGSNAPLRMVFLVVDPFSSAAPNGHSLARRGSLTSPEMRESTPQAVAFARELAALREVAPVLSISSVRSPVVPVGCCEPEQDPEQVPMANVIVRKPVKFAALYRALVRAVTASASGWPQATGSGEPNPFSSMSGGAGSGTNNQVQNKRLTNEKSFDSPPLVGSVNAHAIASAQTIGRMSTPEGGHLQAHPSPGLGPITPAAVLAMDDPALDWQVQSVLVVDDNPINLKVAVKVISSAGHRGHISTATNGREALELLASKQYDVVFMDVSMPTMDGLEATRLLRERERTGAGSAAAVHWVCAMTASALPEERSACLKAGMNDFISKPIKKDTVIRTLQSAMCQRMSARPPASGTAVVE

>[CaHHK2(ETR)](http://genome.jgi.doe.gov/cgi-bin/dispGeneModel?db=Catan1&id=40330) Catan1|40330|

MSVPTTRSAGELTAIVSDALIAIAYFAIPCQIFYFSRHIRLDGLRGSPMFVVWLFMAFILLCGMTHFFGVWLGGASTTMTVAKALTAAVSVATALVLVRLMPLVLNLPARLFVLEEELGLRIQNERLLQLENSNLHKLRSVTQSIRRSMTYQTICDVATVQLTNHFDLAGCFMFAVDAHHLNRATCVAEYAKVPHLRVASGQLGQTSYSGLPSNVGAPGGPVPMQQVSLASIRRGTRIDLAQLLARQPGAVLGEGFPDLTHLLKRSSSSRQRKSSSLSSSSSAAPMPHSSSTDPIHPPPDCLMDIEDEQPNSSTGNVRYCSQPPLMHVIGGTEMTAASTDHAHWVHLDPHLIESVFGIPMSRVGPGLVGTLVQVFDGSNITPMTTPMATPMNAPLSPNNGTATRDNMTANLSQDQLPSRRQRTDTIPTTTTTGTVPSLSSGSGTSLYQASAEGKRTIMLLLHEPDTVRTQGPVLADAVGQVEIALDQSVQIERDLGRRAQVSVLEREKREAEALNGMKTVFLATISHELRTPMNAIIGFVDLLLSQHELSRDMRDILEIVAVSSNSLLNLVNDILDLSKLEFHGNQFALEEAPVSITDVVEQSIEVVYPVAERKGIQVAAVLNHAVDAVLGDKLRVRQVLVNLLSNAIKFTPNGCVSLTVTSAEPETYYVTAEGAKQRKQRILDFYAPRQASGSIPETVTTMATVGSVPSPPSMRIFFQVRDSGIGIAQDKIHLLFEKFQQLDATIARRFQGTGLGLAITSRLVELHRGRIIVDSVPGVGTLFTIMLCFPPNLDLTQQQGPASPGSSLFAAPSSPTKPSALQLTHLEGGVSLPSSAEVTPVNDVPVVLTGLRVGVISAPNVEMAALKSMLRRLQCIPIELLSVPAPPPTEPLSQVAATAAIRPAHSTAPLAYDVLIVNEPIATDAMTSDDYSALCKWARHLPVVGIAKLKAAVATGNNSLPSSPQAQSARSLKNDCACGGFNITKPVRLRVLESTLRTAIQEFRKPRPITVPSPHRDLNTPPPLTTNEITVDGGSSNTPLLTLNDKLLPTPSSSLEPHTLAPQSPSRPSRSASPTPNSSPISANIDPRYSHLKVLIVDDNNINQMVAVRTLRSIGVTNVNTANNGAEAVAYVEAHPDVDVVFMDVSMPVMDGLEATRAILANAAAKVVMFGVKPVLLGRRTSNSTAVSRSPSHSNLGVPGSIPPVFDGSDGIHGKGLPFICAMTASALPEERTTCLNTGMHDFVPKPIRRNDLLEVLGRYCVWRDARPVPTPAQEAAVAAVLAAANGTTRSGPAAAGAVTQPSSLSPTAALVNGALPSPPAEGSRQPDPRAPSP

>[CaHHK3(CKR)](http://genome.jgi.doe.gov/cgi-bin/dispGeneModel?db=Catan1&id=84299) Catan1|84299|

MSQSIIRPHSHSVFPMVSSHVHQRLSAGRARTDHAETEAARASMDDRPLYSPLAHLYHEQAFTQTRPMMWEPGLAGPSGPPSHAIHISATSSCRVADQLSEGQLDKFQSSWLVRVWERRHHIGAVVASLAFFFIGLSVALILFFWFQRVHKSYYTTELHRSCTSLGNLLSLDISTNLVTYADDLAGYFATISNVTEATITRFTSLAQDHSTTSDGISIIPVFAREQLPTFVTQFSNVRFQDGNDTFTRLGPVAPIALRLPFPPLRAGFLNPIGLNLFHASEYASTIRRTLGTGLPSLSLAEPGSKTIKPPNIRFGIMKGVQGVAVPNFPLWVMSFMIDSLRAIGRTLSSERVNGMAVRLVDNRTTSEYFSSSPGSSLLPTDRSVTTFDVVDRQWTLTCTATAHMRSMYRTPWSWVLLFLLISVFTHREGLVASLRMYSRAIVSTVRDPLIALDAFGYLVGSNKEALARLGIHGSSTSPLHISSLLPAGCIPASSSVAHGGEETTDSGDPVGFGPLPPGSYEIGMVGRPEAPFVAEATVSQLTGVKRGQVSQVLLLHDLTERIQALKLLQAAKDSAKAASVAKSQLLLLISHELRNPIYVINSLIEDPQAALTFGTPADDTELVAAKRIHHTTGLVADILGYLVELFAESAWTSTVPSVHGGLLDVRKAGLRQGAPATQLLSIANAALASVQPQLSLWDISVERWIEREMFLQGPTRLVKDILAKVAYVIADATQPQGQAVLELLVDKHNRDIRLYCRGEMWIPPQRSLQFVTSAKTGTRTTPSVRSRSTPWTSEQANAPEHVDSLTHAAIFLAMNVISAALGPNNSLWRVQYTPTYGGFDMIIPLGRVRAFSGPYIVSASAKHSAQLRERAHEQAALRDEVVSIVPGIAIPPAPTVDAITSSPSRAVVSPQPIYPSPLSPNRLALKSESQVAAMAIQAQKPTPPTHSESRYCHTCPIDPSPVIGPYAFPPNVGRSCCYAQQTTKSNLEQQPASSRSHVSNAILVQL

>[CaHHK4(CKR)](http://genome.jgi.doe.gov/cgi-bin/dispGeneModel?db=Catan1&id=64655) Catan1|64655|

MHQHIFAPNSVSGPGPTSNSHASSQQQRPQQRPNASSISQGAVGSSPRRQSRRTVGSPPPPAPTPRSGSPLPWAAPPSSPAVLPSWKSPDLSTSSSSSRRSDRAQLVRLVLMWIVPMLIFFTSVFMAIYANRQILWTEEDSLQTVTSFRCDQQLDRLANEIPSRMLDPARFFQAYVQQAQTINQDALNDFFFGSNVSTYLRFHLHPRVLNQDRAQWERANAPLQITVPADPAFVTTPKGTDAMYPPPRSQLNNGMVPRPINASPVYWPVQFASPQRGVSLGFDFYYGPRTLAVDRVLRTGEPVLSDVVYLYMPLEDPETLQPGMVLFTKPVVRSEDNSTWMVSATVNLIPYLSGTLGKSAPAARIYAKVEIGGVPVYETERVQPGVGIAARAPYIKSAVIGQQNITMVCLPTAFLVGMFVTGWSIAAPLLTVFGGAGLAWMIATLVDRLQRLRTAESLLLSWRSYSEAILQATPNPMILVNDQGNIAGVNEPLLELTGHTTDSIKLVTKLCDLIVPLRKDPDEELFSALSVVGGGSRAGTGRAADDASLPVRATVASYSAGSAWRQNQQQQQQQQQSQPAPPDSTSIQIAPDDDETNANADTTDLQAIHDLLLAPGRREVLVVPAPALTSDPADLDAASTIDAMVTVSEPTGNPVIQHVLVLTDLREQRARDRQLAELLRETELLNAQQRQLLLYLAHELRNPVYVIQGSVQAAADAAAEQVEKNGIGGAGTMTSGEQHLRSSAAVSFAATEASDRAGVLAATEHVTTLLDAVVEYVEASSAAMAVFPYTSPGSRGAGIVGRVGVGAGGTLVRPSEAIAAATLARSTYGRAASALRAGHASGSSPKQSRSVPRPLPADARLSSPHRMVSPTTLIPAAAASAASVVRPAAIGLSDALSLADVISPATALALNPDWTHLDIGCSFNSLPVSALTPRTPDFLRRPPCVQLSRASRHALHKLSAVCRAAPGVTLAGWSVTLPHPPRLPPPLPGAAAPLVLESETYVTLCAALALPDSIGHADLTTLSSPFDWSISGQGSEFRLFGLQVAILTRLAELAGGMAKVDQANREIVLQVPARPCSWVAQGLPCEGVRATGEIGLMEVSGLGASSSHIGQPVKVSKAQQPQLQRPGIIAGGVRARPATPPSTPSPEAAAGKDEHTATLPTVTSVSTVTGHGDDVELQPLLPSTLQTCDSAATVIVDQADPSTDSKAAKSDIQAPPMSPVLPTLVSVSPVPAPDHHECPSEASSAPASPLVVLLVEDNALIQALTKRFLERDGFHVVVADHGQAALDLLADPDLSSRVHLVVMDLMMPVLDGIDATRIIRQTRSPAELPIVALTANAMAEERERCLQSGFNAFLTKPVTRQKLIETVRSLWSASPSPVATTIAPVQA

>[CaHHK5(CKR)](http://genome.jgi.doe.gov/cgi-bin/dispGeneModel?db=Catan1&id=74130) Catan1|74130|

MSPPLTQPPPFGLGPSPGSHLLPADPLTARGMGSRGPLGSNQHLLANTQNRLGGVPPMSTPLPSRFPTAGHPIVPGPAVYTLPTKPSATVLDPAHDNAFQRSWLVRLWIRRHQCAAMVVSLVFFMGGLSVALVLFFWFRGVHRSYYSNDLQRTCLSLGDSLALDIATNLVTYADDLAGYVATEPNLNNAAVTKFTSLATNHSEETDGISLVAVLPRHRKPEWEKQFNLTMIDGNDTFTRLSLVAPITLRVQFTPSRTPMKNPIGYNMFQAGEFARSVRRTLGNGRPSLVVAEPGSTQLHPPHKRVWIAKGVQQVPVPNFHIWIMTLLVDTQRAVSRTLAPSDRTHGIRVKLVDNTTTSELYVSQFPPNSTLIPSDTTLTSFPVVDRNWTLTCTGTHTLRGAFYTPWSWVLLFLLISVFTVSAEIARRAAVRFLAARGVARQVKHREGLVASLRMYSRAIVSTVRDPLIALDPFGYLVGSNKEALARLDIHGPDTSSVHISSLLPPEFVANVMNTPHQLPVASPAVPPMVDQDWDSVYTVTGGAASDPLPLLEPLPPGSYEVGPVGTQSAKFIAEATVSQPTAAKRGEVSQVLLLHDVTERIQALESLQAAREAAKAASVAKSQLLLLISHELRNPIYVISTLVEEIAAVAATADPLAPASGDSIGMRPARTDDSEYIAAARVQHTTRLIADILGFLVDLFADTTALEAARLAATVSGGPKTARAKEPGKHLISLANAALASVHPQLDLWDITVERWISPEWVLHGPTVTVERILAKVAYIVAHATRPYGQAVLELLVDLRNRELRVYCRGDMWIPTENSLQPMASIPPQSPLATSLAPSVQQEKLVMWHEDKIAVPEHIDPLTHAALFLAMGSVCAIVLPMNGSAFRTQFSEQYGGLDMSIPLGLMRAKRVKCTEASGEDTQAVVPVPAAATAPFSEELVSLASISSLAMGTPARPSLPFDGPQRNLAPSSDAYPSPISPDRVTLPLPKMTVPQVVPIDSIAVHPKRYDENHRKELFTPCHSRTNSFSNPASFPQSPSSAPRTPRTAYSPCPPPNSRRESRMTSLMAVRTMTPHSPSPPASVGVAPPAPSSSASNESPSSDLSHASPLDFQPLIPSNDTLPLHIPLPSSPAPSAKSPKLSVPAALSPRSAPRLTLARVPTPVATPTPATLDVFPSFPATSSILFPTPTSQVISTTLHKPSPPNLSPISISGTPVQKPPVAAPVASVQPTNHRPSAGPKRILVVEDNTLVLRVTCKLVKTLGYEADTAMDGLECVKRMNDESKPKVDLILMDLVMPNMSGMDAMKTLRDAGYSKDALPIVAVTANALPEEREACMEAGFNEFVTKPLKKDGLVDICKRFIFEVVD

>[CaHHK6](http://genome.jgi.doe.gov/cgi-bin/dispGeneModel?db=Catan1&id=397421) Catan1|397421|

MSLSRIPGCSDESVRAQQQQLLSSTVIALVALVPALASLFLAWVSRLQQQRLAGTTATSPSPLQHSLTVRQQRLIMVAHVVLLSTSASFVLGLGVSIAWGFRPVSTGHTPPGSTSRLLAVLLVGNAISVITFQTAGECAMLYQFLCLYPSTRARVAVLPLAGILFLARLASQVANHVLRLLIFAMPSSPTMPLWQRLHEQIIYVYTFSLMGIILLLSASFVFKLWTRWRQYCADNRGRLRHHGSMHRVRSVFIASCHHTVITSIAVILMTHRQISHLCFDPPALAQASYPWHLGLQRMPHRPCHCRYHAVSLRDMEAHELGDSATRPVDAQQPPVFRYNSWDSGTLSMTRPTSDLHMTHSHCQPPGHTMHDSWPIVSTAIVTDIGSPSTHFASSARPSPVDGPSQSRSNDPPNQLNLAAQTRHGPDAVIREFTHFIGFRFATRARFFSSSLIGSEAGSGLSASGTVLALAIRECHDTMTELLFVPSSSSPTSPDEKPCALLVPIHFNGIKLGVLVCTPWKHVESTGQNLERCTAECCLAKRNLTQSTMAPSRMSGLCQSVCKDRVHELLRRLSGFFAAYLHSAKYAKSLPLSLPTFIDCFVLACRQRRVNQETVRQFQHQLALVEATAQAKGTFLANMSHEIRTPAAQVVQAAHILSETQLDSVQQEHVEVILKSSELLLAILNDILDLNKLENGKIRFENRPFDLHDAIRQSVDAYAGNKAKPLVDVGYVIDPDVPVTVVGDVVRFRQIWNNLLSNAVKFTESGSIILRAELIATTCRDSDGGRAVIRPSVADTGSGIPEDRLARIFERFEQSDESRYAFPTVWFVLRVDLYTRARICPGVTRLYGGTGLGLSISESLCLLMNSSLHVTSTVGQGSTFAFEVNLGFVPCTPLPEPPPRQTVAVLPAPNVPLPDSIRIGTIAQLVSSRCCVVPSQEAHLADLQLVVHANDWPMLNDKVPAIPTVFPCSTSAPPPIAHHAHIAFVSRPFKQSHLFRILDSLPAVTSGAMAALPADPSKEPPTGVTRHDSKISHDATAGLTPPPPPLESSAATSPPTVALATAKTQTPSLPPSFRVLVVDDNPLNRRLLVATLTRLGHATEQACDGKEAVDRVLHDDKPQLDCVFMDFRMPLMDGLEATRRIVHHYSTHDSKQRRRPIIIGLSADAMTEQEDEGLLNGMDKYLRKPVLKKDLFQ

>[CaHHK7](http://genome.jgi.doe.gov/cgi-bin/dispGeneModel?db=Catan1&id=156475) Catan1|156475|

MPIVVAQHGVSGSESLPIAARAAGLAARHSRFPAAHGIHVDPEPDTRGAATALGSSRVIYPQPALASTSASFLVAQSALSPAAALVHANLAGSDLLSSIIFVLVAPIPILVSLFLAWLTHLPDRSRPYSRQDGYGQHPQGKANRNPQRRHLRRQHWLMVAAHVLLIATSLNFLLTATLGFLKVLVHIGILSLASSTESHPQLLSKEVVLGMLNGIASVTAQTTGEAALVYQFARIYPSSSARWRILPMALLFILVRLTAHVSLNTLVIWSIVDPSVSATLRSYQRIAAACFSSSVTAITLLLSATFVHKLSSRWRRHVASQLNAAKSRGLESSAEPLLPTMQRIRAILVASWHHTVLTSIFSLAMTASFPFAQSFNAAYWINLVATTAFKLSFSLGISRSLYHMAYDSVLRHQIQFLATVNPQSRGARGELRRDCEERARRRRRRRAEQPGHGEVQEGVRLSLSSSAASSFIYNSWGSADSDSDRHEGPDSGAETDIQVVVDTAIPLSEWPISTENIETDPHVLSPSGLGGNALPLSPAAVEATSLAVPNHASSPRPSKHPRSRALSTEPPVWDTFLVTPHHQEPWPIRSSVIESDPGHSYHCSSSATHAVPKDKKPQEVIDSSEGKRPAPAKRINLAMNSLRGPQEVVDHFSEYVCHRFATHAQFFPASLVRESPIDSTTTIIPPPPAASCPAPENDSAATLPPHQPLSAASSASNEHIPSAQQPPSLPAEHAPGARLCYQTMMEVLSLPPAVFFASRRATGTKEPILSDTAGTAKSTKPVLFLPVHFNGRKLGVFVLAPWDPPGKLGKSFGARTVSAAASASAPDGRRTRSRAGSQQRPDSGLRAVSDQLVQGPLNDPAVELVKQLVRFFAAYYYSAQERQLNQETMRKFQERLAVMEATANAKSTFLANMSHEIRTPAAQVIQAVHMLSETDLNSIQREHVQVIIRSSELLLAILNDVLDLSKLENGKIRFENRTFDLHEALRQSVDAYTNPRKPVDVGCVMDRALPQFVVGDVVRLRQIFNNLLSNAVKFTNVGHVLLRAMLVSMDSTSARVRFSVQGTYTGCGIPQDRLASIFERFEQSDETITRLYGGTGLGLSICQSLCTLMNSSLEVTSELNRGSDFSFEVSLPFAPPDHIVPPHIHSPPPPILDPTAPPRLIRLVICTGPAAERPPQLPPAALRGTSSRVVATNSGSSNVLSGKSRPHDVMNDLLANYGIVVATVYTTCNALMDSANATGTGPKPNIVVITCPKRWPPIPPAPPRLPTVIVSGCDMLASADGGPTLVPPHSPYSNLAYVARPFKQTHLYRVLDTVAGLGDALPLSTLINGHSGGDSALVAEPPPIPRSPNAGSVVGGADTKADVDPQFNRLPSPTCACDGSAATGAPHNAPSVIASDLSSTAHTAAGSGLVAGGYAPLRVLVVDDNPLNRKLMQAMVAKLGHEVELAENGKVAVDRVLAMPAASIGDPAADGARVEADEDDTGVANVDCIFMDVRMPVLDGLEATRLIVAHFDSLPDPAQRARRPVIFGLSADALQESEETGLFHGMDVYLRKPLLRKDIETALNQYFGPNGLRHKKQP

>[CaHHK8](http://genome.jgi.doe.gov/cgi-bin/dispGeneModel?db=Catan1&id=54886) Catan1|54886|

MPAPDTAKHEEIPAVAGTCFPNHVKPGDTGFWNAKNPASNAEMILTTLSYETRTMLTNIVGSADTINSFQSSLPPELRESAQTIMQSSRQLLRTIQSVFLLVKLSEKSLGVQPKPVSLCEALVEACQRNERFARDRKIPISASSSALSDWVMTDITALATVFDHFLEFFTRNLAPTTPLDIDMSSSTISAKDSQAPVQCKHLIHIRFAGRGTKLPPVLAQRIAGLPIKHSFLDVLRKMQVLPSYDCLLFSVAHDVVQLMGGSVSPLENGVVINLPLVETTAPPSGQYVDSSEGLYDIHAQKQQEQQQATKVSPTTAQSPAKNAVAEIQTGGDHFVSAHSSIDRSAGDQQQQGGVIQAPELPTIASVSGSGITLAASSSTQSSQGKQTKSSVEGAAGVPQVDAAIVSPSQSHTLRLPSINGTLANGSVVRVLPLSQDACPVPHQKPDGPPGKPKSERHLLVVEDNMINQRILRNMLHKLGYTQITFAADGADALFHYNSLKDEGKYFDVILMDISLPSLSGNDTCAWIRQKDRTQVIVMCTADTQMVGNMARWKACGYDDAIEKPVFVDVLSTILNRWLEKGDARRARHAANMPSGADVRGAATNARVK

>[GpHHK1(VIII/Fph)](http://genome.jgi.doe.gov/cgi-bin/dispGeneModel?db=Ganpr1&id=123888) Ganpr1|123888|

MESPVAKISPDGSHRMISRVFQIVHCRSLDEPSLQSSFFETAPFQVDPRRNEESENRHLSSGGGISDGFTADGQTGAQTLTDEDNGSVFIRYPNGPFIRCEDEQIHIPGAVQSFGALFALDATTLLVRQCTVNSARFTRLTPAALFRLTSFTEILSEGSGSLLVETLKDLEDVDKDGAPITFVLDLPGGQCWVAAHKFSKNPGLVVLEAELLDDELQPHTPQASSCSEAIANAAESLADQSSATDLATIEVMESTEYEPMRLPRALFSTNGSILSTMETFRILDEITAKIEGAKSVQELAFVVVRIVNQLAGFHRTLVYHFDEFWNGSVEAEYVDKTATSPSRYMGLKFPESDIPPQARALYQLNKVRLLYDRAATMCNLVCRSLEDTKRPLDMSHCFLRAMSPIHITYLGNMGIQASLSISIVAFGRLWGLVTCHHYDRRRNSSPLRKLCRVVGEIAGRNLERLAFVSRLSAQDKLAKFAYKSMSNSSLIEEMISSSVMDFLSLFRADWGVLSIEDEARILGTSVPSHEVLALLSFLRAQNIVTIRKTHHVKVAFPEFTFPGQRKIAGLLHVPLSDNGHNFITFLRAEQITEVKWGGNPAKEPSKPLEPHTSFQTWVEYQKDRSSLWTESDLELVALLQLVYWKFLAVWQEKEAALATSKMKTVLLANVSHEVRTPLNAIINYIEIALEQPLASGVRDALTQAHVASESLTILISDLLDLTRIETGKQILFRNKPFSPSNIVLSVASMFESETNKKGVVLRVEIGENLPAVVIGDAGKIKQIAVNLMSNALKFTEAGEIVISCRVLETSDEEANIEWAVKDSGCGIPEEKLSLIFEEFEQVESALSKNADGMGLGLAIVARFVKNMQGQLRLQSAVNAGSLFSVRIALPTRSHFVPALAQERDVANPDLETSGISDVQSNSPLSAPLPIALVSREGHAASYTIFPDIVSPTDEARPSPSSDATTVGSILVDPSPASTLPSPPSRGTMPPVPLRILIAEDNAINQKILQQRLKKDGHDVVITDDGKQCLDVFIQNYESNPFEICIVDLQMPILDGFQCCSEIRSFEQSRSSRDSPTRSPISSSPVDLPFKTPQTVPVITPKPVNGTSIVSSSSDSASPPSLVRKRLPGSTFSLSGRMPSTLSVSSTPWRTPIIACTAQSHASDAEMCQLGGMNGFIAKPIDFKRLRLILGGVRDLSLRSAVKAMLTSENREVGGWF

>[GpHHK2(ETR)](http://genome.jgi.doe.gov/cgi-bin/dispGeneModel?db=Ganpr1&id=220489) Ganpr1|220489|

MGCYRGPRSQMTITRLVSLTIFLVGTACLFGFTLARGIPIPGGESVKEYADAASGDSAVHGTRRNGTFSPDFSGGHLFQHGHVVFFLSSSGDVGMDEEITVELVLEGARIPLDLPWVHNGPLGLLKTTTAPVSGSCRLYWFEVSKSSSFVARWPVYGALTTKESEGWCPSPTDPNLALFSSVLDLLIALAYFSIPFELIFFIRRSPHFPFPSVFFMFGAFIASCGATHIVGALVPWPYLPLYGAHIAVKLATAAVSLFTAGALLIIIPKAFELPVYARAMELALSERLLTVRSLRDENESLEAFRTITHGIRTKALDSRPDILRFTGSEVAQVFLRGDQRWGRCLVYLPFVQEKGGSESRQTEAFLWCFYEHVKGEDEDSVASVKSVILSANMPSVRLSLTSTAPVEVNDSTMPDDLSVFQDLFGGYENGVITSVLLVRILVPQNTSVFSADADLDTVLSTASESFLLSGLESTSHSGEPHSYGLIVLVRHIPAHRVFETGDELTEPFLSTRNLYTPLKQIPPRALRREFPQAFTWGVNTSLLSDVVEQTSISLARASLLDGNKLRIKQLAEQNVVLMQARKEAQLAKGQREFLAIMSHEMRTPLYAVTALSSMMLELPFLQNFQNSTSTIEERQVLELIDMLEVVKRSGDMLITIVNNILDFTKVEEEALNLEKRPFGVREAIETSMEIVTVQDDKGSNPHINVKIDPVVPIALVGDFTRVRQIIVNLLSNACKFTPRDGDVLITLNAEPVNQSGQDVQTRRKVKLLGAVIDTGVGVPEEHRKKLFVKFSQADASITRKFGGTGLGLAIVKKLCALMEGDVSVDDNTDAKKGTVFRFNLTLDSYHEGDWDVKSRPPPPLVQNPAFSNKHVWCIDFHDRNLEGIASTLDAAGCHNYSMAHSLVEFLAVIGAVSPTEQPFLPQTCHGVIIDMRTIVEKEELSLMRRFLSWWPDSPKRIMILCNPALHKVARRQIGYLGTRMSGAPGSSPESIVMATKPVKYSSLWKFLNGLPNGPVPNHPGPEGHYLGDINSLSSYSSAVSIPMTPPVLPHRHSNRLAQALTIIPLAGDERSPSESLHSRNFRASQQPPPTPGSFLTASPGSSPASNDASFAAAVSGPTRESQHSRDYFSFQPKRSRGSAHPTSRQYDTSAKGDERESGTSDHPTETGLPPRLATLSAPSRPPLMVTLAQSNSEVPLPSYNILVVEDNQINQMVTGRILQKLGQKFEVADDGIKALALFQEGRSFDIVLMDISMPRMDGYQCAREIRKTLGGNAPERRSAASPTKPWIIALTANALYDDRVRALEAGMNDFVPKPAKQSDIVDALVRYLEQG

>[GpHHK3](http://genome.jgi.doe.gov/cgi-bin/dispGeneModel?db=Ganpr1&id=285950)(CKR) Ganpr1|285901|

MPSGSDRKNDRPSPRGQAFRHSPRASDVLSLYSVASAPRNRHPMLSSEDLLITKNSPSTPPGPKGDYFTVPAFASPLAPEALPDAWRSNRNRFWSLFALASVIVAAAVFGFALYYSTQIVSPDTQRDLLLSEITIECNQRARFLQRQMQIVEGGIRDIVANYHVATLEGGQFTEAEFTTYMKFSSISQLAIRGAAFAPVVTGAGRSQWESQHNLTIHNDNTTDVPAPDRDFYLPLLYRYFLSPADAQLVSATMGYDILSLWPFLVGSSLVSGLPSLSAATSFATKDGPVPGLVYMSPIFVNATARDALDSSPFLFDASAFRNVVGFMYATIASAQFFPTYLTPDASDLNYAFRVLDTGSGRNFGTPELFSTWNDSWPGAYIAQYAINATIVVTNRTWTLECTPTKSLVNERITSVPIKTIVVTVAVIFGVVLVLVAAMLIRRAYRFGRKAQNALQALFSDSESIVRAIPDPLLALDDDDQVVGANQLALDATGYSAGDIGDLKLQQLIQGYGDKLGTGKRGAGDQLFKVVRKNGSSFPVEISMSPRGRKSGSDQSPSAAGSSVFSRVLVFRDITEKYERENALVEARMYAEAADKRTESVLRYLCHELRNPLHVVIGYTQLVLNRHSDATIPSDGGMSSGNLPSIFVSATDRDELKEVMEASRYISSLIEDVLTFVDLSRNETQSPDRSFDMTSLLSLVSQRPGFEVSIDWYGHRKFIGKEERVTEALAKVLDYLVFIRPDNGVLALEGRSSTLRGGYELVEVSLESRSKTHGIDVFDKTEFFDSPLLAELKREPFAENGSSRGKKFGAPGIWLSVARVIVERCNGSFSILQSDSGRIRAILKFTFKRSNSADRILRHSCDLGLMLGSDLGRRPPLDALEMSSILLRPSLELAVICHSSSTSKTLPTEDETPTSDPVRDPVRNSGDGDLEHLSENIPELPSVNQTPTRNDGVIATPTLDSPAALIASGTSQSGTFDAITIVSTRPDSGPLKQRSSETDTIILVVEDNGLVAKLTQRMLKRGGFKSEIATDGVVCVDMVRNIGIEGISLVLMDLQMPRLDGFGAARHLRSDLHFAGPIVALTAFTTNNDVEQCLEGSLMQEVLGKPVTEAKLLETVRKFIGWRLHNPP

>[GpHHK4](http://genome.jgi.doe.gov/cgi-bin/dispGeneModel?db=Ganpr1&id=468424)(CKR) Ganpr1|468424|

MPHRQAGIISRTGGIEVPDAAALLITGDVGPPGGGAASVLSGTPTKKFFNSWTWTVFIFICTLLVAFGIGMGLYFTAQTIPVADQIANFESSVQNECQARMRNVVLAMNLISDIVQDHVAGISSITNLTKAKFDTFTGVSAINSLAVRSFVYAEAVSESARPAWEKANNAAITELTIFQNGSIDYSIPPYNASSAPLPYYLPLLYRYYPRSVTNTSISTVPGNDNVKLREPLLNRSLRSRKPALSSPVILANNNXELGFAYIAPVFFPNASNAPLXNITDXYDPXAWAGLRGYLVLTFPSNLFFSAYLVGNISIDGNGTAVNQTTDTLFVSLARNKELTPLPKRMRLTVLDSSDVVASGIPVIYTMLERTSETLLQEYTKNVSGIASDRNWTLICTPTEYYFNQNVSNVDVTKIVLIVFSVIGVVLASFVAYLAIQFFRARKRTKNMIEALYSDSKSIVKAIPDPLIALDDAGRIIGANKAALDATGYSAEDIGNLLGSQLLVVSDVLHKGSPKPKLGTLENLGSHWDTHESRKSYTRQPTSLNGLRAIDIIRKDGSRFPAEATFSQGVLLDDDEVLQSKEEYDNAIKHPKRRHFRFHRSRAESGAKMIPLSRRFARVVVFRDVTEKYEKDQALRAALERAEEADRRTERVLRFLCHELRNPVHAIVGYSDLMLHEDDISLSPERAHLPHRIEELQSISKAGNHISSLVTDVLLYTDLQRDQLQLKESTVDMKRFLNGTWRGGTVKVVGQWLDSEDRHFLGDYARLRDAIRKMLDYAVLTLGENGGEVLVEGRISKAPLDAVLFEGARTVEVSITSARRAPSSDGSXSIHSERDSDXQNLPTPGSSGSNPFESRSSSFHVSPLLAALDKDPFHERGSSTGQNFGASGIWLAVARSLSERMGGSLTLTENGDGSIRAVLTFVLKIRSPGRLRLNREEDLTLRLQEXRKSEDIIPPSPSTLXSPTHNTIGISETLLXTSKPAARPETPEAIRPSPLVVSSLINSLSTLPADELGLAASNAWGAKESTGPQFFLSSEGLVAPATLSSAPEQNTAAISTSTTLENVPFFSQHENQLFSDRQNNEIDVHRRHSHLSGDSSDQDVTRGRATLESSVEPPDGNVAGPKTPESLSTLTSTAAKLAAEVDVALSALRQEANIEHSDDHPVPAPSSQGNVAPRSQRVVKKRPKKSTIAGKDISLVPEMDPSPPMGHVLVVEDNIVVAKVTSKMLARNGYQVDLADDGLKCVQRVKVRALNGYDVILMDLHMPNLDGVGAAQQLRELGFDRAIVALTGVSKSYTAQEDQRKCLDSGLMQAVLGKPVKEQDLINCVTRYSGWRR

>[GpHHK5](http://genome.jgi.doe.gov/cgi-bin/dispGeneModel?db=Ganpr1&id=50619) Ganpr1|50619|

MSSGADTLPSPPLSVGAGCCSECREERALLTLRVAGLEEENAALKQAKSLQNGSINISLAVSNTNETKMPKFMKSSYLLCLEKSITGHPINVRPIWSNRPIMGDESELNSQMLLSEQVTAILTECWDTGQNGCWRVGLQSHKENEPSLTYQRVEGLAGSSVREILEVMRAAADRGRLPGDKLMTVECFGTCDAGPLFLCSLYDEIEKVGNQRSQILQTILDITPAWFSITDVTHHVLFVNDNRYFRELSGCTPSEVTDFGWAAHAEAGNGPEIADSVTGAGVVEMGWRRWDGSVAQIRTTSIPFKDEVTGEVFRVGCSVDRALEYQLELKNKELDVALHKAEEASRTKDLLIAKVSHELRTPLNGIMGMTRLLRDSEDLSPELQDYLNVIWESGSGLQTVINDLLDYSRMELGKFELEPSSIEIRGLCEAVMATFRAAVLANKSLKLSLDVSPDIPTTVLADGPRLRQILLNLVGNSLKFTEKGHVLLRVRKPDSASQRKQNQITLLFEVEDTGIGIPPRLQGKLFKPFSQVDDSIKRSYGGTGLGLAIAKQIVELMSGRIWVSSETGRGSTFSFTAIFPEELPAAKKSRTGGSPRYASFQLSAQSPLRKVKPRILLAEDNRINQIVAIKFLEKVGLSAEIAVNGREAVDTVIEGARKGEPFDIILMDVMMPTMSGIDAAEEIARRRDELPRRPHIIALTASASEGDIRKCLVYMQDFLSKPVLLERLVELLERVANSMLGH

>[GpHHK6](http://genome.jgi.doe.gov/cgi-bin/dispGeneModel?db=Ganpr1&id=40982) Ganpr1|40982|

MRAPTLAAAVIAAALALALPRASDAAPPLAVGAICTDSSQCSGWCVSLDMGDATKACSCSKASTWDWGSSGLDVFCALLMPRVSQSLRSPVSPANAHAQMFAAVETTSSSTNAQTVGNQSLPTNPASRRQQRLRREPRARRRKLKPHRVPGSLSPPSLLHVQRNVVHHYQDANRNVEHYHRDPNPIHDQRHNNRYYVRDCHNDRNYDGNSDDHRNYDGNSDNDRNYDGNSDNDCPIESVFDLDSETMSTSTTQTITVTTATETTQSTTSTETSSETETTSATATETAATSATVVETPTTGQTVTATAMTSATSTGRPRKSTTTTKPKPVTTSKTTQTRTATTIKTVPPKPTTTTKAAAKPTTTTTAAPVKTTTIVQVRSCDTIYSAYLAGVKMTDADYADWNTWSCKTWFPKGIGSPSTPPPPPPPPPSTRSCDSIYAAYTAGTKMTSQDYSDWAAWGCKTWFPNGIGSPSTPPPPPPPPSTRTCDTIHAAYVSGKAMTSQDFTDWASWQCSKYWPGGIGSPTVRSCDTIYAAATNGVKLTAQDDSDWAACCTKFKRRSISNSSIAIPNLPKRSAVSSSPSPMLQRDGHENELGCPRCRSEIARLSERIKILESQLNELQSYPNLSLRLRTHDEIPPEVDENYYRTNLIFRLEEHSGPDGITAKVARRVYHSHPRNMNGVLDENGLFLDLVPGWDDDHKALLEAMTRAYETRKSQRWYTQPCVTSPAVSNLRELVRLLAGRASGKSENSLMGIVDFLGEDKAGPLFLCTRGYLFLASSFLYTEDDMLRAQERRVFQQILDLVPVWIYWCDMDGNILYTVDNEYFRKLSSCTPEDVKGEAFDAGLVDSLWKRKDGKVSYIRSKNVAVTDVESGIAFRYGYTVDRGLEHELLVKNEQLLDALERAEAGSRTKDLLLANVSHELRTPLNGLLGMARLLRETPGLSPDQMEYVDGVEESGIVLQKVIEDLLEFSRMEMGKTSLEAAPFSVRGVCEGVVSTLNAAVLKRPNVTLNVDIADDVPDTLIGDSGRLWQILMNLTGNALKFTEEGTVRLSVCRSAGPADLQKDTEESLQKAIVDAPKQSGVWLLFSVSDTGIGIPEPMIDRLFKPFFQVDPSTNRKYGGTGLGLAIAKQLVEIMGGSITVKSRSSAPNQGSTFSFTIRAAIETQPHANGSNTTDEASSPQHSQPRSRPAPSKGPSFELKRDSPLRLHKPRILIAEDNEINQRVVVKLLSKLALSSEIASDGREAVDSALRAVQEGKPFDVILMDIMMPRMSGIEATHAIRAFPPEYPQPHIIALTANASERDVSSYLKIMEGFVSKPIQFENFVTTLEHVVRLRNAD

>[GpHHK7](http://genome.jgi.doe.gov/cgi-bin/dispGeneModel?db=Ganpr1&id=56747) Ganpr1|56747|

MPAPERVDACACGCLPCNASRSRMAARIADLEAELEEIRRGPSPREVIFTVRGPVGDQAPVYPDFKLSTCIVRLETETEIRRLYVSHPLAPSIVEGSVGDLSADSHKLVLSALHEARATGRPCGLQSVVSAQSFVRFDPSNPPEMSVRDIIQQRSSPSRGERQCLHIIEHIGESSEGPLFLIAVYDPVRVYRGQLLRLYETFMDHVPAFLHLAETTEPFDIIWVKDNPYIKSMVGRSGSEFLGQGWRDVRLGEWHAEDLPPPDPATGSMTISYNWQKPDGTPVPVRTTHLPYRDQVTGEDFLLGYSVERELEQRLIDQNRELESASRAKNLLLANVSHELRTPLNGVLGMASLLRDSCGLSKEQEELVDIITQSGNGLRSVINDLLDYSRLEAGKTVLQLDRFSLRELCSSVIAPFRPVVLSKRTTTLTLDISREVPDIFMGDASRLRQVLLNLVGNAMKYTDVGTVFLKVQGVGDIPLENGRLHHDEGSMPIGDVGSTPIGTGTPLLFQVQDTGVGIPKDKQSSLFQPFSQVDSSYKRQYEGTGLGLAIVKSILDLMGGRIWVDSSPGVGSTFSFSIALPTAPLTPSPEPEDPFQHRLQPSRRRRSFELSPTSPLRTYQPRILLAEDNKVNALVVMKFLSKVGLSAETAVDGREAVQKALDAASAGKHFDVILMDIQMPVLDGMEASEAIQRSLSPQQRPFIVALSAAAAPSNVEKCLRIMDLFLAKPVRLEDLVATLETFARMRPTEPRA

>[GpHHK8](http://genome.jgi.doe.gov/cgi-bin/dispGeneModel?db=Ganpr1&id=57155) Ganpr1|57155|

MLDAGVSCSACGREITEFHSKIAALEEEVSRLRALLWTNEEGFAAQDVDDTQGSEIKPTLLVSQRDVDHSDDTLASDYHGSTHGSLKGSEVEQSSQILPPVQQFDKSFDVFRRCYSENATRGGGETLVDSFYDRLKEVLDTVPAAVTWSDMDNNVLFALENKYWYELTQCTLDDVLGMRWLKYVSVPCGASAPIGDGSDVGLWTRKDGTSVTMGSTTYYHTDPATGIKYQLKITVNRHFENELFFKNQELAQALQLAESASRAKDRLVASVSHELRTPLNGMMGTARLLQESENLDAEQRELVDAMSESGEALKSVIDDLLVYSSMELGKAKLNPEPFSLRSVCRSVVVNFRASTLKKASVSLSLQIEDNVPDALMGDAVRLRQVMFNLVGNALKFTDAGSVAVTVRLAESRDVGTEERPAEAQVPKEPDQSAVEPVGLFFEVKDTGRGIPPELQDQLFKPFSRVEQVQRYSGTGLGLSISKQIVEMMSGRLWMESDGSGAGSAFKFTSTFGKLATVDDVNLKSPTSPRLSNSKSIRGTRRPPFILSPTSPLFGLNPPLRVLLAEDNPVSRMVATKFLGKLGVRADDAHDGQEAVDMCQRGNVDGKGYEVVLMDGQMPKLTGLEAAHAIRALLAPLEQPYIIALTANVTEGDIAQARSAGIREHLGKPVSLEDLTKALERAVVEKRRGEPPC

>[GpHHK9](http://genome.jgi.doe.gov/cgi-bin/dispGeneModel?db=Ganpr1&id=207463) Ganpr1|207463|

MGPSPFSCSVVRLETMDDGSQDIRKLYLTETNRRRYYWYQRPVNALLGRDLGRPQAFVDDIITACHEALQTGHRARRILFGKAPAVIPTPNTTIQQLFIISSKSMDVHSDRSVDIRDETGGEGAEASEDNSTSQQQRIVCEIEHFAESEMGPLFLATTILDMIPAMLHVSDSDQCIVYCSNNDYVQRVLHINGEHFLGYGWKDLVQPGNSLRNSESHQSQISLRVTESEIVAPDGTVVPVRTMSLPFKGLYDTEFWIGITIDRELEHTLAQQKEALDNALKEARHAAGMKDRLMANVSHEIRTPLHGLLGMIQLLQNLDNLTPEQSECVEDMTQCXVALKQIIDDILDLAKIEAGKYTIHPTPTNITEIAQSVVNSTRPAFSSKEIVSSVHVSQEVPSRVLVDGSRLRQVLYNLVVNAFKFTERGTVELMVNTVGEMRRLHSDDSNMEGMVDTDLLFVVKDTGCGVPYASQAELFKPFSQVSFTSSPSHRGTGLGLAISRQLVELMGGRIWLKSTEGQGTSLAFTIKASVAKRRKPLTRQSSTPQNVGQQPKTSEKSRHLLEAPFPLRILSVEDNEINQALMRKFLGSMGVQAVAAMNGKEAVELVAKAADKNQHFNLVLMDLQMPILSGLEATYAIRNTLGIPPDKQPYIIAVSANAYEHDIVQGRNAGMEDFVAKPTILGALRQALEKAISELAKRRLNGPLTPMPDEAL

>[GpHHK10](http://genome.jgi.doe.gov/cgi-bin/dispGeneModel?db=Ganpr1&id=501874) Ganpr1|501874|

MQARHQELRARNAELKEDLSMLRASVGKSDPVEWSRITLEHVTETTPCESHGGNAFSKSTLNSEFDGGRDAPLVKAQYKKSFDVFRCCYSNQPVRRVDQTLADSFYNLIKELVDAVSATITRSDMDNNTLFALKNKYLQHLMQCSFDDICRKGWIEYLSVVCGASAPIGDGSNIGLWNCKDGTSVNRHFENELSLQNIELACKLQCAESASCAKDWLVAIVLHELRTPLNRMMGTARLLQESESLDADQIEQLDALMESGEALKTVIDDLLAYSSMELGKARLIPTPFSLRSVCRSVVITFRASTLKKLGVSLSLHIDDSIPDTLMGDAVRPRQVLINLVGNALKFMEEWSVTVVVCVAGLLDGESGADTGRGIPPELQDRLFKPFSQVDQVSKYSGTGLGLSISKQIVKMMSGRMWMESAGRRGDWNDSTAESRPRRPGARPPPFVLSPQSPLYALDPPLRVLLTEDNPMYRMVATKFLAKLGVRPDNAHDGEEAFDMWERGCGDGKGYGVVLMDGQMPKLPGLEAARIIRSLLPHLQQPYIIALTANVTEGDIAQALCAGIQEHLGEPVTLEDLMRVPERAVVEVRGGAA

>[GpHHK11](http://genome.jgi.doe.gov/cgi-bin/dispGeneModel?db=Ganpr1&id=129927) Ganpr1|129927|

MSNTQDSGRPNLLRRINTSPPHQYAGRRACPGSPASLRSAPPHEQRNPYPASRVVTFVNPVQRYSRLHERRSFDRPDAAPGSGGEGNRSPTEFERLMKELQSEREQRRKAEERARWMEAFCDQTPAFLRCVMDVVENPDGTNDAIIVWASNRMIQYRYGGRDPAGLSLKKELQQSWHSVASYFVGKCLEAAASPNSTLEFDIEVPVTEVDDHPPRSPSECSAASDATVESAGFGTLEYSRATGDNYPVSASFTTLTALGAEGKSGGVYPGSSTITRVKRMRELVTFVETTPTGAKRIAVSAIDPHEEPLLQLVESRYKRIMDLVPAFVVVCDVEADSKPIIWNANSYVRDQLLGMDPGHLLLQHSKYNPTFFSIVHPDDREHAKKAFSLDVLKLQGGTGDSVSFRVTKPSKDPNPTEVDAGNVVHVKARSALMNEQGQEFLVLVITDAELEYQLHLKNSDLQDALSSKELFIANVSHELRTPLHGLKGMTRLLQETSLRSNSEGVQASSPSDSDVSSASSVLPFRRANSPLSPVSSTFSKEQEEYLHMIRTCTDDLTRVIDDLLDFSKLQAGKTQLDAACFNVRDLLNSVTSLLSSTATERGIFLVSQVAENVPKIAKADPGRIRQVLLNLVGNAIKFSGSGTTVSIALRMDPTHQDARITSAFKMLFEIKDHGIGIPVSRFDRLFKPFSQVDSSTSRNYGGTGLGLSISRGLVELLGGRIWVDSEEGIGSTFSFTVAFARPTPDEDLKWLEEHGESGPDLGLTLPISRDTENIVGSVAPMVAPSTAPNVFEGVIKSARDDSVNPTTRTRRTKLPIRPLPKTRNPDLQLLLAEDNLVNQTLVARLLEKSGFRNVEMCLNGAEAVKTVSERGKNGIPIDLILMFFGIDAGGGDVDTFVDIQMPLMSGYEATKLIRKMDLTVQPWILALTANAQNGDKKRSIEHGMDGHLSKPLELVDLVSAIDAFGEHRRRTCE

>[GpHHK12](http://genome.jgi.doe.gov/cgi-bin/dispGeneModel?db=Ganpr1&id=50552) Ganpr1|50552|

MDAGTHGAHSRTSSNQTSDPSSTVGEPGGAQSVIGALPPNYHALLSASAMIYGTPTYVEMRHNLREKYKDFRKPSWPVFDDNARSLLAQFETPLWVWDFNKVGMEWANAAGVRVWRKRDLQHFLATDYRTDLTDATQARLIQIEDALLRGEKLYEQWTFYPGNIEGKRMAVTIDCNVSGVFDTSGTLFFIIEGIERAASTVAYGTQSTPGESVPFPIQADTATAIDTDAANNNGGTKRSRGSESEVSDTTDKRGQAPPSDNEMSPMEKKRKVAITTQFENDAGHNETGGASPKHSSSEVVSIPPTTQSDIDGHRITRPINTSYSTFHATVAPNVLRSVESFRHAPYCVLVLSPEYRVLVEHSLTMALFGTKFDLWSAFVDQQDAERARRALSSPSLQNGVFVDEVRLRTVNGERWFRMDGKRMNDPVTGGVLVLVYLVDVSLQKELERQLNNAKEMAEAGNRAKSEFLANISHEIRTPLTGILGYALLLHETSLDEQQRELLRTIRTCGDDLHTILSDVLDWSKIEAGKLELDPRPFDLVDLVRGVMELLRVRARERDLTMSYTVRLKDGQEVVNVMQKGSGVCPRSNRNRGSASTVTPGSPSTSYALLPSAYSPTDVPTVVLGDEQRLRQVLINLTSNAIKFTAPGGCVDIMISSTEDISEHDRDSSWWLDSYLPDDDIMASRSAINDSESTQSFTTNVWTKSRVKRQRVTLAVKDTGIGIPASRMDRLFQSFSQVDASTSRKYGGTGLGLAISHRLVELMGGTISCTSEVDVGSTFQFEVQLGVLAATKAVVMSSQIEDGNGVAPPSSTVPENLPSSSVTVGRPTISADLSTEGQGSSRRKTGAGIPAEYAGLGKQISLRILVAEDNPVNQKLMIRLLDRLSFMADLAVNGTEAVQKAKQCALESNPYDLILMDLQMPETDGLSATRLIRANPLLTACQPVIVGVTANASVSDRDECLAAGMDGYITKPLKLEQLVDKIRICIERRTRT

>[RaHHK1](http://genome.jgi.doe.gov/cgi-bin/dispGeneModel?db=Rozal1_1&id=5218)(X-B) Rozal1_1|5218|

MPEIFEPILVDFSSSQLLFETEAFSFFRAIMYKALPNTKPIMRDESSASRVLIKTPSEELFDRLDEVDRIIPYFQNEKRMAERFASKCGMFHVPRWIRRTQEGIALVYDDFGGCSLREMCLKMGFSDRLRVFLKVAIDITRMLYVLHDSGGVFRNLSMKCFLVDDNFDMRVFNYTLMSFVESESVSFDFIDRWESSMIDLQYVSPEQTGRMNRCVDYRSDFYSLGVILYEWLCGTAPFDGADRHDLVYAHLAVQPRHPSEVWNEIPKAYGDVVMKLMSKRAEDRYFSCSGLLVDLESLTRGVDKSFIAGVYDDKSRLMIPEKLYGREEQVKAIIEAYETVCSGQVAVVMISGYSGIGKTSLVPFNGLIKHLLSLSTELFEKWRDKILEALEGHGGALVEIIPGLIDIIGEQEELPKLGPEETTKRMSLALKKFVRVFCSVEHPLVLFMDDITWADAESLKLMEELANLHGIALMLIGAYRDNEVPVTSLLNEVLGRLRHTDHYLFEIVLEKLNVNDVQLFLSDCLYYHMASPRLCKLTQLLYSKSGGNPFFLIQLLKHLVSQGTIYFNFEIGEWEWTVEDLEKVEITENVVYLMIKLIRGMKESNQRILKCASCCGTIFKSRDVAKCLGMSSYELVGELDEALKNNLVVKVNGCEDIEATAVEINTDKFDNFKYAFTHDRIQQASNEMLKEEEKRNIHLKLARYYYEECLNEKMKEEALFGIVQHYNEALDLVVEDEERLIVCELNLRASKRARASASYSTSLTFSNAAIRSFPDKLKKDHFEFYFKLLLNNLESEYLNTNYERAKEMIDLALQESQSELQKAQVLSHQVLYKTSQGKVLEAIESGWQAFRLLGVEIPTESGAIDRMFNELFEKNRMLSKDIEKLIELPEMTREDYRVVIKIELTKVNNPLLFEKGIDLIPPVYFARPELLVPMILKLVELSVKNGISSLTSYVYCLFGLVLSGLKYYKDSYVFGKAAMHVLDKYPNDPIECQVIKVYASHIQCWNEPLMNTLETFNRSLQAGINYANKEYYGYGSAERIIYSFFSGVKLELIMEQGSRLLTIVEELKQDIGIYYTRLFLQLIYNLINSESVIDPIILTGQYFNESDFHKLESVTLLVFAFYLCKLILCVVFGEYDKACIYSALAKQNAKGAPGLFMLAELEFYATCGFIMEYRNNKEFLIDDIENGLNELKVGMNSCEYNLDNKIKILEACKAAIIGDSNNSFKHATHSNFNHEIDQHEAERVEHLFDLAIDASLKDGFIHEYAICLELKAKLLMRSKSNYYLLHQEAYYAYLKWGSTTKGHQLLNLIGKRLLSTTPRLNSSTVSEKIERRSIVEINSSILIKACEVINDLVDFDSICRTLMELVVKCVDADRGYLVMQDLINNVDPHVKLSIVGKNSKGENGKGEKSSSSSVSDGSSSSNIQMHPSTCTSSTSTSTTSMVPWSLINYCLRSKKKVINEFDDFIKDEYFNLNKTNHVLCVPLVDKARCIGCVYLESNRIGFKQEQIETLKLLLFQTTTHLVNSMLISGNEKKDLMIEKLKQDLRKNNTNLESLVQNRTKILEIRNKQLTDEIQERKRIEVELKRAKDEAEAATLSKSQFLANMSHEIRTPMNAIIGFTDLLLLTNLNSNQAEFASSVKSSAQDLLTIINDILDFSKVETGNIVLESLKFNIEKSIESCLEVVNSIKNSENLDLSLIVNKNLPNFVIGDPTRFRQVVLNLLSNAIKFTKQGDVCIQVEICENEECEDELAIGNLYEVELTLGNQLTDAQSCFALNSPLPSVKSNNSHSSQPSIYSKQFHSTSESFHSSNPTNNKYLLVVKVYDTGIGISKENRKKLFKLFQQADSSITRNFGGTGLGLSLSKSLANAMGGDVYIASSKVNVGSCFVFKCKVGIDFSSSTSLISDSTSKSTSTSSTMNILNNMTLNTSTPTMNIPSTSTMNTPSTPSTMSTPSTFTFTSSTTSTSTSNESNDNDVLNFNLLLNKKYSLIVDDKSSFFSKSLIYYLENTFNCIPNDKTPDFIIIKDSFLLPTNIPSTVPSILIKSKKSLHKSAFELTQPVKRKALYETIIKALKINPKNNLQSIPESIPEKSQSKEKTISDVSILLAEDNLVNQRLIFHMLKKIGYKCDLAENGVQVLELLEKKTYNIILMDVQMPVMDGITCTRIILKKNLKNRPLIVALTANALSESKERCLDAGMDDFLTKPIKLSELENVLSKIQSTRK

>[RaHHK2](http://genome.jgi.doe.gov/cgi-bin/dispGeneModel?db=Rozal1_1&id=3629)(ETR) Rozal1_1|3629|

MNSELTPLNITEIVSDVLISLSYFAIPLEIFYFQKLLPNPFKYRIVIWLFEMFIFACGTTHFVGSFKFSYNVASIMVLTKVLTAIVSFITAILLIRVFPLAFSLQNRNMKLEEEIGLREENERELIKANASLLRFRRIVNRIRSSLDENILIDTAVREIRREFAADVCLIAQKDSLAVAEGTFKVRGISFKTLKGSIDTLSLNSLNENKDASFMNKDEIRGIFGLDTSVKSGLFIRLKDSQNCELGIMFLLNYCYVNKFDEIEMELLHEVAIQISIALDQAISIEKERQSVENLKKEFQRNELLNKEKNAAEMTSKMTADFLATMSHEIRTPLNAIIGFGNILQQQSLTEEQQEILEMITSSSNLLLNLINDILDYSKIESGFLSLEDKEFSLVSSVERVIMLNSQHSFSKNIQLNMFIDPSVPIMINGDETRLKQVLNNFINNAIKFTDTGEVIVTIATTEDYFKFYDFSEWKEFHVLAANNHNVPRLYFRIIDTGIGIAEESLPNLFQKFKQVDSSITRRFGGTGLGLAISFKLVEIFHGSLSVSSLVGKGSAFSFSISLGSNPGKKWTENPAFSKKFSVAIVSPILRTRQSIEHILTCLEYPFKSFSSISEYSKSTDTYRIVLVDETVSESHLDNITIPENCMLLVNSSSTIPVTFKQRFVACLTKPLRYTNIIKIFSSINNGNSISLDKAAHLSRSSLFEKKLHILVVDDNAINRKVIAKLLESILCPQIDFAEDGAVAVKLALSKSYDIVFMDVSMPVMDGLTATRLIKKEKKVFIVAMTANALYQERLLCMESGMDDYLAKPVSRDDVISLLERFLKRL

>[RaHHK3](http://genome.jgi.doe.gov/cgi-bin/dispGeneModel?db=Rozal1_1&id=4039) Rozal1_1|4039|

MFPNIERNISPTLIIDKKLNIKSLSNSFYTKLGIDTSVIFRNLKDHLKPIEPEIYGVKYLNDLTYLKKTDYSVLDFNGHELYHLEMEEDFFIIEVQNVYCCLKSLILLERCTSILKKSGIFITNHEGYIEMTNSLADSMFKVSDISESEDKNVLKFFKAEVKEFIKQVKETGKPKRQMRTVAYVPDGQTMTTDFDIDGFEICGEWTFVIRVTDTRSHSFGELEFQSVVDTAVDPILIANSTGIIQFVNKATVKNFGFEESELLGENLNMLMPSPDKEKHDGYMLNYRRTNKPKVLGIGREVVAKRKNGEMFPAELAVSEFIHDDEIKFTGILRDISEKKKAQQRILEAEKAIYKTEAEYKAKSEVFAFMSHELRTPMNGVLCMTTMLRDTPLTQEQSEVVDIIRASSENLLHVINNILDLSKIDAQKVNLEQIDFDLHSVILSVLEITQPTANKKQVETFLDYPNNLSLIMNGDPTRLRQVIMNLVGNAVKFTDKGHVCVQAYKEENKLKIIVKDTGIGIPESDLPHLFDHVYQTKAAVTRSHGGTGLGLTISQRLCQLMDGSISCSSLVNQGSTFTVEIPWKDPLKFVDTIFSKLKISKIVLFHSNSLVSPQIVSDFVKCGCVVLDLLLLHPNEIDSDVDLIIIDVNTSGIERLLSDDWKIIIEKHKAKIILMHNYDTFIDWKQFRNMGLNFVIRKPYSLPKILSIVAKMQSVELKEISLFNSNQLKISTIFTSAEKDILKRLKVLVAEDNDINQTIMRSFLLKFGINQVQMAGDGVEALELFKRSKYDLVLMDLMMPRMGGVEAAKLIREYELLKQLEKTIIIAVTADGHSKFRTPLCFIRFSYSFGFESSKRQNALYLNENTVITSSGSSLVFLDPKNLSQDHVSLDNDSSISCICTDSTKSYYAVA

>[RaHHK4](http://genome.jgi.doe.gov/cgi-bin/dispGeneModel?db=Rozal1_1&id=3104) Rozal1_1|3104|

MISSLLQKENVNIDLILKVLKPASPIQNTCTYGSCTLSWFNVDATAVLDNENKLMYYVVLLHDISRQKQVEDEQRKANRLKTEFITTISHELRTPLNCIMGSCSHLLHLINSNPGKTLDQIFQFNDFCETISVTMSSSQFLLTLVNNVIDIQKIEAGKMEAQNITFDLPGIIVKAVRACKPIASQKKIRILTFFKKLYCSTSKVDCNDSSIVCIKKFNIENDFENITVSLQALKGSEMDSTDLLPKYCIGDPEKLFQVVMNILSNSVKYSPSNSLIKVHISSFLYKSKTILTFSIHDQGQGISPEDYKKLFQRFSRLKLRKRSDKPASGSGLGLNICKRLLMIMNGDIWVRGNEDGIEGEYKELAELILKSEEDSVHEKWIKQYCKGYPEKFEGACFTFVVPVECAKENDTTCIKSKDEVDNSAILTRTLENLVFGHLRENQSIENINKSSVELDRNVCDSAFSLSASIVSLWNQLNLLVVEDNIINQKVLIKMINEILKNCIPFSIDVAENGLVALEKVDQKFYQVVFMDIQMPVMDGVEASLKICEKYPDCSKRPIIAAVTANAMKTDANQNIQRYFDYYLNKPLTITKIKSIFSKLGLPF

>[RaHHK5](http://genome.jgi.doe.gov/cgi-bin/dispGeneModel?db=Rozal1_1&id=946) Rozal1_1|946|

MNTNEDWRFRRNPYTACADGIKFYVGVPINVPSEYGKGNCRIGTFCVIDRAPHIGEIPSAFKRLLFMFADIGSFELLSFTSKIKEEREKTFLSSIEKVMMKSQPQNSYSICDPMISNISSELGLSCFLLMYNNSCELELKFCSAAFSLAKSKRKFWSYLKYKVPIFLCGENFERHSDFNFDTLPECIFFSGCGVVLKKYDGTIYGVLLAFSSFETRPVTHHDIKLLELWTVRLMQALRSFEKISELQKFNESMKETMIFQDEKLKTLQKGLSSFSHEMKTPLYGITGALESATDCIECRELLSIIKECSETMKTVVNDLIDSQSLRATKSSLTFHAVDVGLAFLLKKVLSVLNYNIASKSMNIKVCVRNDTIIHIDISRGKQLLINIIEFSDNGKTIEIFDDWSDSTFYKLIIRDQGIGIDEHDKDKLGLEFFQQSHNRGGLGLGLSICKNIVSLVNGYITLNNRNDGKLGACCTIALPDTAVKFCKVENPERWIYEENHMTNEKMGTIMDEIKGSRIAIVDDNAVCQRILEKQLSILGFSKISVFQSIEKILSQIQDKTVFDIIISDLLLLGSINGDVGSRLLRDLLPDCLIIIVTGLSRNEIGEHCADVVIEKPVSLEELRDALVRSHM

>[AtETR1](http://www.ncbi.nlm.nih.gov/protein/30697334?report=genbank&log$=protalign&blast_rank=1&RID=KY5RW1HM014)(ETR) At1g66340

MEVCNCIEPQWPADELLMKYQYISDFFIAIAYFSIPLELIYFVKKSAVFPYRWVLVQFGAFIVLCGATHLINLWTFTTHSRTVALVMTTAKVLTAVVSCATALMLVHIIPDLLSVKTRELFLKNKAAELDREMGLIRTQEETGRHVRMLTHEIRSTLDRHTILKTTLVELGRTLALEECALWMPTRTGLELQLSYTLRHQHPVEYTVPIQLPVINQVFGTSRAVKISPNSPVARLRPVSGKYMLGEVVAVRVPLLHLSNFQINDWPELSTKRYALMVLMLPSDSARQWHVHELELVEVVADQVAVALSHAAILEESMRARDLLMEQNVALDLARREAETAIRARNDFLAVMNHEMRTPMHAIIALSSLLQETELTPEQRLMVETILKSSNLLATLMNDVLDLSRLEDGSLQLELGTFNLHTLFREVLNLIKPIAVVKKLPITLNLAPDLPEFVVGDEKRLMQIILNIVGNAVKFSKQGSISVTALVTKSDTRAADFFVVPTGSHFYLRVKVKDSGAGINPQDIPKIFTKFAQTQSLATRSSGGSGLGLAISKRFVNLMEGNIWIESDGLGKGCTAIFDVKLGISERSNESKQSGIPKVPAIPRHSNFTGLKVLVMDENGVSRMVTKGLLVHLGCEVTTVSSNEECLRVVSHEHKVVFMDVCMPGVENYQIALRIHEKFTKQRHQRPLLVALSGNTDKSTKEKCMSFGLDGVLLKPVSLDNIRDVLSDLLEPRVLYEGM

>[AtERS1](http://www.ncbi.nlm.nih.gov/protein/OAP07328.1)(ETR) At2g40940

MESCDCFETHVNQDDLLVKYQYISDALIALAYFSIPLELIYFVQKSAFFPYKWVLMQFGAFIILCGATHFINLWMFFMHSKAVAIVMTIAKVSCAVVSCATALMLVHIIPDLLSVKNRELFLKKKADELDREMGLILTQEETGRHVRMLTHGIRRTLDRHTILRTTLVELGKTLCLEECALWMPSQSGLYLQLSHTLSHKIQVGSSVPINLPIINELFNSAQAMHIPHSCPLAKIGPPVGRYSPPEVVSVRVPLLHLSNFQGSDWSDLSGKGYAIMVLILPTDGARKWRDHELELVENVADQVAVALSHAAILEESMHARDQLMEQNFALDKARQEAEMAVHARNDFLAVMNHEMRTPMHAIISLSSLLLETELSPEQRVMIETILKSSNLVATLISDVLDLSRLEDGSLLLENEPFSLQAIFEEVISLIKPIASVKKLSTNLILSADLPTYAIGDEKRLMQTILNIMGNAVKFTKEGYISIIASIMKPESLQELPSPEFFPVLSDSHFYLCVQVKDTGCGIHTQDIPLLFTKFVQPRTGTQRNHSGGGLGLALCKRFVGLMGGYMWIESEGLEKGCTASFIIRLGICNGPSSSSGSMALHLAAKSQTRPWNW

>[AtETR2](http://www.ncbi.nlm.nih.gov/protein/OAP05469.1)(ETR) At3g23150

MVKEIASWLLILSMVVFVSPVLAINGGGYPRCNCEDEGNSFWSTENILETQRVSDFLIAVAYFSIPIELLYFVSCSNVPFKWVLFEFIAFIVLCGMTHLLHGWTYSAHPFRLMMALTVFKMLTALVSCATAITLITLIPLLLKVKVREFMLKKKAHELGREVGLILIKKETGFHVRMLTQEIRKSLDRHTILYTTLVELSKTLGLQNCAVWMPNDGGTEMDLTHELRGRGGYGGCSVSMEDLDVVRIRESDEVNVLSVDSSIARASGGGGDVSEIGAVAAIRMPMLRVSDFNGELSYAILVCVLPGGTPRDWTYQEIEIVKVVADQVTVALDHAAVLEESQLMREKLAEQNRALQMAKRDALRASQARNAFQKTMSEGMRRPMHSILGLLSMIQDEKLSDEQKMIVDTMVKTGNVMSNLVGDSMDVPDGRFGTEMKPFSLHRTIHEAACMARCLCLCNAIRFLVDAEKSLPDNVVGDERRVFQVILHMVGSLVKPRKRQEGSSLMFKVLKERGSLDRSDHRWAAWRSPASSADGDVYIRFEMNVENDDSSSQSFASVSSRDQEVGDVRFSGGYGLGQDLSFGVCKKVVQLIHGNISVVPGSDGSPETMSLLLRFRRRPSISVHGSSESPAPDHHAHPHSNSLLRGLQVLLVDTNDSNRAVTRKLLEKLGCDVTAVSSGFDCLTAIAPGSSSPSTSFQVVVLDLQMAEMDGYEVAMRIRSRSWPLIVATTVSLDEEMWDKCAQIGINGVVRKPVVLRAMESELRRVLLQADQLL

>[AtERS2](http://www.ncbi.nlm.nih.gov/protein/OAP16410.1)(ETR) At1g04310

MLKTLLVQWLVFFFFFLIGSVVTAAEDDGSLSLCNCDDEDSLFSYETILNSQKVGDFLIAIAYFSIPIELVYFVSRTNVPSPYNWVVCEFIAFIVLCGMTHLLAGFTYGPHWPWVMTAVTVFKMLTGIVSFLTALSLVTLLPLLLKAKVREFMLSKKTRELDREVGIIMKQTETSLHVRMLTTKIRTSLDRHTILYTTLVELSKTLGLKNCAVWIPNEIKTEMNLTHELRPRIDDENENEHFGGYAGFSIPISESDVVRIKRSEEVNMLSPGSVLASVTSRGKSGPTVGIRVPMLRVCNFKGGTPEAIHMCYAILVCVLPLRQPQAWTYQELEIVKVVADQVAVAISHAVILEESQLMREKLAEQNRALQVARENALRANQAKAAFEQMMSDAMRCPVRSILGLLPLILQDGKLPENQTVIVDAMRRTSELLVQLVNNAGDINNGTIRAAETHYFSLHSVVKESACVARCLCMANGFGFSAEVYRALPDYVVGDDRKVFQAILHMLGVLMNRKIKGNVTFWVFPESGNSDVSERKDIQEAVWRHCYSKEYMEVRFGFEVTAEGEESSSSSSGSNLEEEEENPSLNACQNIVKYMQGNIRVVEDGLGLVKSVSVVFRFQLRRSMMSRGGGYSGETFRTSTPPSTSH

>[AtEIN4](http://www.ncbi.nlm.nih.gov/protein/OAP06947.1)(ETR) At3g04580

MLRSLGLGLLLFALLALVSGDNDYVSCNCDDEGFLSVHTILECQRVSDLLIAIAYFSIPLELLYFISFSNVPFKWVLVQFIAFIVLCGMTHLLNAWTYYGPHSFQLMLWLTIFKFLTALVSCATAITLLTLIPLLLKWKVRELYLKQNVLELNEEVGLMKRQKEMSVQVRMLTREIRKSLDKHMILRTTLVELSKILDLQNSAVWMPNENRTEMHLTHELRANPMRSFRVIPINDPDVVQVRETKVVTILRKNSVLAVESSGCGGSEEFGPVAAIRMPMLHGLNFKGGTPEFVDTPYAIMVLVLPSANSRVWTDKEIEIAEVVADQVAVAISHASVLEESQLMREKLGIQNRALLRAKQNAMMASQARNTCQKVMSHGMRRPMHTILGLLSMFQSESMSLDQKIIVDALMKTSTVLSALINDVIDISPKDNGKSALEVKRFQLHSLIREAACVAKCLSVYKGYGFEMDVQTRLPNLVVGDEKRTFQLVMYMLGYILDMTDGGKTVTFRVICEGTGTSQDKSKRETGMWKSHMSDDSLGVKFEVEINEIQNPPLDGSAMAMRHIPNRRYHSNGIKEGLSLGMCRKLAQMMQGNIWISPKSHGQTQSMQLVLRFQTRPSIRRSILAGNAPELQHPNSNSILRGLRITLADDDDVNRTVTKRLLEKLGCEVTAVSSGFECLNALSNVEMSYRVVILDLQMPEMDGFEVAMKIRKFCGHHWPLIIALTASTEDHVRERCLQMGMNGMIQKPVLLHVMASELRRALQTASE

>[ZmERS1-25](http://www.ncbi.nlm.nih.gov/protein/821325025/)(ETR) gi|821325025| MDGCDCIEPLWPTDDLLVKYQYISDFFIALAYFSIPLELIYFVKKSSFFPYRWVLIQFGAFIVLCGATHLINLWTFTTHTKTVAMVMTIAKISTAVVSCATALMLVHIIPDLLSVKTRELFLKNKAEELDREMGLIRTQEETGRHVRMLTHEIRSTLDRHTILKTTLVELGRTLGLEECALWMPSRSGSSLQLSHTLRHQITVGSSVPMNLPVVNQVFSSNRAIIIPHTSSLARVRPLAGRYVPPEVAAVRVPLLHLSNFQINDWPELSAKSFAIMVLMLPSDSARKWHVHELELVEVVADQVAVALSHAAILEESMRARDLLMEQNVALDLARREAEMAIRARNDFLAVMNHEMRTPMNAIIALSSLLLETELTPEQRLMVETVLKSSNLLATLINDVLDLSKLEDGSLELEIKAFNLHAVFKEVMGFIKPIASIKRLSVSVMLAPDLPLCAIGDEKRLMQTILNISGNAVKFTKEGHITLVASIVKADSLREFRTPEFHPTASDEHFYLKVQVKDTGCGVSPQDLPHVFTKFAHPQSGGNRGFNGSGLGLAICKRFVSLMGGHIWIDSEGTGRGCTATFVIKLGVCDNTNTYQQQLVPLVWPSSADSNLSAPKVLPDGRGSVSLKSRYQRSV

>PaETR1(ETR) 10048g0010 Picea abies

MEVAIQTRDFVCGFRKGRCSLLSHSSHAIRTRRDCCNDLSGGDCHCFTPPPEHCNGIRGCEFWFIEMQLWVIVQFGAFIVLCGLTHFINIWTYGPHSFHVMLALTVFKFLTALVSCATAITLVTLIPELLRVKVREIFLKNKARELDREVDIVKRKEETSWHVHMLTQEIRSSLDRHTILNTTLISLAKTLNLENCTIWMPLADGTAMEVTHELKRRHLQVPLTVPTTDPDVEKITRSNSAILLNPDSALGKESNHHWSLAGPVAAIRVRLLKASDFKGGTPEHVAPSYAIMVLVLPCEDERQWSSQELYIVQVVADQVAVALSHAAVLEESQKMKAPLIDKNKALQQAKQDVLRASEARHSFQLAMNREMRVPMYAISALSSILQSASLNVEQLAMANMLAKSSSLLSTLINDIMDFSELEDTSLVLQLHPFQLHVMLKDAAHLTETMSRSRGLLLNIEIGDGMPDHVIGDEKRILRIILHMVGNAINSTKQGTISINVCVEDRAEGWWDPNNRRWRPSLCDGFTYLRFEIRTSGSGSIQNDNPSFLAVVQEGKSDSSTSTGEGLGFAICKKFVQLMHGNIWLEPNSKGEGSVVTFLIRVQLQTSTAKKHWHSPDEKIYKSSFIGLKVLIADDNNVSRSVTRRLLQELGCQTIEVDSGYRCLMTLLQSGSSFQVVFLEVCLSQMDGYEVAFRIRQKFRSRNRPLVVALTASTDKETMERCLQTGMDGVIRKPVTLREMSNELFKIIHQTNNIHE

>[PpETR7](http://www.ncbi.nlm.nih.gov/protein/168043876?report=genbank&log$=protalign&blast_rank=1&RID=R1T7BPFU014)(ETR) gi|162674261| MDTCHCVEPQWPADDLLMRYQYVSDFFIALAYFSIPLELIYFVKKSSIFPYRWVLVQFGAFIVLCGSTHLINLWTFSPHTRTVAVVLTVAKIFTAVVSCATALMLIHIIPDLLSAKTRELFLKNKAAELDREMGLIRTREETGRHVRMLTHEIRSTLDRQTILKTTLAELGKALDLEECTLWMPTRLGQDLLLTHSLRQLEQTHITVPIHHPVVKQVFSNHRAIMITPNSPVCLIRSRQGKYSMGDCVAVRVPLLPLNNFHTDWPESHSKRAYALMVLMLPCDSARRWHVHQLQLVETVADQVAVALSHAAILEESMRARSLLVEQNVALDLARREAETAIRARNDFLAVMNHEMRTPMHAIIALSSLLQETRLTPEQRSMVDTILKSSNLLATLINDVLDLSRLEDGSLELETRVFNLPIVFKEVMKLVAPITSVKKLKSELTLDGDLPEFVVGDEKRLMQTVLNVVGNAVKFTKEGSVTIHVILDRDRTDYQRSEPHSLREPLSQVESRSQRDHHLSLGEQHCYIRVEVVDTGVGLNPLDIPNLFNKFLQADSSPTRNYGGTGLGLAICRRFVSLMGGDIWVESEGIGKGTTVNFSVRLSLPEKPNEQDRQITPSPASVHLRTDFSGVKVLVTDDNGVNRMVTRGLLTRLGCEVTVVSSGSECLQVISQPGQNFQVLLLDVCMPEMDGYEVAIRIQQKFARHERPLMVALTANTDKQTRGKCLDLGMDGVIMKPISLEKMRMNLTELLERGSLTPETRRKA

>[SmETR1](http://www.ncbi.nlm.nih.gov/protein/302795193?report=genbank&log$=protalign&blast_rank=1&RID=R1TBP9UZ01R)(ETR) gi|300153128|

MDCNCMDSLWPWLPEDIYTKSQYVSDFFIALAYFSIPCELIYFVKKSAVFPYKWVLVQFGAFIVLCGSTHAINMLTMTSHSRTVAFIMIMSKILTAIVSCATAVTLVTIIPDLLSVKTRELFLKNKADELDREMGLIRTQAEAVQHVRMLTHEIRFTLVRDTILNTTLVELGKALALEECALWMPSRDGLELHLCRTLGSTNPGRVTVPVHHPSIQPVFCTHRARNIPATSPVAAIRPRAGKYLPGEVVAVRVPLLHVENFNISYWPEGGDVPYALLVLMLPSDSARTWHLHELELVEAVAGQVAVALSHAAILEESIRQRDTLIAQNVKLDASKREAEMALRSRNDFLAVMDREMRTPMSTIIAASSHLQDTSLSQEQRAMVDTILNSSNLLATLINDVLDVSRLEDGSLELEKRTFNLPATFREVLNLVKPVASLRKLAISLCLGSELPEFVCGDDKRFVQIALNIVGNAVKFTKEGSVSITVRLEKPESFQASDYVVPSEKHCYIRVQVQDTGIGVNPQDIPKLFNKFIQAPAVSSRKQVGTGLGLAISKRLVSLMDGNIWFESEGLNRGCTVSYVVRLAIPDAVDQVSYALQQVPPSSSPRTSVTGLRVLVMDANGANRMVTRGLLLRLGCDAFAVGSPRECLQLLAQPGRSFRLVLVDVYMAESDGFEVAVKIQDMFKRHERPLVVALTAAHDRQTSERCSSIGMDGVIFKPISLEKMRSALAQLVDKGSFFDDCYPRKLSS

>[MpETRb](http://www.ncbi.nlm.nih.gov/protein/1026775428?report=genbank&log$=protalign&blast_rank=1&RID=R1TG5B7G014)(ETR) Mapoly0036s0126.1
MDACNCLEPQWSAEDLLMRYQYISDFFIALAYFSIPLELIYFVKKSAVFPYRWVLVQFGAFIVLCGATHLISLWTFTTHSKTVASVMTVAKVLTAVVSCATALMLVHIIPDLLSVKTRELFLKNKAAELDREMGLIRTQEETGRHVRMLTHEIRSTLDRHTILNTTLVELRKTLALEECTLWMPTRNGLELQLSHTLRQQSPVPITVPIHHPTIKQVFSTNRAVVISPNSPVAVTRPRTGKYMIGDVVAVRVPLLHLTNFHIGDWPESTKRPFALMVLMLPSDSARRWHVHELELVEVVADQVAVALSHAAILEESMRARDLLMEQNVALDMARREAETAIRARNDFLAVMNHEMRTPMHAIIALSSLLQETEMTPEQRSMVETVLKSSNLLATLINDVLDLSRLEDGSLELDMRTFNLPAVFREVMNLVKPIASVKKLSVSLSLHPELPEYAVGDDKRLMQTALNVVGNAVKFTKEGSVTVNVCLERPEYLRDPRYPDFQPSHGENHCYIRVQVRDTGLGLNPQDIPKLFNKFVQADSTTTRNYGGTGLGLAICKRFVNLMEGHIWIESEGLGKGSTVIFIVKLALPEHVNDIDRQTTPAVQPSHLRTDFSGVRVLVTDDNGVNRMVTRGLLIRLGCDVTVVSSGRECLQVISQPGQPFRVLLLDVCMPDMDGYEVAVRIQEKFARHERPLLVALTANTDRATRERCMNLGMDGVVLKPISLEKMRVVLTELLERGSLVESLRRS

>[Nostoc](http://www.ncbi.nlm.nih.gov/protein/17135160/)(ETR)_all0182 gi|17135160|

MSELWTYFFNSTPFIPHGHCYLWQTDLVWLHIISDGLIALAYYSIPATLFYFVRKRQDLPFYWIFLLFSGFIVACGTTHIMEVWTLWYPTYWVSGFLKAITAIISVFTALTLIPLVPKALLLPSSAQLERANKDLQNEIGERLKVEAELRKYQNHLEELVTIRTNEITNANEKLQQQINERQHIVEILRESEERYRYLAEAIPQLVWTADANGECDYFNQNWCDYTGLNLEESLGSGWLAALHLEDLERSYETWSKAVETGALYENEYRFKRAADGSYRWLLGRGLPLKDHQGRVVKWFGTCTDIHEQKQILEERARLLELEQTARAEAETANRIKDEFLAVLSHELRTPLNAILGWSKLLQERKLDPTRTFQALATIERNATLQVQLIEDLLDISRILQGKLVINITKVNLVTVILAALETMRLAAESKLIQVLTNFPPNIGQVMGDSARLQQVVWNLLSNAVKFTPNGGKIEVTLDKMEGYAQIVVSDTGKGIQPEFLPYVFDYFRQADSSSTRRFGGLGLGLAIVRKIVEIHGGTVTAQSLGEEQGASFTVRLPILPEEDGSVTYQQHRSASLVTNSLPLEGIKVLVVDDDADSRDFLAFILEQEGADVSLATSALEVLQLLPEIKPDVLVSDISMPDMDGYTLIRQMRTWTAEQGGAIPAIALTAFARQYDREQALQAGFQLHLPKPLNAEEFIAAVVELVKSQHSIVSFH

>[Kf1](http://www.ncbi.nlm.nih.gov/protein/971510323/)(ETR) gi|971510323|

MAAQVSHRWTLFWALLLGALVNVVRAETNRKLLGAAGVAADTCHCEKVYWAIDDSFARWQLVSDFFIAVAYFSIPVELIYFVHKSQVFPFKWILWQFGAFIILCGLTHLIAMFCYGPHSYTIMLVQTTAKALTAFVSCATGVTLIYVIPELLSLKVRELFLRTKAAELDREMGIIRTQEEANRSVRMLTHEIRSTLDRHTILRTTLVELARTLGLENCTIWMPSCNGRMLELTHELERRLVNVPTLVSVTDPTVLQVVNTRSAITVPASSAIGVASSRKGVQGQDLAPVAAVRLPLLELAHFNGEGHFAAQEESFALMVLVLPSTSARVWRPHELEMVECVADQVAVALSHAAVLEESQRARQKLVEQNAALDAARREAESAIRARNDFLAVMNHEMRTPMHAVIALSSLLNETELSEEQRSMVDTVVKSGTVLSTLINDVLDFSRLEDGSLALDPRPFELHTVLREAENLAGPMARAKNLNLSVHIDKDVPACVVGDDKRLLQTLLNLVGNGVKFTEKGSVRLSVSVERPGRKPDPGEPAWQPRHEGGSIYVRISVQDTGLGIRPSDIPKLFKKFVQADSTTTRNYGGTGLGLAICQKFVELMQGQIWIRSDGIGKGATTTMVVRLGLQPLSPLVANTSGERASTTAHEAAALVGLRILVTDDNSVNRIVTRRLLDKLGCKTTLAESGERCLTELAKPGADFQILFLDLCMPIMDGYTVNERIRERYPPGKRPLIIALTANTDKKTRDQCLHQGMDGIVLKPITLNELRDVMCALFANSAGFTRLPAEGYGFKNI

>[Bm|388937|](http://genome.jgi.doe.gov/cgi-bin/dispGeneModel?db=Basme2finSC&id=388937)(ETR)

MSDHDTTSWVMMVADVFISMSYMAIPIEIFFFQRTLTLPLPMLYKSVLLLFEAFITACGATHLVTVWKPFANTEVALCVVKVVTAVISTLTAILLIQVFPKAFSLPARAAWLEEELGSHMRHEQSLRMANDLLLKFRKVTQHIRRTLDTNSVCQTSVFELSVTLGVGCAIYMKEGDTYVCTHEDYRHVNDHSLRHSDKNIFWQKIRISKENKMITRMATLSYACCLNKRDLSDMVGEGVNLPFKAAMGVRFKIDGKDSGFVLIYSSEETWSLPEQEVELFEDIVGQIEIALQQSSQLSKEHGWKSPSSKLTQSIETPGHADERGLGSLRQSAAEATLPSSSPPPFSNHGQEYEVYDIEPKYWH

>[AMAG_07095](http://www.ncbi.nlm.nih.gov/protein/909135888/)(ETR) gi|909135888|

MIPITRASSGSASALAMTTTSTTMALAAALVSPLLLLFVVTAGPHHATHALPVPTPDPAPGILAASPLAPNRVVSRASWHIDPPLHGPPRDPTGDPTATVWVAVSMVSDALIAFAYFAIPAQIFYFERHLQVDSLRGRLRAIVWLFEAFILACGTTHLLKVWTTSDTATLALTKAVTAIVSVVTSVALVQLMPMAMLLPGRLFLLEEELVVRAHNERVLQAENTNLTKLRAITLCVRKALEFHTICDIASVQLARHFDLVGCAVFALDAHCPVPDGPATPAAVAAALATALRSAPPSPAISHCAWRFSPVPGHVPLADMPAAAPDTHPWAQHVALGPATCVALHHTPEYADLVAAQAAAAAAASPRPSSSSNDSSSRTSTGSAPRRIRAWTRFMQRRSSTDLAATDEHTSPAPPPPTAPWSAPATPVVSAPIPGAAAATTASAPPGTLRHDDMHSPPPLPHARDMTGSTAVSGDSLATGASASTVLPPPRTPLAVGARVDFAHVAAVWSRFPQRVVLDQPAVAAILGESAVPYATLVALRAQDRVVVVALLHRDRPVGENAILHDALGQIEIALDQAVQIEYEAKRQAQMSVLEREKQEAEALNSTKTVFLATVSHELRTPMNAIIGLVDLLLTKYTLSRDMREVLEIVAVSSSTLLNLVNDLLDLSKLECQGNSFTLDHRPFSVLDVVEKSIEVVCTQAEAKGLYLCAHLDHEFNVVVGDKLRLRQVLVNLMSNAIKFTERGGVTLCVTTEEPSMLVDEGDGESPTSATSTPKTGARSERRSKRRQRILDYRQPAKATADKDRAAAAAASHVRRSSLPAPPLPPLPPPAPAAPSLSSDTLVPAQADTVPPPPPAAAAADHGRDHGEADETVLPKRSVYFQVRDTGIGIEQDNMHLLFEKFQQMDATISRRFHGTGLGLAITSRLVGLHQGRIVVDSVLGKGSVFTVELPFPVAQVAAGDAQSAPLKTPPHDSAVGAVRHAHPPPPDPVPPTELVKPSLLLSEDQRPPRTLHAPAPSIPRSPRVRHPTTGSTGIPVAVADTCAAERSAIAALLRRMGCNPVPVDSTESLCAFFESETHEPGAEAPVIADAAVRVAAAVVEDWVAWSLDEPTRRALLDHKIPVLVTAHIQLASKERATDTALAAGRTRKCKVPFRFGLSKPIKLRALHRLLRDALEECYTPAELAAAGCAMPALDPQDMFTSPDDANTDVHADSAESAAPSTAAAAAAGPDPARIPQLHVLVVDDNAINLVVAQRTLASIGVAQIGTAADGLLAVQYVDAHPDVDVILMDVSMPVMDGFEATRAILAKGARLVAGGELRAPFPYICAMTASALPEERNVCLASGMHDFVPKPARCLDLVRVLERFVEWRDAFCPGMPVVVAPQLVGAHATVAAHAALELPAPLEPIEEVASEVASVVASVKDDAGEEMPGGGGEEERVGGDRSGGTTEGQG

>[AMAG_07058](http://www.ncbi.nlm.nih.gov/protein/909135254/)(ETR) gi|909135254|

MEVAGIVSDALIAIAYFAIPCQIVYFSRFIRLEGRGVQFKSVIILFELFILLCGLSHLIKVWTADNSWLMTIVKVLTAIVSITTSALLVKLMPVVLKLPERLFVLEEELGLRIQNEQQLQAENSNLQKLRSITQSIRRTMHFQSICDIACIQLTNHFDMVGCCIFALDLPGTMTPNTAASPNSPTTPVNHATSPTSNGNNSDMALCLAEYSRLPPATSLSASSPSTGSSPPPSGTHLVVNPSGSSRQGKMTTAMSMGVLAALNAAGGGCVGVGATGVTKGMRIDLSRLVATWSKTGEWRRISATTMHGVFGISLPRSPTNKSRATLTRADSVPENPLGSGDGVALAESYKGKKDDDDEEIEIRVLATGRMDGSSPTSDRHGDANVIPEARYGTLAMMRVQGRRLIVLMMHEHEDVHASPGVVQDALGQVEIALDQSLQIEQEASRRSQMSVLETEKREAEALNGMKTVFLATISHELRTPMNAIIGFVDLLLTQYELSRDMRDILEIVSVSSSTLLNLVNDILDLSKLEFQGKQFTIEEAPLSILNVTEESVEVVYSQAERKSIKLNAILDHCVDLVLGDKLRVRQILVNLLSNAIKFTSQGSVTVTVSTKMPNTFLTLPPDHPDFARLMVPRKQRVLDYSNANVKDAPAASNGPRTQIYFQVCDTGIGVEQDKMHLLFEKFQQVDATIARRFHGTGLGLAITSRLVELHRGRVWVDSLPGVGTLFTIMLTFTPAPANVTQVPDEPAAPSLLSAASAHGSLAASADLSATSSTAPTIRIAVLDSCPIETRAIATLLKRMGCEAAAFESVAALVAAVEHDRAFESTRPLSAIVTEEDFVRALTGDDSQRLATLGLPVVTLVRIKSSSRMLIGEGLYRHTATKPLKLRTLEATLRGALEYHHNQWRRAQLGAGDSVTASQMASRDGLAKTVSAPDREAALLEVGVEPGSQAVSEAMLVPVPDAAATAEVPSGAALLAHNVTQVEAAAYEVPTSATRVAGGDSGPNSGHASPKTAAPRPLCPKQVDMLRRAEGGARPAPSTAHSSSTGLSPAARPEGKLNVAGAPVMNRSLASLAQPVLFPPARKSPIEAVRAGTAAVGSISQLIQNCGTGGAGDGGLRDTLSSLKVLVVDDNNINQMVAARTLKTLGIDKVDTANNGQEAVTYFETHPTVSIIFMDVSMPILDGLDATRAILARSSHAPPGQRRRAPFICAMTASALPEERNTCLSTGMHDFVPKPTRRQDLMQLLDRYFTWRAAQDEEEAKGSAPLGMGLLQPFLYVPPSSGKHDLQGQHP

>[AMAG_09825](http://www.ncbi.nlm.nih.gov/protein/909140918/)(ETR) gi|909140918| MRALAAAFTAVALVATIGAFGAAAAAATANANANPTVLHLGGQGEWHFARSSQAGPNAPPPPPSPPSPPSPPPSSAPVSSSSPPSRDGTASNGGISSISSGMSTLMTVEMVADLLIGLAYVAIPCMIAFFQRQLVGATSPTYRGLLVLFQAFILCCGATHILHALRFSSDSILLVVTKVITAAVSCLTSAMLFWVVPVAFSMPARLHRLESELGEMIREANRRQYGVHDGARRPLRELTARLRHEQLAESPGVDGAGGGANSAAAGGSSSNIATTTPNAEDHHVDSEDEYKALPLERVCHLAVRAIAQSVPGFASVLMFMEPARDSVAPVAAPAELTTSGAGAGDDAVQPDWMGPGLRCIAEYHPAAPSSSARAAGERLVLAHAPFRLRTCIEACLDLVAPRLEHVDVALNYILDADVPEVVAGDRTRVTQILANLLGNAAKFTEQGEILVIVSTRPRRVVDPAVFTSVRKLDFRNPAQGVTPATLVAVSPESIDRAGRPVESATPAEGDRKSSLSPGSRHREATSASSASAPDPLSPRLATQSRTRTNSLGGHTGYLYFYVVDTGCGIHQHDMGRLFERFQQGQQGAARRQHGTGLGLAVSARLVELHGGQMAVQSAPGAGTVFSFSLRMQYAGAAHAGDHDDVVHAAAVAWEPLWQAPAAPLGSPWTGLELVVVPDPRLASSARSLGSLLDAIGCAYTCVPTVAQAQRVARRLHAAHVEPIHVAFLVVERALLAPATAPGSATDGGRGRRQELRAELLALQAIGPIALLTRRGRGDELTVDVTWFDYVLRKPVKRLSLERVLHKIADKHAQSSDAPPVVTDDVAAPLPTVVPTQVSVAVPVAVRVPDANSPAIAAAAAGAAAGGFPAAGAAAAFPGIQRHDSVMLHSVLDRPFVADFPTHRSPAAAGTPPAVAPFPLPPLIEPMATPSPAPSTPTSITTSRTTATPEREDWMRQAVLVLDDNVINLKVAQKLLKSIGVTAITTANHGGEAVDMVERGAFDIVFMDVSMPVMLG

>[AMAG_07677](http://www.ncbi.nlm.nih.gov/protein/909136656/)(ETR) gi|909136656|

MLVLFQAFILCCGATHILHALRFSSDSVVLVVTKVITAAVSCLTSAMLFWVVPVAFSMPARLHRLESELGDMIREANRRQYGMHDGARRPLRELTARLRHEQIAESPGVDRASSGATLAAVHGGSNSSSTAPLVQDHHVDSEDEYKALPLERVCHLAVRAIAQSVPGFASVLMFMELAHDPVAAAPAPAELVTSGPGAGDDVVQPDWTASGLRCIAEYHPAAPNSSARLFADHIVDTHDRIYCSMAPGRASRFSGVDLACLIGCTRFVNAIAMQMNYGSRRGLLVLCATKEGPVISSVNEILHYEDPLHYIQTALDLAHARDRTRRLRHELSAANSARTAAEAARTSALEQVVATNDLLATTSHELRTPMNAIVGMVDVLLDESSNVPLCHEVRESLEIVLSSANLLGNLINNLLDLAKLHHQGTAGEALVLAHAPFRLRTCIEACLDLVAPRLEHIDVALNYILDADVPEVVAGDRTRVTQILANLLGNAAKFTEQGEILVIVSTRPRRVVDPAVFTSVRKLDFRNPALGVTPATLVAVSPDSIDRAGRPVESATPADWDRKASLSPGSRKREATSASSASAPDQLSSRAASQARTRTNSLGGHAGYLYFYVVDTGCGIHQHDMGRLFERFQQGQSGAARRQHGTGLGLAVSARLVELHAGQMAVQSAPGAGTVFSFSLRMQYAGGAHAGDHDDVVQAAAVAWEPLWQAPAAPPGSPWAGLELVVVPDPKLASSARSLSSLLDAIGCAYTCVPTVAQAQRVARRLHAAHVEPIHVAFLVVERALIAPATAPGTATDGTRGRRQELRAELLALQAIGPIALLTRRGRGDELTVDMTWFDYVLRKPMRDAPAMPATDVAAPLPTVVPAQVAVAVPVAVRAPDASSPAIAAAAAAAAAAAFPGIQRHDSVMLHTVLDRPFVADFPSRGSPAAATPPAVLPPLIEPMATPSPAPSTPTSTATTTRTIATPEREDWMRQAVLVVDDNVINLKVAQKLLKSIGVTAITTANHGGEAVELVERGAFDVVFMDVSMPVMDGLEATRRIRHARSTQAAATVSPWICAMTASALPDEQTACQEAGMDDFVPKPVKRDAVIRALKNAHDHLQHRDRGSDAP

>[SPPG_07928](https://www.ncbi.nlm.nih.gov/protein/XP_016604759.1)(ETR) gi|907090295

MDVVLLLLGFLLERAWALPVPVESSGQIGAQTLPDVSTKLTVPWADAVIGASYFAIPVELAFFVFKLPSTTLYQKCVGGLFVAFILFCGIGHFLDASHMGVEWVIADRYLTAGVSAITAIASPYVLNYSVEAIKGFSEESKLVEKQRDMLADAQALTHLGNWELREDEKTGVRWINASDEFFRIFGMEPDTYEANRITYDRYWKCVAPEDREKIDKAVSEALLNGDSYHIIQRIRRESDRKEVYIRGYGKPVIRDGKIQGLRGTAQDITSQVFAEMELTRAKEEALIESKHKDVFLATMSHELRTPLTSIIGHVELMDETPMDETQKEYMSNARRAATTLLSLINDILDYSKLTAGKVDLDLRVTSVAEILDDVRAISKDLGKEVTLRVDSYDGPNVIGDSTRLKQILLNLVSNAVKFTMPGGYVFVTNTWTVDNADRVNLTISVRDTGIGMSEQVIQRLFNPFTQADASTSRRFGGTGLGLSIVKKLVNAMDGSIEVTSEENKGSTFTVLFKLPKAHVLPQEVPVPDKMGSMRILVAEDNKVTQSLLKRMLRDFTVDVADNGRQAVDMVKKNESYHMLFCDLNMPVLDGLEATREIRKTTQGREIFIVGLTANAFKTDRENCLKAGMNDYLSKPFTKLSLLAMIAKAKLSG

>[AtAHK4](http://www.ncbi.nlm.nih.gov/gene/814714)(CKR) At2g01830

MRRDFVYNNNAMFNPLTTHYSSDMNWALNNHQEEEEEPRRIEISDSESLENLKSSDFYQLGGGGALNSSEKPRKIDFWRSGLMGFAKMQQQQQLQHSVAVKMNNNNNNDLMGNKKGSTFIQEHRALLPKALILWIIIVGFISSGIYQWMDDANKIRREEVLVSMCDQRARMLQDQFSVSVNHVHALAILVSTFHYHKNPSAIDQETFAEYTARTAFERPLLSGVAYAEKVVNFEREMFERQHNWVIKTMDRGEPSPVRDEYAPVIFSQDSVSYLESLDMMSGEEDRENILRARETGKAVLTSPFRLLETHHLGVVLTFPVYKSSLPENPTVEERIAATAGYLGGAFDVESLVENLLGQLAGNQAIVVHVYDITNASDPLVMYGNQDEEADRSLSHESKLDFGDPFRKHKMICRYHQKAPIPLNVLTTVPLFFAIGFLVGYILYGAAMHIVKVEDDFHEMQELKVRAEAADVAKSQFLATVSHEIRTPMNGILGMLAMLLDTELSSTQRDYAQTAQVCGKALIALINEVLDRAKIEAGKLELESVPFDIRSILDDVLSLFSEESRNKSIELAVFVSDKVPEIVKGDSGRFRQIIINLVGNSVKFTEKGHIFVKVHLAEQSKDESEPKNALNGGVSEEMIVVSKQSSYNTLSGYEAADGRNSWDSFKHLVSEEQSLSEFDISSNVRLMVSIEDTGIGIPLVAQGRVFMPFMQADSSTSRNYGGTGIGLSISKCLVELMRGQINFISRPHIGSTFWFTAVLEKCDKCSAINHMKKPNVEHLPSTFKGMKAIVVDAKPVRAAVTRYHMKRLGINVDVVTSLKTAVVAAAAFERNGSPLPTKPQLDMILVEKDSWISTEDNDSEIRLLNSRTNGNVHHKSPKLALFATNITNSEFDRAKSAGFADTVIMKPLRASMIGACLQQVLELRKTRQQHPEGSSPATLKSLLTGKKILVVDDNIVNRRVAAGALKKFGAEVVCAESGQVALGLLQIPHTFDACFMDIQMPQMDGFEATRQIRMMEKETKEKTNLEWHLPILAMTADVIHATYEECLKSGMDGYVSKPFEEENLYKSVAKSFKPNPISPSS

>[AtAHK3](http://www.ncbi.nlm.nih.gov/gene/?term=At1g27320%5bsym%5d)(CKR) At1g27320

MSLFHVLGFGVKIGHLFWMLCCWFVSWFVDNGIEDKSGLLVGSVGDLEKTKMTTLKKKNKMWFWNKISSSGLKIPSFSYQFLGSVKFNKAWWRKLVVVWVVFWVLVSIWTFWYFSSQAMEKRKETLASMCDERARMLQDQFNVSMNHVQAMSILISTFHHGKIPSAIDQRTFSEYTDRTSFERPLTSGVAYAMRVLHSEREEFERQQGWTIRKMYSLEQNPVHKDDYDLEALEPSPVQEEYAPVIFAQDTVSHVVSLDMLSGKEDRENVLRARSSGKGVLTAPFPLIKTNRLGVILTFAVYKRDLPSNATPKERIEATNGYLGGVFDIESLVENLLQQLASKQTILVNVYDITNHSQPISMYGTNVSADGLERVSPLIFGDPLRKHEMRCRFKQKPPWPVLSMVTSFGILVIALLVAHIIHATVSRIHKVEEDCDKMKQLKKKAEAADVAKSQFLATVSHEIRTPMNGVLGMLHMLMDTELDVTQQDYVRTAQASGKALVSLINEVLDQAKIESGKLELEEVRFDLRGILDDVLSLFSSKSQQKGVELAVYISDRVPDMLIGDPGRFRQILTNLMGNSIKFTEKGHIFVTVHLVDELFESIDGETASSPESTLSGLPVADRQRSWENFKAFSSNGHRSFEPSPPDINLIVSVEDTGVGIPVEAQSRIFTPFMQVGPSISRTHGGTGIGLSISKCLVGLMKGEIGFSSTPKVGSTFTFTAVFSNGMQPAERKNDNNQPIFSEFRGMKAVVVDHRPARAKVSWYHFQRLGIRVEVVPRVEQALHYLKIGTTTVNMILIEQEIWNREADDFIKKLQKDPLFLSPKLILLANSVESSISEALCTGIDPPIVIVKPLRASMLAATLQRGLGIGIREPPQHKGPPALILRNLLLGRKILIVDDNNVNLRVAAGALKKYGADVVCAESGIKAISLLKPPHEFDACFMDIQMPEMDGFEATRRIRDMEEEMNKRIKNGEALIVENGNKTSWHLPVLAMTADVIQATHEECLKCGMDGYVSKPFEAEQLYREVSRFFNSPSDTES

>[AtAHK2](http://www.ncbi.nlm.nih.gov/gene/833552)(CKR) At5g35750

MSITCELLNLTSKKAKKSSSSDKKWLKKPLFFLILCGSLVIVLVMFLRLGRSQKEETDSCNGEEKVLYRHQNVTRSEIHDLVSLFSDSDQVTSFECHKESSPGMWTNYGITCSLSVRSDKQETRGLPWNLGLGHSISSTSCMCGNLEPILQQPENLEEENHEEGLEQGLSSYLRNAWWCLILGVLVCHKIYVSHSKARGERKEKVHLQEALAPKKQQQRAQTSSRGAGRWRKNILLLGILGGVSFSVWWFWDTNEEIIMKRRETLANMCDERARVLQDQFNVSLNHVHALSILVSTFHHGKIPSAIDQRTFEEYTERTNFERPLTSGVAYALKVPHSEREKFEKEHGWAIKKMETEDQTVVQDCVPENFDPAPIQDEYAPVIFAQETVSHIVSVDMMSGEEDRENILRARASGKGVLTSPFKLLKSNHLGVVLTFAVYDTSLPPDATEEQRVEATIGYLGASYDMPSLVEKLLHQLASKQTIAVDVYDTTNTSGLIKMYGSEIGDISEQHISSLDFGDPSRNHEMHCRFKHKLPIPWTAITPSILVLVITFLVGYILYEAINRIATVEEDCQKMRELKARAEAADIAKSQFLATVSHEIRTPMNGVLGMLKMLMDTDLDAKQMDYAQTAHGSGKDLTSLINEVLDQAKIESGRLELENVPFDMRFILDNVSSLLSGKANEKGIELAVYVSSQVPDVVVGDPSRFRQIITNLVGNSIKFTQERGHIFISVHLADEVKEPLTIEDAVLKQRLALGCSESGETVSGFPAVNAWGSWKNFKTCYSTESQNSDQIKLLVTVEDTGVGIPVDAQGRIFTPFMQADSSTSRTYGGTGIGLSISKRLVELMQGEMGFVSEPGIGSTFSFTGVFGKAETNTSITKLERFDLAIQEFTGLRALVIDNRNIRAEVTRYELRRLGISADIVSSLRMACTCCISKLENLAMILIDKDAWNKEEFSVLDELFTRSKVTFTRVPKIFLLATSATLTERSEMKSTGLIDEVVIKPLRMSVLICCLQETLVNGKKRQPNRQRRNLGHLLREKQILVVDDNLVNRRVAEGALKKYGAIVTCVESGKAALAMLKPPHNFDACFMDLQMPEMDGFEATRRVRELEREINKKIASGEVSAEMFCKFSSWHVPILAMTADVIQATHEECMKCGMDGYVSKPFEEEVLYTAVARFFEPC

>[ZmHK1](http://www.ncbi.nlm.nih.gov/gene/541634)(CKR) NC_024463

MGGKYRAARTKRWWRGLAAAGWVLTAVVCSAVMHWTLRRDSMDRAEERLVSMCEERARMLQEQFGVTVNHVHAIAILISTFNFEKSPPAIDQDTFAKYTARTSFERPLLNGVAFAQRVFHHEREMFESQQGWVMNTMQREPAPPQVEYAPVIFSQDTVSYLARIDMMSGEEDRENIFRARTTGKAVLTNPFRLLGSNHLGVVLTFAVYRPDLPADASVEQRVEATIGYLGGAFDVESLVENLLSKLAGNQDIVVNVYDVTNASDAMVLYGPSSLDEQVPFLHVSMLDFGDPFRKHEMRCRYRQKLPMPWSAITNPLGTFVIWMLLGYSIAAAYSRYDKVTEDCRKMEELKTQAEAADVAKSQFLATASHEIRTPMNGVLGMLDMLLGTDLTMTQKDYAQTAQMCGRALITLINDVLDRAKIEAGKLELEAVPFDLRSLMDDVVSLFSSKSREKCIELAVFVCDNVPKVVIGDPWRFRQILTNLVGNAVKFTERGHVFVRVCLAENSNMEANQVLHGAMNGKGGRVESTANGAFNTLSGFEAADRRNSWQYFKLLLSDKESLLDDLESENSNQSDSDRVTLAISIEDTGVGIPLQAQDRVFTPFMQADSSTSRNYGGTGIGLSISKCLAELMGGQISFTSHPSVGSTFTFSATLKHSHKDISGDSSRSLTEALPTAFKGMKAILVDGRPVRSAVTRYHLKRLGILLQVVNNMNAVVKAFPGQNGAAGSREKASILFIESDFWRPETDVQLLNHLREQKNGQLSDGHKVVLLVTSEADKDKYGSIFDIVMCKPIRASTIASSIQQLLKVEIAERKDNQNRPSFLRSLLVGKNILVVDDNKVNLRVAAAALKKYGANVSCVESGKDAISLLQPPHRFDACFMDVQMPEMDGFEATGQIRQMELKANEERKNKLASIEGSTTAEYHLPVLAMTADVIQATYEECIKSGMDGYVSKPFDEEQLYQAVSRLVVGTTDSAV

>PaCRE1(CKR) MA_47453g0010

MRLPSDMTCSLESKMLTQGNGFHGGGLVSSNIYQSCGLSRLALALKWWSQSTASNTKKQNPSKWPKLETKWHTKLFVLWVLVAAIVSMWIFYSMNYDIIERRKETLASMCDERARMLQDQFSVSMNHVHALAILVSTFHHRKHPSAIDQKTFAEYTARTAFERPLMSGVAYAHRVLHSEREQFEKQQGWTIKTMKSREPSPMQDEYAPAIFSQETISYVGSLDMMSGEEDHENIVRARATGKGVLTSPFRLLESNHLGVVLTFPVYYTDLPPDATPEERNRATAGYLGGAFDVESLVENLLRQLAGNRAIIVNVYDITNSSTPLIMYGPQVTDNVVPHVSNLDFGDPFRRHVMHCRFNENPAIPWSAITTSLGIFVIAVLVGHIIYAAVNRIAKVEEDFRKMEELKKQAEAADIAKSQFLATVSHEIRTPMNGVLGMLQMLMDTDLDATQQDYARTAQASGKALITLINEVLDQAKIESGKLELEEVPFDLRSILDDVLSLFSGKSRDKGIELAVFVSDQVPDIVVGDPGRFRQIITNLVGNSVKFTERGHICVCVHLAENVKAVREANVEACSKQLTEDKKQSLKSSCNTLSGFEAADGTNSWETFRLLLSDGIMNQQPGTIDKLSFPEASDTVNLVVSVEDTGVGIPMHAQRRVFMRFMQADSSTSRTYGGTGIGLSISKCLVELMSGEMNFKSRPGVGSTFTFTVVLKKAHTSQDIKRHQPESLPTIFKGMRAVVVDGKPVRAEITKYHLRRLGVRVEIANDQKSALAIISGGKNSDIRCRATGNIDMVLVDKEAWGPGRGLTFPQLLRESTLSSKAGSCPMLPKMILLATSINSIDSEKAKAIGFVETVIMKPLRASLIAACLQQALGIGDKRQQGKGLMDGSPSLQSLLLGKNILVVDDNRVNRRVAAGALKKYGAKVENAESGKAAIAMLQPPHKFDACFMDVQMPEMDGFEATRQVRNIERQVNEQIESGVTSREAYGDVDRWHVLILAMTADVIQATHEECLRCGMDGYVSKPFEEEQLYRAVAGFLDTKTKPTXAKLAFLVKVILLPPMSKLHANNPHELQSKEHVHSYSY

>[SmHK1](http://www.ncbi.nlm.nih.gov/protein/302768761/)(CKR) gi|302768761|

MSKQRHVKYFGEKYHGKLLVLWLILGLGAAVYINHSLTDSYIERRRETLANMCKERAWMLQDQFNSSMNHVRSLTALVTTFHLAKQPSALDQARQFFWTSFERPLMSGVAYAHKVEHWQRRAFEEEMGWSIKEMRSNAPRAQNLDEYAPTVLSQNTLAHLASVDMMSGEEDRENILRSRASGKGALTSPFRLLESDHLGVVLTFTVYKTDLPDDATPAQRIQATAGYVGGAFDFESLVENLLRQLSESQTIIVNVNDVTNKSNPLVMYGPNIPDGNEIEVCQLEFGDPFRKHEMRCRFNKEASVVWTAITTTFGIVVIVLLVAQIFYAAGNRIAKVEEDYRKMEDLKVRAESADVAKSQFLATVSHEIRTPMNGVLGMLQMLMDTELDSTQRDYAHTALESGKQLIKLINEVLDQAKIESGRMELETVPFKLRTILDDILTLFSAENKDKGIELAAYVSERVPDVVLGDPVRLHQIVTNLVGNSIKFTDRGHIFVSVHLEEDVKAAMSAQCEACSKQVTETQDHSDKLNTVDAAGSCSTLSGLEAADRRNNWETFKLILKQEKSRLTTHKQPVDTVKLVFSVEDTGVGIPVHAQERVFSPFMQADSSTSRNYGGTGIGLSISKCLVELMKGEIGFVSSLGVGTTFWFTVNFKVGDLEALEAAHSAECSKQQRAAHSSTPLTTQLSGLKALVVDGRPVRSEVTRYHLRRLGIQVDVASDVSSALAHQRSKNLDMILIDKDAWGSSTGLSYPSKIKEAGPTLAPKLVLLAFDDDEHKAKAAGFSDNVIKKPLRASYIANHLEKALGLDLKRRTRESSTPTSSSSDTSASINSGSGSSNNLHSLLTGKWILVVDDNKVNRRVAAGALQKYGARVECVESGRLAIEKLEPPHKFDACFMDIQMPEMDGFEATRRIRCFEKRGGSHVPILAMTADVIQATYDECRRCGMDDYVSKPFEEKQLYRAVAKFF

>[PpCHK4](http://www.ncbi.nlm.nih.gov/protein/162675438/)(CKR) gi|162675438| MEAGQSDPEALRWNRPGKKLRSLHRSSSACLRYGPIRVLVVVLGWIVACGVGAVLWRAALKFREQEFDLKCENRKEVLKSQVENNLNASFVIVGLLASVPEVSADMWVNFATRTLFLRPTVKRLVYVERVPAANRAAFERKWNTSILYINHSNVTEVRSANDTEYSPVVFETDDKVPKYLFVDAGAYPAYRSAIDAARDTGLFTLSPVTPRRDGTWQMGAYLAYYGPGRDYTSFSSTEARRQACRGYVGIVMNVTDVFHGVLSRFTDVDDMDVVAVFNVNSKLESFDSYNCSAAASSKSCAVPLFDPAGRASEFSKAAVTWEYGTQHFEVRCLPKRNLKLLALRAAIAWPLLMSLVVIFFSIIVYLVLKRMQAIEEEVRLMEKMNEDLNVAKLAAEAADKAKSNFLATVSHEIRTPMNGVIGMTNLLMGTNLTTQQLEYVKVVQASGNTLIALINDVLDLSKIEAGRMELESVAYDIRKEVDGVFLLFDDRAQQNKIELSMLVHDAVPNYIVGDPGRFHQILANVVSNALKFTREGNILVCVRAMRVSQGNKALFTAITIDQNSAVPGDVQPSQVLPDEPFSLVGSDGMKLYEDQDVAPRLSIQPGSESNRSEALDLWRKWEWKNLSGSHCKPPNQFILIVSVEDTGPGIPCHMEPRLFQPFSQADSNSSREHGGTGIGLFISQKLIKLMNGTIRVMSEPGKGSVFEFTLPASFADTAGGSKPQLPMEDKRLKGLHVALVDTDLVHREITASFLRYSGMHVELADDVQSTMEILQRTGGPTLQAVMVDIKGLPSAPAVELARSIRSTPSLKALSVLVLTSNPVSPPGEKELKDAGVSFIISKSLRLSTGSSVLLEAMGLKPQAPVKKKANDNVKLLLGKRLLVVDDNMVNQRVATSMLKRYGAIVSSVNSGIEDKKLDLVLMDIQMSEMDGWQATRHIRNWEVENCDTCCKSNVNWCRHNRLPIVAVTADAMKGTHAECFSSGMDDYITKPLDQKQLQSLLERFIKRDLVNVPPMTDVGS

>[MpCHK1](http://www.ncbi.nlm.nih.gov/protein/1026759120/)(CKR) gi|1026759120| MLCNSDDEERHRWRRGCRSLSHLKGLKRTFPSWKEIRQSPRRAVAAQLVCTAIGFLTAVGVGLVLFHNSLQTQRQEFKLKCSNRQEILMGEVANNLNTSFMILGLLASEPDLTQEAWLAFTNETDFLRPSTPRVSYIELITDKERAGFELEWNSSLLMIDEKQEAIPINYTAKEYAPIIYASQTVLYALLVDVRSFRAINHTLNNARNSGAIAMSPPDQYGTIWRVGTYLPYFGNKTPTTVEERIEMCIGWVGVSLDVEKVFGTVLSRYQDDEDMDAAVVYLPQSRDDWLPSLNCVPSASTCELPVYDPQSRFGQESTATIAWTYAFQNFELRCFATKSIRLNALRNVIAWPILMMIVVLLCSVIVYLAIKKMQAIEKHMFQVEKMNFDLRAAKQAAESADKAKSRFLATVSHEIRTPMNGVIGMTNLLMGTELSAQQHEYVKIAQASGNNLVSLINEVLDLSKIEAGKMELESVPFDLRVELDDLLCLFEDKVNEKKLEVSALVHDSVPRCVYGDPGRLRQVLINLVGNSMKFTKHGSIFVCVRIYNPQEDTFSSMLTSSLSSSPASEGSHRGKRQYDVSKLVKIVESREWSASDIRLEGVGVVEAMAPRLSMQEGPLNTEEAVKKWRNWVPKAGVEGEDSVEGSKTLSLVISVEDTGIGVPSHLQHRLFQPFLQADSSTSREFGGTGIGLSISKKLVELMGGKLDVISAPDEGSIFEFTFKVGKERDVADKDVKRECEGYGEENLKGRRVLLVDQHFVRQEVAASYLRRLGVIVEGVSKRQTALTSLLESDRPQIHAVILDLQGMGMDEAVQLVKLMRKEQHLMSIPVLALSCPLTTPLEKKELYEAGFSQTVFKPIRRTTLATGLLQAVGISLRPPTKTVNTNANMLAGKRLLVVDDNLINRKVARSMLARYGATVECVNGGVEAIEAIKNKAANLQFDLILMDIQMPEVDGCEATRRIRRWEIENCSFCRASESKKWEQQAPPGVLQQCPHSRIPVVAVTADVMQGTHEMCFGSGMDDYMPKVNQEEEVRHWKGNLWHNEQVNHKVTTRIKDA

>GrHHK7(CKR)

MQTIHPLVLTPHLSPIFTIFYALINISEFFLLYMPLQRWFFVKDIANSNCFYRNILHYVNGIFLVSNQMSDQTNRHSLSIRSTNSEGAEEKNSATLTTFAATEIPEKTNTISDEIPEKTNTIFDDAADLCFKGSNDVGGKGLQFEDRYVVNFYNTTDNTNAELCDNPEIANTDMPKNTRNGYYRNNSNTTTNTSSVKECFMNRIRPSPQTLSTIIIPIIIFLLGIIMGIAVFFVILGQERRRYRIEFGYYCDERKNAIVSGFWRSLATARDFCAFLSVTPNVTGAIVNRYGNFSSMLNMNIRSVNFAPRVKANDRLSWEKENGLIMKQLNNDGEVVTRDYNGTEYFPLQYISPWRSDSIKAIGFDVYSQIERRRSIEVLRKARNGNVTITSSIPLVFNASVSGVLVLFPFYKNLTDPHFIPPDRDLDGLIIGVYDINKSFGSIIDQFQDVGLSIKIIDRDYDSLIFDTSQPDVKYRNGSDGFMVEIDHKFADRYWTFQCVPSESSYNFAVSKTMPSVSLVLFTLFFGLLGFLTSRYFRKYLSARDKVSMQTRKLGETQSLLKAITADSKAVLEAIADPLIALNAKGEIVGANQHALRLTGYSPNDIKVQNKMHVNQLLIPIVETPAEEREISDFDPMQVPVRPGMRDVMARRKDGTCFEAEANFSQQVVEKNYCTQVVMFRDVSFKKEYERSVIEAKKEAEMANQSKTEFLFFLCHEIRNPIHAIFGFAEMLKNSFKEKEQEELDYIMSAGKFLSFIVNDILDLTHLTNPNPYEIELKCEPFELHALISNLAKIQSVAATNKKINIKTIIHSDIPRNIYGDARRIEQVINKLIARSIEIAPEGGIIELEIQALIFHSTRGVLLRFSVLDESEGLSSDKEIAELFKPYSKANSSIGSRFHAQGLSMALAQAIVKVMGGKLHVDKAQKKQPRNRVWFDIWLRTDDKFTWDNFKRGSFDVAISPTHSTDETHMDYSPDTTEFKAQLHASKSVSIAKSLPSVRPYKSTLRRKRRQRLSNGEESDDGNNNSIIKGNFFQRNGSVKATATGFGGAGSSHQNNTTVNDKESMNDSIDSITSFKPSPTFPPIITLYPGVKSQSDSENLNEDTTFTQKSISSYDNSLLQASPIKLKSDASIITPPRSPTLLNTQNATSDQPSVATDEHSNQNLYINIQKSPVQTIISSNNTSVTPPSSSHAAHPPLSTTSSDQTLITLTTLQTENSMVDDSNANVTSSIAPLSLSTSQTCQVEQSSPQTSKVLLVEDNLICQRVTSKMLVRNNYSVDIANNGKEAIDMVEETINMGGYSCILMDIITPVMNGYEATKLLRERGVDIPILALTANSFNSDFKKAIDVGMDAFLTKPIKEEELIVAIKEEIEKYKNRHHSLGSEEINNTIES

>[SPPG_01597](http://www.ncbi.nlm.nih.gov/protein/XP_016612202.1)(CKR) XP_016612202

MPTRRNGPSRRGSRAVSSEALTSRANRAVISSSSALSPDLSPVESGSTQSASAKPTGWRSSVNRTRETLSVHKPNIGMLLWILAGLAVSIPVLFALRNRSQDAERQAYRSDCQSTVAAIQNQMMLPLTLIYGYRWFFDSGVNVTQNVFQRFTKSYETQSWMVPWVGYAPYVTQNQRASWEATNGRPISQGPRDGESLPVSPTEGYVPVNRAVADTYFPTQYVPEGSEWLIGFDVLSDSTSQPAVFRALNTGLLTIGQKVQLRFENGYRDVHRFVLPWFGPNGTGRSLQGIFYGVFDPYQLIDTTVQSTTANISLGLQVLDASGGNAVLYAQPRNSTPSGQSMGYYSFPVADRTWILYCWGKPKQLPAPYVAFVFLLLTFLLLALLTRCCMLKRRQRDKSDPTIIENPRMSQRQIDWMRTNAYSILGAIRDPLLLINREGYVIDANDEGLALTKLVVEDLIFGIHISRVFPGVSTTKSRNGVKDKHFLKISHPPGPWASSTNIPAAVTGNDAVQEKDNVVIDVDSTYINLPDSTPRDEYNAATDDDMVIPGMQETVLKTKDGREITVEANFSPIVDCQDQKERIQMVLFRDMSGWKSIIKETLEAKDAAEMATTEKSNFLAFVCHELRNPLHVVIGLNSILMQSISSAADNGAMSPSSSDTPSPPPSTTELSDGNGGSKSNQAMTLSPEGLEHLASVADAARVMKFILNDMRLLSKIEAGTVEIERVPFDLKALTERMYKAQMACKLTNGLDDRGVCEAVRCSPDVDFKLVIKGLEDVPPVAEEADKTVELGSKEAPSNTSGKEWFPARVRGDATKLQQALLHLVSNSFESTKRGSVILRVIVEGIRPIPVDAIPQSPKPSEKRVSFREGLVDATSQVLVRFEVEDTGEGFPTTDLPNLLKKYSPESGADRAIGTSGLSLNITNSLLQLLGGEIQMASTPGKGTKVSFSLWFDLLDEISQQEDQETNAELKGLGIASSFGSGLPFGKENEGLVCRRESIAKNPGCKSTGIEQQITPIPDSVVISHDYARDSPGFADITSTDIQNRAPSPDSTPGGSPSQSTPPQASPSLSGVPSISPITRTSSMSRAQAVGRDPSIMHVASPRITLSSPNLTPGVRRSSQGDRKSPSIRPNGTKKSPNPAVRRLGTHPLKDRPIRVLVVEDNEVLLKIAATTLTRAGFEVQQAQNGEQAVQRIEKNGEKYFDVCLMDLLMPVMDGFQATEEIRRRGWTIPVIALTAKTLESDRLRCFKIGFNYFMTKPFQLGDIATVIRFMVGAEAEQQAQGPHDGATPHPNYGGPWSKFT

>[Bm|296463|](http://genome.jgi.doe.gov/cgi-bin/dispGeneModel?db=Basme2finSC&id=296463)(CKR)

MPIPAISRKGSHTGTTFFDILNDDPDEILENEKDTSYQEPQDCVHKPRKTRFQRYKLFLVCAAWFIVTSVVGGNLFYYSSRMDRQSMQEKLASNCQQSTNILKSTLSRLINDLSNHAYFVSSVGQGMTYEMFSTFTSYATKDSLKYALAWQPQVKQHEREEWEAQNGINITIVQETSSMLLKTPSPMQPYYYPILYGVMYDTEHIGIDVLQTNRSDTILKALGTLKTAVSPISEFKLMGFDGIRIYLPVLVNLYLTDKGDYSTFRNNHTKVHENVLGLVSLSVSIQNILTNAINISDGNYIRIIDKTGNDYMIYSNNGKEELPGDIFEYREELSFGDREWHISCYSPPLPFFPSKTKSSLFFAIILCTIFSTIILFILTKKYLKAKELVSIRSKMLQDSNALVTDMATNSKAILMSITDPLLLFNRDGKITGANAYALSRTGYTSDDISSSSELAIKDVINIAQWTGSVTRLVIEPGMREVTITCKNGSQFFAEANFSAQTVIGSNYAQVVTFRDISAKKQAAIDLWNAKNKAEKADKSKGDILLYLCHEIRNPVHAIGGYARLLMEQMEDEDEPSEEMENILCSSKFLSELVNEVLDLANLHDNDLVLNERTISLAELLATINHLTSPMEGSKYQLTSTLTGSGQVRVVCDPSALQLVIHRLGCLLSDSTSIDDHEEALMRVDVVLVKEDAECAIIKYLIQCPNKVIPSHYLAVDFDPLAQGNGSMGDQFGMKGISLTLTRIMIQKLGGKLISTSDTRIGTQFSFELQFKRSPDHPSSSSSSRPLHHTSQRALWMRIQALQRASHILRGQPPSSGPAHQRSPARRGNRSSHAQNQTRTGPAITEKSSRLLTKNGYDVTTANNGLEAVEAVERSPDYDIALLDIHMPLMGGLEAGRAMRERLGFQDR

>[AMAG_01137](http://www.ncbi.nlm.nih.gov/protein/909128066)(CKR) KNE55224

MSYGPVPTNPTAAAAAVNDATAPLPVSPRSHVASAPADTVVVTVHDPHHDHDVLPPPRRTASPPLDHVDDDDLRTPLAPATEDPGRRGRFRSMASPRAVPVPPPPTPPPSLPGGGDSPARPGSINSPADQSSERDEKVPPCCLSFWRHRHQFWAFLLALFVFLVGLAFSLVLFFWFHDMERSKFEHAVMDMCRAFRNALSIEFNVNNLNYLIDVAAFLPTLAVVTQEGISRFGNASPFPLNQIYTVLASPLVTSEAQDRYTTTYGISIWRESNIRPAPVVVPITFAFPDNITLIRERGFDLYTDPQRSTAVHGAFHGGKPSLSNPYPIFNNTETGMMLSLPVNMPRDPNFRTWTIGGTVVMSPVIKNSLTISGSKALIIDVYSGDGTHQWTVQCTSTDHFADPFFTPWPFVVLFAVLLIFTVMAEFVRRGFLRFLRMRRVTRQVQQRDRLVASLNTYSKAILQAVPDPLLVLDGLGYVIGLNERALVRLGLSESDLVHAHISDLVRGAPQLETLAPGTYEVGVLGKNEDTFFAEATVSRVVSQQRGQFAQVLLFHDITERIESLKALRAAEAAAQAAHRAKSQLLYFISHEMRNPIYVVENSVDCLALSEHPEIVQAVTTVKRCTMAMSDLLDNVMEFMNESKHPCYTQHRFATRVSDLLESTLVQFALPWVDAVRLEGCTCFLVKIIVKVLFITVQCAAPGSAVLVEAMVDEAGQFLLSHRVETVHDYRRFRVRRVRQESGRSVSSRVVLDAVEPDDDVEKGTLTLAFSVLSKLVARCSGTVHLFSQDGRGGLNVVLPLAVMNIKVLRRREPSGASHLVHGSSAVTGTHSAPPHSVSPVAEDQDERRPLPLIDVEDEIGGSVADWVHSTTSDRRSDRVPTMPRTVSMATSTSLGPVPRTMSTATSGSLGKVEPEAVGRDIVRRPSIPDVLADLVHPAANGVAAPSRPGHDATHIDTSHAMVQISHHGVDGLILLDAAHSRAAEARASPAELSNGHEPPCVNVEDDDVASLGRSDGGAQDRERLAPAADADLPDPTADTTVSETESHLPVPPDTATTHSHDFWTAPSSPAHRGLAASERPSEVDSPPVFTARTPALSPAELAAPRDVAEPPSTPQANGLRHDAPSIRSVPADVLKESRPPSAPVENPKSAPVSSRRFTLPMPPVRAHAAPSAALPPPVRVVQQSSASVPTQPVSSAATAAVAAGVLAGARATLFADGFARDAPDYINGESSPASTVHRDLHDHAGQTSPSPSSMSKRTGTSTQTAKPPPGSSSSNAPTPTAPPAPAPTVAPAATEKASAGPKRVLLAEDNLLVQKMTRKIVEKMGFTVETANDGAEAVTKSLTSDYDLILMDLVMPNKDGHEATQAIRAAGRSQASLPIIAVTANALPEERDRCLATGFNDFLTKPLKKEVLEAALAKVFPKDG

>[AMAG_18430](http://www.ncbi.nlm.nih.gov/protein/909133549)(CKR) KNE59867

MSYGPIPTNPTAAAAAAAAAAAAVNDATAPLPVSPRSHVASVPADAVVVTMHDPHQDALPPPRRTASPLLDHVDDDDARTPLAPATEDPGRRGRFRSMVAQRAAPVSPPTPPTPPPSLPGGDDGPARPGSINSPADQSSEQDNKMPPCCLSFWRHRHQVWAFLLALFVFLVGLAFSLVLFIWFHDMERSKFEHAVMDMCRAFRNALSIEFNVNNLNYLIDVAAFLPTLAVVTQEGISRFGKASPFPLNQIYAVLASPLVTTEAQDRYTTTYGISIWRESNIRAAPVVVPITFAFPDNITLIKERGFDLYSDPQRSSAIHGAFHGGKPSLSNPYPIFNNTETGIMLSLPVNMPRDPNFRTWAVGGTVVMSPVIKNSLTISGSKALIIDVISGDGTHVFREPYKNPQPYAVNVTAPIIDQQWTVQCTSTEQFADPFFTPWPFVVLFAVLLIFTVMAEFVRRGFLRFLRMRRVTRQVQQRDRLVASLNTYSKAILQAVPDPLLVLDGLGYVIGLNERALVRLGLSESDLVHAHISDLVRGAPQLETLAPGTYEVGVLGKNEDTFFAEATVSRVVSQQRGQFAQVLLFHDITERIESLKALRAAEAAAQAAHRAKSQLLYFISHEMRNPIYVVENSVDCLALSEHPEIVQTVTTVKRCTMAMSDLLDNVMEFMNESKHPCYTQHRFATRVSDLVESTLVQFALPVRERGLSITKWVDAVRLEGCTCFLVKIIVKFLFITVQCAAPGSAVLVEAMVDEAGQFLLAHRVETAHDYRRFRVRRVRQESGRYVSSRVVLDAVEPDDDVEKGTLTLAFSVLSKLVARCSGAVHLFSQDGRGGLNVVLPLAVMDIKARIPRRSAAYLRSPRTRTSCGRRR

>[DdDhkA](http://www.ncbi.nlm.nih.gov/protein/1136289/)(CKR) gi|1136289| MELKTFKDLNDDIIGDTSPVINTGDQPNPLRTQQQQLQQQQQQQQQQQQQQQQQQQQQQQQQQQQQHHIPQQLYQKQQQQQHSHSYGNHSFIHNVSPTSPSYDINNNNNNNNNNNNNNNNNNNNNNNSNNNNNNNNNNKNNYNNNYYYSPIENSNISKSLEESVLNQFPHNFNLNSSNNNYLNNSSSLHNINQSVNSLSNNNNNQTNQQPINNNNNNNNNNNNNNSNNSNNSNNNNNGNNNNNITDSPTKSKRHSTYETNIGSHQRRKSIQSLIANSAIHSFSKLKNKPLSSSTPSTVNTCGAVNNNSNNNNNNNNNSTGSLGAIPMDRSFDGNINTITEESTGGNNSPRSNCGSNCGSNGGIPLSPRNLSSLNSGVNVSPRNIHLNNLNNNSSNLPPLSPRHINFHINVSNLNNNNNNNINPNNNPNNSNNSNNNVSPRNNNHNISPRGSNISPRSNNGGSTTISPRNISNNNNIINNINNNNILTPPRNSPRLENVNPTNSPRLLATSLNSTLPIVSSLTSSNNNNQSNNNTNPSINNNNGRNGHCIQTISEEILGNKPVVYNNGNNNNNNNTNNSTTSNNNITTNNNNNNNNNINNNVLSTPRKRTKGNHSKTNSLQDFETSSMNGGDDSISGAGSGGSLRRRNKDDNDENDGNSNNTNSNNSNNNNNNNNNSSNNNNNNSNNNNNNNNNNNNNNNNNNNNNNNNNNNNNNNNNNNNNNNNNNNYHNGATMMMSHNHQSIGMSSSPKKNNFKPFSRNCSLMGMGRRAWAIILGLFIVGSSISILATLVLRYSEENSIADDFARVARDRFTMLRIEFNNRLYITQTLSLLLSVFPSTSEDQFVPFSKLWSDNAEGLEGIMWAPRVSNLDRYTWEIEHSVKIREIVTNPNNSSDMRDVPAAAASDYYPILFSEPQSSNDHFKGYNIYSDMWRRPSLNKTRDTGEKVSVASPYINKLANVPKNSRSNVLLYIYQAVYTYGKVLSTVEDRRHEVIGFASCRFFISRMVSASLQRLTEEDSLDLYVFDLDSTPIGELIYYRASNAGNDDGSSPTNIMNGKMLEDRSDMIYYNTMNVGGRNWMIALRPSRKFTNKHYTFYPYAIGGVCMLLSALVSFWFAVNTKHNIKLSATNEDLHKEIYNRKLAEKALAESQERLELAMEGSEDAVWDWKVNTGELHISSRWFQILKAHDTSYQSRTLYEELKSSSTNNLNFKGDSKNGGSNNGTFNLFKNGKVDSSSPQSITNVNTTNGGGGGELRKSNSGYLYNDELFSPIILEEMVSSPNTHQLAIWNMKFLAELIHPDDKQKFISEIKKTITRETAIMEIECRMRKKYGGYLYIIMRGKVVSNETSFKDNSLRMAGTLRDMTSRKDMQRLILEKEAAEEANKAKSAFVATVSHEVRTPLSGVIGVSDLLLETNLSEEQRDYVQTIQKSSQALLTIINDILDYSKLESRQLKMETLPFSIIETCQAVIHMLSVAANDDVDILLRVPPNVPRIIFGDAMRMRQVLLNRLSNAIKFTSRGHVLTDISVDDSIPPTNTEEEIIHLCITIEDTGIGIPQSLFDSIFEPFSQADNSTTRKYGGTGLGLSITKRLIEEVMGGTIQVSSIVGQGSKFKCIIPFLLPNTSPSDLNLISPSSLPKPFINRSPKSTYSFTDKKNSVPSTPIPSGDILINKVCLLICRDTVTELVFKEQLEWLGMIVKQVPRNVIDSIKNTILNNNNNNNNNNNNNNNNSNNSSSIISPSSLDYSDENEHLDLVLIDLEILTEHLKIPSNVPIIFITPTKFNISKHNGILNKWITKSPNQRVELIRRPAITDKLIPIISKCIKSQVQFTSGSSQLQSQQANLQQQLLHQQLCNNGQTLNNNYNSGGIGGGGGGGGSNTMNGSSGNLSNNNNFGQTPLSSGLVLLVHTGRTPPLFNNNGNSIIPPLELAVDHHGNQQQQLYQQQQQQQNNSSGNFQQFYQQQNNNSNNSFTPTLPNENSNNSIMNNSLNNNNTTPSNVTPTLFTSSPLDLQGRDTPVLQPPAYRKKALIVEDNELNRKVLAQLFKKIDWTISFAENGREALKEITGERCFDIVFMDCQMPVLDGFQTTKIIRSKERENNWKRMNIVALSAGSSSSFVQDCLDSGMDSFMGKPITLATLKDALAKWGGYNN

>[SyHik14](http://www.ncbi.nlm.nih.gov/protein/359277049/)(CKR) gi|359277049| (not included in the phylogenetic tree)

MVISATVISMKSPLRRLLLSPVLPILIGAGLSVMTVHTWLFLYNQVQERLKQVLTNRLTNIHEQIENQLNIHVRQLEQMAGRWERSPTGTEQTDWRLDARAQAEGFDGYQTIQWVDPKFVVRWVEPLPDDEHDIDYANQYPHQIQSLELAARQKNTILSGVVDLDQGEKGFLVYVPLFIGDRFDGFVVGVFQLDQFIGSVRPLSGNKPDQDSGLRSFGLRIFEGNQLIYNDVPDDWEQDIVVTQELAWANSLPQAEAMTSRWQLQLVPGPILLDQYGSSQWILFSGLLMAWAVAIAVYYLQKSSKHSEQLSESIKQQQKVEEFLKSTLQELAVQKTALDEAAIVAITDTEGVITYVNDKFVEVSGYSREELIGNTHRLVSSGYHSPEFFQQFWQTIRAGKVWHGQINNRAKAGNTYWVDSTVVPFLDDNGNPYQYLAIRFEITSSKQAEKSLRESEARFRMMADTSPIMLWVADQDKKMTFVNQSWLEFRGATLAEESGNGYLEGIHPDDKGHYLGVYSQAFGDRRRFELEYRYRRVDQQYRWIVNVGVPRYLEDGKFMGYVGSCLDITDRKQAQDILQKKLNQILLMRKISQEIRRSLQPTLIFQTAARQVGNVFAVSRCLIHNYSEATTLQVPVVAEYLGGQFTSLLAGEIAVEQAYDPTIIQGDRAMAVVDLDQDLNSTHTKAFYQRFQVKSFLAVRTSYQGKANGIIALHQCDRQRVWTADEIELLEAIAEQMGIALAQAALLEKERERRRELAQKNLELEKATWAAEAANRAKGEFLAMMSHEIRTPMNGVIGMTELLIMTDLNLQQLDYVQTIRQSGETLLTIINDILDFSKIEADKLVLETQAFELRPLIETVLEMFGPIARAKHLELTYGIDPQTPARILGDQVRLRQILSNLIGNALKFTEKGEVVLTVKGEPFDPAESYHTILNLPHPSHRICFNLRDTGIGIPLDRQDRLFKSFSQVDSSTTRKYGGTGLGLVISQRLTQMMGGVLTVTSEPGVGSNFRFCILTTAQAPALAEADSVQQMKGKQVLIVDDNETNRRILQDQCQAWGLVCHCFTSGESALDWFARCPDLDAAILDLQMPNMDGITLAHHLRQFAQGKDLPIILLSSGLVAGADELSVFQTVLNKPVRQSLIFDSLVNIFQGSIGLADYAPQFDQLDLPEFVPDGDGLPTEDNATSLQPALQILLAEDNLVNQKVAHQMLNNLGYPVAIANNGQEVIDALEKKFYDLVLMDMQMPVMDGITACRHIRQTLPLERQPRIVAMTANAMPGDRQECLDAGMDGYISKPISINQLRKVLQDTSALITSPQAREDIVTLGDKITVVEEQTMVKPTDVTESPLDPTAIAFLRDDLCGGDLTLFGEMVACYCQESQKLIEELVQGLEVDDFAVIRRTAHSLKSSSASLGAQQLSTFCQQLEKNAGSGNLGLGSPPQLVDRCRQLHIAVVEALAPFTIPSP
